# Fluorescent Probes to Image the K_Ca_3.1 Channel in Tumor Cells

**DOI:** 10.3390/pharmaceutics17020154

**Published:** 2025-01-23

**Authors:** Insa Thale, Elke Naß, Laura Vinnenberg, Luca Matteo Todesca, Thomas Budde, Ivan Maisuls, Cristian A. Strassert, Albrecht Schwab, Bernhard Wünsch

**Affiliations:** 1 Chemical Biology of Ion Channels (Chembion), University of Münster, Corrensstraße 48, D-48149 Münster, Germany; thalei@upmc.edu (I.T.); laura.vinnenberg@med.uni-duesseldorf.de (L.V.); lucamatteo.todesca@unipd.it (L.M.T.); tbudde@uni-muenster.de (T.B.); aschwab@uni-muenster.de (A.S.); 2 Institute of Pharmaceutical and Medicinal Chemistry, University of Münster, Corrensstraße 48, D-48149 Münster, Germany; 3 Institute of Physiology I, University of Münster, University Hospital Münster, Robert-Koch-Straße 27a, D-48149 Münster, Germany; nass@uni-muenster.de; 4 Institute of Physiology II, University of Münster, University Hospital Münster, Robert-Koch-Straße 27b, D-48149 Münster, Germany; 5 Department of Biology, University of Padua, Via U.Bassi 58/B, 35131 Padova, Italy; 6 Institute of Inorganic and Analytical Chemistry, University of Münster, CiMIC, SoN, Corrensstraße 28, D-48149 Münster, Germany; maisuls@uni-muenster.de (I.M.); ca.s@wwu.de (C.A.S.); 7 CeNTech, University of Münster, Heisenbergstraße 11, D-48149 Münster, Germany

**Keywords:** K_Ca_3.1 channel, non-small cell lung cancer cells A549-3R, senicapoc-derived small molecule imaging probes, structure-imaging property relationships, imaging of living cells

## Abstract

**Background/Objectives**: The Ca^2+^-activated K^+^ channel K_Ca_3.1 is not only involved in physiological processes such as immune reactions and control of vascular tone, but is highly expressed in various tumor entities. Thus, imaging of K_Ca_3.1 channels comes into focus for the localization of high channel density, i.e., for tumor diagnosis. In particular, the physicochemical properties of the fluorescent probes should be improved compared to existing probes. **Methods**: The small molecule inhibitor of the K_Ca_3.1 channel, senicapoc, was used as a warhead and was coupled with different fluorescent dyes. After synthesis of the novel probes, their physicochemical properties (lipophilicity, photophysical properties) and their ability to image K_Ca_3.1 channels in A549-3R lung tumor cells were determined. **Results**: In order to increase the polarity and quantum yield of reported fluorescent probes, three strategies were followed: (1) An F-atom at the B-atom of bodipy-labeled senicapoc derivatives 9a, 9b, and 15a was replaced by a OCH_3_ moiety, which decreased the logP value by one log-unit. (2) The *p*-phenylene moiety of the linker was replaced by an aliphatic tetramethylene linker decreasing the lipophilicity by 0.3–0.5 log-units. (3) Instead of bodipy dyes, fluorescein was coupled with the senicapoc warhead resulting in very polar probes 21a and 21b with low logP values of 1.5 and 1.3, respectively. Introduction of an ethyl moiety at the bodipy core increased the quantum yield, which resulted in the best punctate staining pattern of fixed and living A549-3R lung tumor cells with the ethylbodipy-labeled senicapoc derivative 10b. The specificity was shown by various control experiments. Co-staining with 10b and an antibody did not result in overlapping signals. **Conclusions**: The well-balanced lipophilicity and fluorescent quantum yield render the ethylbodipy-labeled senicapoc derivative 10b a very good probe to image selectively K_Ca_3.1 ion channels in fixed and living tumor cells. It was hypothesized that the antibody binds selectively at the closed channel (58.5%), whereas the senicapoc–bodipy conjugate **10b** binds selectively at the open channel (41.5%). The ratio 58.5:41.5 reflects the ratio of the ion channel in closed and open conformations.

## 1. Introduction

In 1958, Gardos observed that a rise of the intracellular Ca^2+^ concentrations can cause K^+^ diffusion across the plasma membrane [1]. This K^+^ conductance is mediated by K^+^ channels, which are activated by intracellular Ca^2+^ ions (K_Ca_ channels). These K_Ca_ channels are found in different cell types and tissues and are involved in various cellular (e.g., cell excitability) and tissue functions (e.g., heart rate regulation, hormone secretion) [1]. Based on single-channel conductance, the K_Ca_ ion channels are classified into three main categories: large-conductance K_Ca_1 (100–300 pS), intermediate-conductance K_Ca_3 (20–100 pS) and small-conductance K_Ca_2 (2–25 pS) ion channels [2,3,4,5].

Herein, we focus on the K_Ca_3.1 channel (also known as Gardos or SK4 channel), the only member of the group of intermediate-conductance Ca^2+^-activated K^+^ channels. It is primarily expressed in non-excitable cells such as erythrocytes, platelets, lymphocytes, secretory epithelia, vascular endothelial cells, vascular smooth muscle cells and fibroblasts [6,7,8,9,10] and is thus involved in physiological processes including immune reactions, control of vascular tone and renal fibroblast proliferation [11]. Activation of the K_Ca_3.1 channel has different effects on the specific cells or tissues. For example, opening of K_Ca_3.1 channels in erythrocytes leads to volume decrease; in secretory epithelial cells they are involved in salt and fluid secretion. It also provides the driving force for Ca^2+^ influx.

Furthermore, K_Ca_3.1 channels are overexpressed in various tumor entities, including cells derived from breast, prostate, lung (e.g., non-small cell lung cancer (NSCLC) cells) and pancreatic tumors [12]. In cancer cells, it contributes to proliferation, migration and invasion, metastasis and therapy resistance, behavioral traits underlying tumor aggressiveness [7,13,14,15,16]. Accordingly, increased expression of the K_Ca_3.1 channel correlates with tumor grade and metastatic potential [17]. Thus, the K_Ca_3.1 channel represents a promising target for the development of novel therapeutics and diagnostic tools [16].

The K_Ca_3.1 channel is a homotetrameric membrane protein. Each subunit consists of six transmembrane segments, termed S1–S6. At the C-terminal end of the transmembrane segment S6, two α-helices HA and HB represent the binding site for calmodulin, which is permanently bound to these HA and HB α-helices. Binding of Ca^2+^ to calmodulin causes conformational changes and movement of the transmembrane segment S6 resulting in opening of a pore between S5 and S6 for the passage of K^+^ ions [18,19].

**Figure 1:**
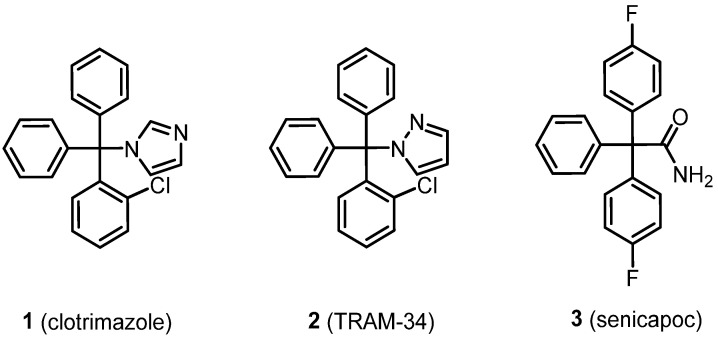
Development of prototypical K_Ca_3.1 inhibitors TRAM-34 (**2**) and senicapoc (**3**) starting from the antifungal drug clotrimazole (**1**).

A binding site for triarylmethane-based K_Ca_3.1 inhibitors is located within the channel pore. The first triarylmethane derivative, which was able to block the ion flux through the pore of the K_Ca_3.1 channel, was the antifungal drug clotrimazole (**1**) (Figure 1) However, the imidazole ring of **1** interacts with the Fe^2+^ of CYP P450 enzymes leading to undesired side effects. A shift of one N-atom from the 3- to the 2-position resulted in TRAM-34 (**2**), a pyrazole derivative, which is no longer able to interact with CYP P450 enzymes [20,21]. With an IC_50_ value of 20 nM, TRAM-34 (**2**) is approx. 5–10-fold more potent than clotrimazole (IC_50_ = 70−250 nM) to block K_Ca_3.1 channels [21]. The pyrazole moiety of TRAM-34 forms an H-bond with Thr250 and lipophilic interactions with Val275 in the S6 transmembrane segment within the ion channel. Binding at this region of the ion channel inhibits the passage of K^+^ ions before they reach the selectivity filter [21]. In 2003, the triarylacetamide senicapoc (**3**) with increased potency (IC_50_ = 11 nM) was introduced [21,22,23]. As TRAM-34, senicapoc (**3**) binds at Thr250 and Val375 in the channel pore inhibiting the permeation of K^+^ ions. In particular, the NH_2_ moiety of the primary amide of senicapoc acts as H-bond donor binding at the OH groups of Thr250 moieties located within different subunits [21,24]. It was shown that these K_Ca_3.1 inhibitors reach their binding site within the channel pore by penetration into the cell and subsequent entering the channel pore from the cytosol [25].

**Figure 2:**
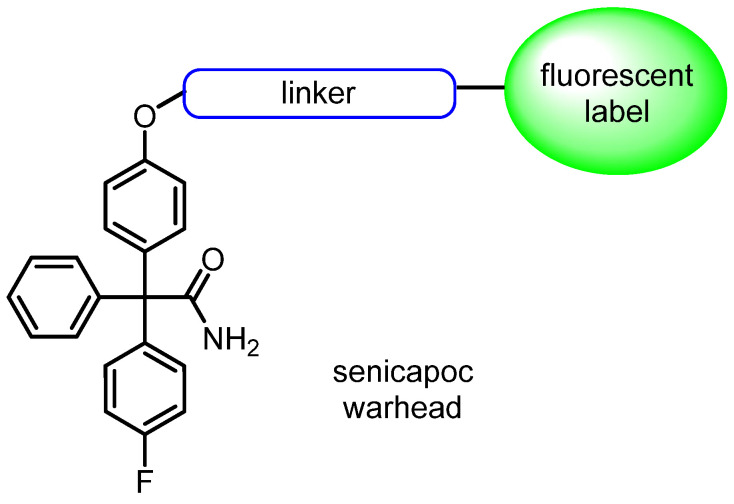
Concept of labeling K_Ca_3.1 channels: senicapoc warhead targeting K_Ca_3.1 channel connected with different fluorescent labels via different linkers.

Recently, we reported on bodipy-labeled senicapoc derivatives, which were able to image the K_Ca_3.1 channel resulting in punctate staining patterns of cells. Each fluorescent dot of the membrane corresponds to one ion channel [26,27]. In the present study, novel fluorescently labeled probes designed to image the K_Ca_3.1 channel are reported. For this purpose, a multidimensional optimization of the ligands regarding target interaction, polarity/lipophilicity, quantum yield and absorption and emission wave length are conducted. In particular, modifications of the bodipy framework or the introduction of alternative fluorophores increase the polarity of the fluorescent probe, while adjustments to the linker flexibility enable better adaption to the inner surface of the ion channel. Additionally, photophysical properties of the fluorophore are optimized (Figure 2).

## 2. Materials and Methods

### 2.1. Chemistry, General

Oxygen- and moisture-sensitive reactions were carried out under nitrogen, dried with silica gel with moisture indicator (orange gel, VWR, Darmstadt, Germany) and in dry glassware (Schlenk flask or Schlenk tube). All solvents were of analytical or technical grade quality. Thin-layer chromatography (tlc): tlc silica gel 60 F_254_ on aluminum sheets (VWR). Flash chromatography (fc): silica gel 60, 40–63 µm (VWR); parentheses include: diameter of the column (∅), length of the stationary phase (l), fraction size (v) and eluent. Automated flash chromatography: Isolera^TM^ Spektra One (Biotage, Uppsala, Sweden); parentheses include: cartridge size, flow rate, eluent, fractions size was always 20 mL. Melting point: melting point system MP50 (Mettler Toledo, Gießen, Germany), open capillary, uncorrected. MS: MicroTOFQII mass spectrometer (Bruker Daltonics, Bremen, Germany); deviations of the found exact masses from the calculated exact masses were 5 ppm or less; the data were analyzed with DataAnalysis^®^ (Bruker Daltonics). NMR: NMR spectra were recorded in deuterated solvents on Agilent DD2 400 MHz and 600 MHz spectrometers (Agilent, Santa Clara, CA, USA); chemical shifts (*δ*) are reported in parts per million (ppm) against the reference substance tetramethylsilane and calculated using the solvent residual peak of the undeuterated solvent; coupling constants are given with 0.5 Hz resolution; assignment of ^1^H and ^13^C NMR signals was supported by 2-D NMR techniques where necessary. IR: FT/IR IR Affinity^®^-1 spectrometer (Shimadzu, Düsseldorf, Germany) using ATR technique.

### 2.2. HPLC Methods for the Determination of the Purity

#### 2.2.1. HPLC Method 1

HPLC: Merck Hitachi Equipment; UV detector: L-7400; autosampler: L-7200; pump: L-7100; degasser: L-7614; column: LiChrospher^®^ 60 RP-select B (5 μm); LiChroCART^®^ 250–4 mm cartridge; flow rate: 1.0 mL/min; injection volume: 5.0 or 10 μL; detection at *λ* = 210 nm; solvents: A: water with 0.05% (*v*/*v*) trifluoroacetic acid; B: acetonitrile with 0.05% (*v*/*v*) trifluoroacetic acid: gradient elution: (A%): 0–4 min: 90%, 4–29 min: 90 → 0%, 29–31 min: 0%, 31–31.5 min: 0 → 90%, 31.5–40 min: 90%. Data acquisition: HSM software; manual integration.

#### 2.2.2. HPLC Method 2

An Ultimate system by Thermo Fisher Scientific (Bremen, Germany) was used to determine the purity of the synthesized compounds at a detection wavelength of 210 nm: guard column: Zorbax SB-Aq 12.5 × 4.6 mm cartridge; column: Zorbax SB-Aq StableBond analytical 150 × 4.6 mm; UV detector: UltiMate VWD-3400RS variable Wavelength Detector; autosampler: UltiMate 3000; pump: Ultimate LPG-3400SD; degasser: Ultimate AFC-3000: data acquisition: Chromeleon Client 6.80 (Dionex Corpor, Thermo Fisher Scientific (Bremen, Germany)). Data were evaluated by manual integration. Gradient was used to determine purities via HPLC; Tetrabutylammonium phosphate buffer (5 mM) in H_2_O/CH_3_CN, flow rate: 1.0 mL·min^−1^ at room temperature; 0 min: 20% CH_3_CN, 20 min: 80% CH_3_CN, 30 min: 20% CH_3_CN.

### 2.3. General Methods

General numbering of the bodipy core is presented as follows:

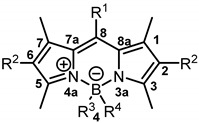



#### 2.3.1. General Procedure A for the Synthesis of Monomethoxy Bodipy Derivatives

Under N_2_ the respective bodipy dye (1.0 eq.) was dissolved in CH_2_Cl_2_ (140 mL/mmol). The solution was cooled down to 0 °C, and under stirring TMSOTf (5.0 eq.) was added dropwise. After stirring for 2.5 min at 0 °C, methanol (100 eq.) and DIPEA (10 eq.) were added to the solution. The mixture was allowed to warm up to room temperature, and H_2_O (140 mL/mmol) was added. The mixture was stirred for 20 min. The organic layer was separated and washed with H_2_O (3 × 140 mL/mmol), dried (Na_2_SO_4_), filtered and concentrated in vacuo. The crude product was purified as described in the particular procedure.

#### 2.3.2. General Procedure B for the Synthesis of 1,2,3-Triazoles

Alkynylated senicapoc derivative **8** (1.0 eq.) and the corresponding azide substituted bodipy dye (1.0 eq.) were dissolved in DMF (80 mL/mmol) and H_2_O (65 mL/mmol). Sodium ascorbate (6.6 eq.) and CuSO_4_ (6.5 eq.) were added, and the mixture was stirred at room temperature for 16 h. Afterwards, ethyl acetate (150 mL/mmol) and LiCl solution (5% wt in H_2_O, 150 mL/mmol) were added. The organic layer was separated and washed with LiCl solution (5% wt in H_2_O, 3 × 150 mL/mmol). The organic layer was dried (Na_2_SO_4_), filtered and concentrated in vacuo. The crude product was purified as mentioned.

### 2.4. Synthesis of Senicapoc Bodipy Conjugates Containing a Phenylene Moiety in the Linker

8-[4-(2-(2-Azidoethoxy)ethoxy)phenyl]-2,6-diethyl-4,4-difluoro-1,3,5,7-tetramethyl-4-bora-3a,4a-diaza-s-indacene (**6b**) was prepared as follows:

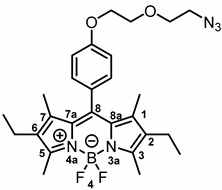



Under moisture, oxygen and light free conditions, 3-ethyl-2,4-dimethylpyrrole (**5b**, 1.72 mL, 12.8 mmol, 2.0 eq.) and benzaldehyde 4 (1.50 g, 6.37 mmol,1.0 eq.) were dissolved in dry CH_2_Cl_2_ (30 mL/mmol). Trifluoroacetic acid (2 drops) was added and the mixture was stirred at room temperature overnight. A solution of 2,3-dichloro-5,6-dicyano-1,4-benzoquinone (DDQ, 1.57 g, 6.37 mmol, 1.0 eq.) in CH_2_Cl_2_ (10 mL) was added and the mixture was stirred at room temperature for 30 min. Et_3_N (4.86 mL, 35.0 mmol, 5.5 eq.) was added and the mixture was stirred at room temperature for 15 min. After cooling the solution down to 0 °C, BF_3_ OEt_2_ (BF_3_ OEt_2_ (4.84 mL, 38.2 mmol, 6.0 eq.)) was added slowly and the mixture was stirred at room temperature overnight (16 h). H_2_O (30 mL/mmol) was added and the mixture was stirred at room temperature for 1 h. The organic layer was separated, washed with H_2_O (3 × 30 mL/mmol), dried (Na_2_SO_4_), filtered and concentrated in vacuo. The crude product was filtered over silica with CH_2_Cl_2_. The filtrate was concentrated in vacuo and the product was purified by flash chromatography (Ø = 5 cm, h = 15 cm, V = 30 mL, cyclohexane:ethyl acetate = 9:1), R_f_ = 0.21 (cyclohexane:ethyl acetate = 10:1). Red solid, mp 115 °C, yield 564 mg (17%). Purity (HPLC, method 2): 97.2% (t_R_ = 22.2 min). C_27_H_34_BF_2_N_5_O_2_ (M_r_ = 509.4). ^1^H NMR (600 MHz, DMSO-*d*_6_): δ (ppm) = 0.94 (t, *J* = 7.5 Hz, 6H, 2-CH_2_C*H*_3,_ 6-CH_2_C*H*_3_), 1.31 (s, 6H, 1-C*H*_3_, 7-C*H*_3(indacene)_), 2.29 (q, *J* = 7.5 Hz, 4H, 2-C*H*_2_CH_3,_ 6-C*H*_2_CH_3_), 2.43 (s, 6H, 3-C*H*_3_, 5-C*H*_3(indacene)_), 3.42–3.47 (m, 2H, OCH_2_C*H*_2_N_3_), 3.67–3.72 (m, 2H, OC*H*_2_CH_2_N_3_), 3.81–3.85 (m, 2H, OCH_2_C*H*_2_O), 4.16–4.21 (m, 2H, OC*H*_2_CH_2_O), 7.09–7.14 (m, 2H, 3-*H*_(Ph)_, 5-*H*_(Ph)_), 7.21–7.26 (m, 2H, 2-*H*_(Ph)_, 6-*H*_(Ph)_). ^13^C NMR (151 MHz, DMSO-*d*_6_): δ (ppm) = 11.5 (2C, 1-CH_3(indacene)_, 7-CH_3(indacene)_), 12.2 (2C, 3-CH_3(indacene)_, 5-CH_3(indacene)_), 14.5 (2C, 2-CH_2_*C*H_3(indacene),_ 6-CH_2_*C*H_3(indacene)_), 16.4 (2C, 2-*C*H_2_CH_3(indacene),_ 6-*C*H_2_CH_3(indacene)_), 49.9 (1C, OCH_2_*C*H_2_N_3_), 67.2 (1C, O*C*H_2_CH_2_O), 68.8 (1C, OCH_2_*C*H_2_O), 69.4 (1C, O*C*H_2_CH_2_N_3_), 115.1 (2C, C-3_(Ph)_, C-5_(Ph)_), 126.7 (1C, C-8_(indacene)_), 129.3 (2C, *C*-2_(Ph)_, *C*-6_(Ph)_), 130.4 (2C, C-8a_(indacene)_, C-7a_(indacene)_), 132.4 (2C, C-2_(indacene)_, C-6_(indacene)_), 138.1 (2C, C-1_(indacene)_, C-7_(indacene)_), 140.6 (1C, C-1_(Ph)_), 152.9 (2C, C-3_(indacene)_, C-5_(indacene)_), 159.0 (1C, C-4_(Ph)_). Exact mass (ESI): (*m*/*z*) = 510.2889 (calcd. 510.2847 C_27_H_34_BF_2_N_5_O_2_ [M + H]^+^). IR (neat): ṽ (cm^−1^) = 2963 (C-H, aliph), 2924 (C-H, aliph), 2110 (N=N=N), 1535 (C=C, arom), 1470 (C=C, arom), 1288 (C-O).

8-{4-[2-(2-Azidoethoxy)ethoxy]phenyl}-2,6-diethyl-4-fluoro-4-methoxy-1,3,5,7-tetramethyl-4-bora-3a,4a-diaza-s-indacene (**7b**) was prepared as follows:

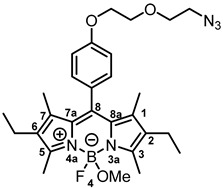



According to General Procedure A, bodipy dye **6b** (150 mg, 0.29 mmol, 1.0 eq.), TMSOTf (0.26 mL, 1.47 mmol, 5.0 eq.), methanol (1.20 mL, 29.4 mmol, 100 eq.) and DIPEA (0.51 mL, 2.94 mmol, 10 eq.) were reacted stepwise in CH_2_Cl_2_. The crude product was purified by automatic flash column chromatography; Column 1: (ø = 3 cm, h = 15 cm, V = 10 mL, CH_2_Cl_2_:CH_3_OH = 99:1), Column 2: (ø = 2 cm, h = 20 cm, V = 10 mL, CH_2_Cl_2_:MeOH = 98:2), R_f_ = 0.21 (CH_2_Cl_2_:CH_3_OH = 98:2). Red solid, mp 155 °C, yield 69.2 mg (46%). Purity (HPLC, Method 2): 90.7% (t_R_ = 20.7 min). C_28_H_37_BFN_5_O_3_ (M_r_ = 521.4). ^1^H NMR (600 MHz, DMSO-*d*_6_): δ (ppm) = 0.94 (t, *J* = 7.6 Hz, 6H, 2-CH_2_C*H*_3_, 6-CH_2_C*H*_3_), 1.30 (s, 6H, 1-C*H*_3(indacene)_, 7-C*H*_3(indacene)_), 2.29 (q, *J* = 7.6 Hz, 4H, 2-C*H*_2_CH_3,_ 6-C*H*_2_CH_3_), 2.42 (s, 6H, 3-C*H*_3(indacene)_, 5-C*H*_3(indacene)_), 2.78 (s, 3H, OC*H*_3_), 3.42–3.47 (m, 2H, OCH_2_C*H*_2_N_3_), 3.67–3.72 (m, 2H, OC*H*_2_CH_2_N_3_), 3.81–3.85 (m, 2H, OCH_2_C*H*_2_O), 4.16–4.20 (m, 2H, OC*H*_2_CH_2_O), 7.07–7.14 (m, 2H, 3-*H*_(Ph)_, 5-*H*_(Ph)_), 7.18–7.25 (m, 2H, 2-*H*_(Ph)_, 6-*H*_(Ph)_). ^13^C NMR (151 MHz, DMSO-*d*_6_): δ (ppm) = 11.5 (2C, 1-CH_3(indacene)_, 7-CH_3(indacene)_), 12.1 (d, *J* = 2.1 Hz, 2C, 3-CH_3(indacene)_, 5-CH_3(indacene)_), 14.6 (2C, 2-CH_2_*C*H_3,_ 6-CH_2_*C*H_3_), 16.5 (2C, 2-*C*H_2_CH_3,_ 6-*C*H_2_CH_3_), 48.4 (1C, OCH_3_), 49.9 (1C, OCH_2_*C*H_2_N_3_), 67.2 (1C, O*C*H_2_CH_2_O), 68.8 (1C, OCH_2_*C*H_2_O), 69.4 (1C, O*C*H_2_CH_2_N_3_), 115.0 (1C, C-3_(Ph)_), 115.1 (1C, C-5_(Ph)_), 127.2 (1C, C-8_(indacene)_), 129.4 (2C, *C*-2_(Ph)_, *C*-6_(Ph)_), 131.2 (2C, C-8a_(indacene)_, C-7a_(indacene)_), 132.1 (2C, C-2_(indacene)_, C-6_(indacene)_), 136.9 (2C, C-1_(indacene)_, C-7_(indacene)_), 140.4 (1C, C-1_(Ph)_), 152.9 (2C, C-3_(indacene)_, C-5_(indacene)_), 158.9 (1C, C-4_(Ph)_). Exact mass (ESI): (*m*/*z*) = 490.2812 (calcd. 490.2784 for C_28_H_37_BFN_5_O_3_ [M-OMe]^+^). IR (neat): ṽ (cm^−1^) = 2959 (C-H, aliph), 2929 (C-H, aliph), 2094 (N=N=N), 1539 (C=C, arom), 1462 (C=C, arom), 1111 (C-O).

2-{4-[(1-{2-[4-(2,6-Diethyl-4,4-difluoro-1,3,5,7-tetramethyl-4-bora-3a,4a-diaza-s-indacen-8-yl)phenoxy]ethoxyethyl}-1,2,3-triazol-4-yl)methoxy]phenyl}-2-(4-flluorophenyl)-2-phenylacetamide (**9b**) was prepared as follows:

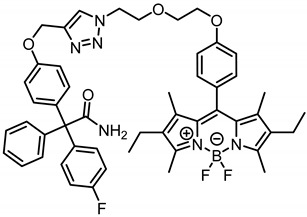



According to General Procedure B, bodipy dye **6b** (100 mg, 0.20 mmol, 1.0 eq.), propargyl ether **8** (70.6 mg, 0.20 mmol, 1.0 eq.), sodium ascorbate (257 mg, 1.30 mmol, 6.6 eq.) and CuSO_4_ (204 mg, 1.28 mmol, 6.5 eq.) were reacted in DMF and H_2_O. The crude product was purified by flash column chromatography, Column 1: (Ø = 2.5 cm, h = 15 cm, V = 10 mL, CH_2_Cl_2_:CH_3_OH = 99:1); automatic flash column chromatography; Column 1: (cartridge: SNAP KP-Sil, 25 g (Biotage^®^), ethyl acetate:cyclohexane, gradient 50:50 → 85:15, 25 mL/min), Column 2: (cartridge: SNAP KP-Sil, 25 g (Biotage^®^), ethyl acetate:cyclohexane, gradient 50:50 → 90:10, 25 mL/min), Column 3: (cartridge: SNAP C18, 12 g (Biotage^®^), CH_3_CN:H_2_O, gradient 60:40 → 100:0, 12 mL/min), R_f_ = 0.42 (CH_2_Cl_2_:CH_3_OH = 98:2). Red solid, mp 109 °C, yield 29.5 mg (18%). Purity (HPLC, Method 2): 99.2% (t_R_ = 22.1 min). C_50_H_52_BF_3_N_6_O_4_ (M_r_ = 868.8). ^1^H NMR (600 MHz, DMSO-*d*_6_): δ (ppm) = 0.92 (t, *J* = 7.6 Hz, 6H, 2-CH_2_C*H*_3,_ 6-CH_2_C*H*_3_), 1.29 (s, 6H, 1-C*H*_3(indacene)_, 7-C*H*_3(indacene)_), 2.27 (q, *J* = 7.5 Hz, 4H, 2-C*H*_2_CH_3,_ 6-C*H*_2_CH_3_), 2.42 (s, 6H, 3-C*H*_3(indacene)_, 5-C*H*_3(indacene)_), 3.76–3.81 (m, 2H, OC*H*_2_CH_2_O), 3.92 (t, *J* = 5.2 Hz, 2H, NCH_2_C*H*_2_O), 4.11–4.15 (m, 2H, OCH_2_C*H*_2_O), 4.59 (t, *J* = 5.2 Hz, 2H, NC*H*_2_CH_2_O), 5.11 (s, 2H, aryl-O-CH_2_), 6.60 (bs, 1H, N*H*_2_), 6.94–6.99 (m, 2H, 3-*H*_(OPh)_, 5-*H*_(OPh)_), 7.05–7.12 (m, 6H, 3-*H*_(FPh)_, 5-*H*_(FPh)_, 2-*H*_(OPh)_, 6-*H*_(OPh)_, 3-*H*_(dye-Ph)_, 5-*H*_(dye-Ph)_), 7.14–7.22 (m, 7H, 2-*H*_(dye-Ph)_, 6-*H*_(dye-Ph)_, 2-H_(Ph)_, 6-H _(Ph)_, 2-H_(FPh)_, 6-H_(FPh)_, 4-H_(Ph)_), 7.25–7.31 (m, 2H, 3-*H*_(Ph)_, 5-*H*_(Ph)_), 7.51 (bs, 1H, N*H*_2_), 8.24 (s, 1H, C*H*_(triazole)_). ^13^C NMR (151 MHz, DMSO-*d*_6_): δ (ppm) = 11.5 (2C, 1-CH_3(indacene)_, 7-CH_3(indacene)_), 12.2 (2C, 3-CH_3(indacene)_, 5-CH_3(indacene)_), 14.5 (2C, 2-CH_2_*C*H_3,_ 6-CH_2_*C*H_3_), 16.4 (2C, 2-*C*H_2_CH_3,_ 6-*C*H_2_CH_3_), 49.3 (1C, N*C*H_2_CH_2_O), 61.1 (1C, aryl-O-*C*H_2_), 65.9 (1C, *C*CONH_2_), 67.0 (1C, OCH_2_*C*H_2_O), 68.6 (1C, O*C*H_2_CH_2_O), 68.8 (1C, NCH_2_*C*H_2_O), 113.8 (2C, C-3_(OPh)_, C-5_(OPh)_), 114.2 (d, *J* = 21.2 Hz, 2C, C-3_(FPh)_, C-5_(FPh)_), 115.1 (2C, C-3_(dye-Ph)_, C-5_(dye-Ph)_), 124.9 (1C, C-5_(triazole)_), 126.4 (1C, C-4_(Ph)_), 126.8 (1C, C-8_(indacene)_), 127.7 (2C, C-3_(Ph)_, C-5_(Ph)_), 129.3 (2C, C-2_(dye-Ph)_, C-6_(dye-Ph)_), 129.9 (2C, C-2_(Ph)_, C-6_(Ph)_), 130.4 (2C, C-7a_(indacene)_, C-8a_(indacene)_), 131.2 (2C, C-2_(OPh)_, C-6_(OPh)_), 132.0 (d, *J* = 8.0 Hz, 2C, C-2_(FPh)_, C-6_(FPh)_), 132.4 (2C, C-2_(indacene)_, C-6_(indacene)_), 136.0 (1C, C-1_(OPh)_), 138.1 (2C, C-1_(indacene)_, C-7_(indacene)_), 140.3 (d, *J* = 3.45 Hz, 1C, C-1_(FPh)_), 140.6 (1C, C-1_(dye-Ph)_), 142.5 (1C, C-4_(triazole)_), 144.0 (1C, C-1_(Ph)_), 152.9 (2C, C-3_(indacene)_, C-5_(indacene)_), 156.5 (1C, C-4_(OPh)_), 158.9 (1C, C-4_(dye-Ph)_), 160.5 (d, *J* = 243.8 Hz, 1C, C-4_(FPh)_), 174.1 (1C, CONH_2_). Exact mass (ESI): (*m*/*z*) = 849.4120 (calcd. 849.4106 for C_50_H_52_BF_3_N_6_O_4_ [M-F]^+^). IR (neat): ṽ (cm^−1^) = 3496 (CON-H), 2933 (C-H, aliph), 1678 (C=O), 1543 (C=C, arom), 1504 (C=C, arom), 1189 (C-O).

2-{4-[(1-{2-[4-(2,6-Diethyl-4-fluoro-4-methoxy-1,3,5,7-tetramethyl-4-bora-3a,4a-diaza-s-indacen-8-yl)phenoxy]ethoxyethyl}-1,2,3-triazol-4-yl)methoxy]phenyl}-2-(4-flluorophenyl)-2-phenylacetamide (**10b**) was prepared as follows:

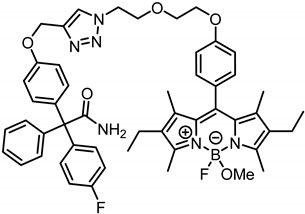



According to General Procedure B, bodipy dye **7b** (31.1 mg, 0.06 mmol, 1.0 eq.), propargyl ether **8** (21.4 mg, 0.06 mmol, 1.0 eq.), sodium ascorbate (77.9 mg, 0.39 mmol, 6.6 eq.) and CuSO_4_ (61.8 mg, 0.39 mmol, 6.5 eq.) were reacted in DMF and H_2_O. The crude product was purified by automatic flash column chromatography; Column 1: (cartridge: SNAP KP-Sil, 10 g (Biotage^®^), ethyl acetate:cyclohexane, gradient 50:50 → 85:15, 25 mL/min), Column 2: (cartridge: SNAP C18, 12 g (Biotage^®^), CH_3_CN:H_2_O, gradient 60:40 → 100:0, 12 mL/min), R_f_ = 0.45 (CH_2_Cl_2_:CH_3_OH = 95:5). Red solid, mp 106 °C, yield 15.7 mg (30%). Purity (HPLC, Method 2): 94.0% (t_R_ = 20.9 min). C_51_H_55_BF_2_N_6_O_5_ (M_r_ = 880.8). ^1^H NMR (600 MHz, DMSO-*d*_6_): δ (ppm) = 0.92 (t, *J* = 7.5 Hz, 6H, 2-CH_2_C*H*_3,_ 6-CH_2_C*H*_3_), 1.28 (s, 6H, 1-C*H*_3(indacene)_, 7-C*H*_3(indacene)_), 2.27 (q, *J* = 7.5 Hz, 4H, 2-C*H*_2_CH_3,_ 6-C*H*_2_CH_3_), 2.42 (s, 6H, 3-C*H*_3(indacene)_, 5-C*H*_3(indacene)_), 2.76 (s, 3H, OC*H*_3_), 3.76–3.81 (m, 2H, OC*H*_2_CH_2_O), 3.92 (t, *J* = 5.2 Hz, 2H, NCH_2_C*H*_2_O), 4.10–4.15 (m, 2H, OCH_2_C*H*_2_O), 4.59 (t, *J* = 5.2 Hz, 2H, NC*H*_2_CH_2_O), 5.11 (s, 2H, aryl-O-CH_2_), 6.60 (bs, 1H, N*H*_2_), 6.93–6.99 (m, 2H, 3-*H*_(OPh)_, 5-*H*_(OPh)_), 7.03–7.12 (m, 6H, 3-*H*_(FPh)_, 5-*H*_(FPh)_, 2-*H*_(OPh)_, 6-*H*_(OPh)_, 3-*H*_(dye-Ph)_, 5-*H*_(dye-Ph)_), 7.14–7.25 (m, 7H, 2-*H*_(dye-Ph)_, 6-*H*_(dye-Ph)_, 2-H_(Ph)_, 6-H_(Ph)_, 2-H_(FPh)_, 6-H_(FPh)_, 4-H_(Ph)_), 7.25–7.31 (m, 2H, 3-*H*_(Ph)_, 5-*H*_(Ph)_), 7.51 (bs, 1H, N*H*_2_), 8.24 (s, 1H, C*H*_(triazole)_). ^13^C NMR (151 MHz, DMSO-*d*_6_): δ (ppm) = 11.5 (2C, 1-CH_3(indacene)_, 7-CH_3(indacene)_), 12.1 (d, *J* = 1.8 Hz, 2C, 3-CH_3(indacene)_, 5-CH_3(indacene)_), 14.6 (2C, 2-CH_2_*C*H_3_, 6-CH_2_*C*H_3_), 16.5 (2C, 2-*C*H_2_CH_3,_ 6-*C*H_2_CH_3_), 48.5 (d, *J* = 6.4 Hz, 1C, OCH_3_), 49.3 (1C, N*C*H_2_CH_2_O), 61.1 (1C, aryl-O-*C*H_2_), 65.9 (1C, *C*CONH_2_), 67.0 (1C, OCH_2_*C*H_2_O), 68.6 (1C, O*C*H_2_CH_2_O), 68.8 (1C, NCH_2_*C*H_2_O), 113.8 (2C, C-3_(OPh)_, C-5_(OPh)_), 114.2 (d, *J* = 21.3 Hz, 2C, C-3_(FPh)_, C-5_(FPh)_), 115.0 (1C, C-3_(dye-Ph)_), 115.1 (1C, C-5_(dye-Ph)_), 124.9 (1C, C-5_(triazole)_), 126.4 (1C, C-4_(Ph)_), 127.2 (1C, C-8_(indacene)_), 127.7 (2C, C-3_(Ph)_, C-5_(Ph)_), 129.4 (bs, 2C, C-2_(dye-Ph)_, C-6_(dye-Ph)_), 129.9 (2C, C-2_(Ph)_, C-6_(Ph)_), 131.1–131.2 (m, 4C, 2C, C-2_(OPh)_, C-6_(OPh)_, C-8a_(indacene)_, C-7a_(indacene)_), 132.0 (d, *J* = 8.7 Hz, 4C, C-2_(FPh)_, C-6_(FPh)_, C-2_(indacene)_, C-6_(indacene)_), 136.0 (1C, C-1_(OPh)_), 140.3 (d, *J* = 3.0 Hz, 1C, C-1_(FPh)_), 140.4 (1C, C-1_(dye-Ph)_), 142.5 (1C, C-4_(triazole)_), 144.0 (1C, C-1_(Ph)_), 152.9 (2C, C-3_(indacene)_, C-5_(indacene)_), 156.5 (1C, C-4_(OPh)_), 158.8 (1C, C-4_(dye-Ph)_), 160.6 (d, *J* = 143.9 Hz, 1C, C-4_(FPh)_), 174.1 (1C, CONH_2_). Exact mass (ESI): (*m*/*z*) = 849.4123 (calcd. 849.4111 for C_51_H_55_BF_2_N_6_O_5_ [M-OMe]^+^). IR (neat): ṽ (cm^−1^) = 3495 (CON-H), 2945 (C-H, aliph), 1678 (C=O), 1605 (C=C), 1543 (C=C, arom), 1504 (C=C, arom), 1184 (C-O).

### 2.5. Synthesis of Senicapoc Bodipy Conjugates Containing an Aliphatic Linker

8-(4-Chlorobutyl)-4,4-difluoro-1,3,5,7-tetramethyl-4-bora-3a,4a-diaza-s-indacene (**12a**) [28] was prepared as follows:

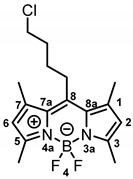



Under moisture, oxygen and light free conditions, 5-chlorovaleryl chloride (**11**, 0.9 mL, 6.97 mmol, 1.0 eq.) was dissolved in dry CH_2_Cl_2_ (25 mL/mmol) and the solution was cooled down to 0 °C. 2,4-Dimethylpyrrole (**5a**, 1.50 mL, 15.0 mmol, 2.2 eq.) was slowly added to the mixture. After stirring for 30 min at 0 °C, the mixture was allowed to warm up to room temperature and was stirred for another 30 min. Et_3_N (2.90 mL, 20.9 mmol, 3.0 eq.) was added slowly and the mixture was stirred at room temperature for 15 min. After cooling the solution down to 0 °C, BF_3_ OEt_2_ (4.40 mL, 34.8 mmol, 6.0 eq.) was added slowly and the mixture was allowed to warm up to room temperature. After stirring for 14 h, H_2_O (30 mL/mmol) was added and the mixture was stirred at room temperature for 15 min. The organic layer was separated, washed with H_2_O (3 × 30 mL/mmol), dried (Na_2_SO_4_), filtered and concentrated in vacuo. The crude product was filtered over silica with CH_2_Cl_2_. The filtrate was concentrated in vacuo and the residue was purified by automatic flash column chromatography (cartridge: SNAP KP-Sil, 50 g (Biotage^®^), CH_2_Cl_2_:cyclohexane, gradient 20:80 → 55:45, 50 mL/min), R_f_ = 0.51 (cyclohexane:ethyl acetate = 4:1). Orange solid, mp 164 °C, yield 1.28 g (54%). Purity (HPLC, method 2): 84.2% (t_R_ = 19.0 min). C_17_H_22_BClF_2_N_2_ (M_r_ = 338.6). ^1^H NMR (500 MHz, CDCl_3_): δ (ppm) = 1.76–1.84 (m, 2H, CCH_2_C*H*_2_CH_2_CH_2_Cl), 1.88–2.03 (m, 2H, CCH_2_CH_2_C*H*_2_CH_2_Cl), 2.42 (s, 6H, 1-C*H*_3(indacene)_, 7-C*H*_3(indacene)_), 2.52 (s, 6H, 3-C*H*_3(indacene)_, 5-C*H*_3(indacene)_), 2.94–3.01 (m, 2H, CC*H*_2_CH_2_CH_2_CH_2_Cl), 3.58 (t, *J* = 6.3 Hz, 2H, CCH_2_CH_2_CH_2_C*H*_2_Cl), 6.06 (s, 2H, 2-C*H*_(indacene)_, 6-C*H*_(indacene)_). ^13^C NMR (126 MHz, CDCl_3_): δ (ppm) = 14.6 (2C, 1-CH_3(indacene)_, 7-CH_3(indacene)_), 16.5 (2C, 3-CH_3(indacene)_, 5-CH_3(indacene)_), 27.7 (1C, C*C*H_2_CH_2_CH_2_CH_2_Cl), 29.1 (1C, CCH_2_*C*H_2_CH_2_CH_2_Cl), 33.1 (1C, CCH_2_CH_2_*C*H_2_CH_2_Cl), 44.4 (1C, CCH_2_CH_2_CH_2_*C*H_2_Cl), 121.9 (2C, C-2_(indacene)_, C-6_(indacene)_), 131.5 (2C, C-8a_(indacene)_, C-7a_(indacene)_), 140.4 (2C, C-1_(indacene)_, C-7_(indacene)_), 145.5 (1C, C-8_(indacene)_), 154.2 (2C, C-3_(indacene)_, C-5_(indacene)_). Exact mass (ESI): (*m*/*z*) = 339.1547 (calcd. 339.1606 C_17_H_22_BClF_2_N_2_ [M + H]^+^). IR (neat): ṽ (cm^−1^) = 2980 (C-H, aliph), 1547 (C=C, arom), 1504 (C=C, arom), 1304 (C-N), 714 (C-Cl).

8-(4-Chlorobutyl)-2,6-diethyl-4,4-difluoro-1,3,5,7-tetramethyl-4-bora-3a,4a-diaza-s-indacene (**12b**) was prepared as follows:

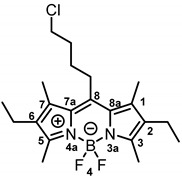



Under moisture, oxygen and light free conditions, 5-chlorovaleryl chloride (**11**, 0.33 mL, 2.59 mmol, 1.0 eq.) was dissolved in dry CH_2_Cl_2_ (25 mL/mmol) and the solution was cooled down to 0 °C. 3-Ethyl-2,4-dimethylpyrrole (**5b**, 0.75 mL, 5.56 mmol, 2.2 eq.) was slowly added to the mixture. After stirring for 30 min at 0 °C, the mixture was allowed to warm up to room temperature and was stirred for another 30 min. Et_3_N (1.10 mL, 7.77 mmol, 3.0 eq.) was added slowly and the mixture was stirred at room temperature for 15 min. After cooling the solution down to 0 °C, BF_3_ OEt_2_ (1.60 mL, 13.0 mmol, 5.0 eq.) was added slowly and the mixture was allowed to warm up to room temperature. After stirring for 14 h, H_2_O (30 mL/mmol) was added and the mixture was stirred at room temperature for 15 min. The organic layer was separated, washed with H_2_O (3 × 30 mL/mmol), dried (Na_2_SO_4_), filtered and concentrated in vacuo. The crude product was filtered over silica with CH_2_Cl_2_. The filtered solution was concentrated in vacuo and the product was purified by automatic flash column chromatography (cartridge: SNAP KP-Sil, 50 g (Biotage^®^), CH_2_Cl_2_:cyclohexane, gradient 30:70 → 60:40, 50 mL/min), R_f_ = 0.65 (cyclohexane:ethyl acetate = 4:1). Red solid, mp 111 °C, yield 572 mg (56%). Purity (HPLC, Method 2): 73.0% (t_R_ = 21.7 min). C_21_H_30_BClF_2_N_2_ (M_r_ = 394.7). ^1^H NMR (600 MHz, DMSO-*d*_6_): δ (ppm) = 1.05 (t, *J* = 7.6 Hz, 6H, 2-CH_2_C*H*_3(indacene),_ 6-CH_2_C*H*_3(indacene)_), 1.77–1.84 (m, 2H, CCH_2_C*H*_2_CH_2_CH_2_Cl), 1.94–1.99 (m, 2H, CCH_2_CH_2_C*H*_2_CH_2_Cl), 2.34 (s, 6H, 1-C*H*_3(indacene)_, 7-C*H*_3(indacene)_), 2.38–2.43 (m, 4H, 2-C*H*_2_CH_3(indacene)_, 6-C*H*_2_CH_3(indacene)_), 2.50 (s, 6H, 3-C*H*_3(indacene)_, 5-C*H*_3(indacene)_), 2.93–3.10 (m, 2H, CC*H*_2_CH_2_CH_2_CH_2_Cl), 3.59 (t, *J* = 6.3 Hz, 2H, CCH_2_CH_2_CH_2_C*H*_2_Cl). ^13^C NMR (101 MHz, CDCl_3_): δ (ppm) = 12.6 (2C, 3-CH_3(indacene)_, 5-CH_3(indacene)_), 13.5 (2C, 1-CH_3(indacene)_, 7-CH_3(indacene)_), 15.0 (2C, 2-CH_2_*C*H_3(indacene),_ 6-CH_2_*C*H_3(indacene)_), 17.3 (2C, 2-*C*H_2_CH_3(indacene)_, 6-*C*H_2_CH_3(indacene)_), 27.8 (1C, C*C*H_2_CH_2_CH_2_CH_2_Cl), 29.0 (1C, CCH_2_*C*H_2_CH_2_CH_2_Cl), 33.1 (1C, CCH_2_CH_2_*C*H_2_CH_2_Cl), 44.5 (1C, CCH_2_CH_2_CH_2_*C*H_2_Cl), 131.0 (2C, C-8a_(indacene)_, C-7a_(indacene)_), 132.9 (2C, C-2_(indacene)_, C-6_(indacene)_), 135.7 (2C, C-1_(indacene)_, C-7_(indacene)_), 143.8 (1C, C-8_(indacene)_), 152.5 (2C, C-3_(indacene)_, C-5_(indacene)_). Exact mass (ESI): (*m*/*z*) = 394.2104 (calcd. 394.2159 C_21_H_30_BClF_2_N_2_ [M]^+^). IR (neat): ṽ (cm^−1^) = 2962 (C-H, aliph), 1543 (C=C, arom), 1477 (C=C, arom), 721 (C-Cl).

8-(4-Azidobutyl)-4,4-difluoro-1,3,5,7-tetramethyl-4-bora-3a,4a-diaza-s-indacene (**13a**) [29] was prepared as follows:

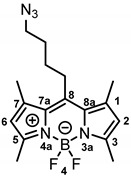



Alkyl chloride **12a** (800 mg, 2.36 mmol, 1.0 eq.) and NaN_3_ (306 mg, 4.72 mmol, 2.0 eq.) were dissolved in DMF (50 mL). The mixture was heated to 40 °C and stirred for 24 h. LiCl solution (5% wt in H_2_O, 50 mL) was added to the mixture. After stirring for 15 min, the mixture was extracted with ethyl acetate (3 × 50 mL). The organic ethyl acetate phase was washed with LiCl solution (5% wt in H_2_O, 3 × 150 mL). The organic layer was separated, dried (Na_2_SO_4_), concentrated in vacuo and purified by automatic flash column chromatography (cartridge: SNAP KP-Sil, 50 g (Biotage^®^), CH_2_Cl_2_:cyclohexane, gradient 35:65 → 60:40, 50 mL/min), R_f_ = 0.49 (cyclohexane:ethyl acetate = 4:1). Orange solid, mp 129 °C, yield 714 mg (88%). Purity (HPLC, method 2): 97.2% (t_R_ = 18.9 min). C_17_H_22_BF_2_N_5_ (M_r_ = 345.2). ^1^H NMR (600 MHz, DMSO-*d*_6_): δ (ppm) = 1.57–1.67 (m, 2H, CCH_2_C*H*_2_CH_2_CH_2_N_3_), 1.69–1.78 (m, 2H, CCH_2_CH_2_C*H*_2_CH_2_N_3_), 2.40 (s, 6H, 1-C*H*_3(indacene)_, 7-C*H*_3(indacene)_), 2.42 (s, 6H, 3-C*H*_3(indacene)_, 5-C*H*_3(indacene)_), 2.92–3.00 (m, 2H, CC*H*_2_CH_2_CH_2_CH_2_N_3_), 3.43 (t, *J* = 6.6 Hz, 2H, CCH_2_CH_2_CH_2_C*H*_2_N_3_), 6.24 (s, 2H, 2-C*H*_(indacene)_, 6-C*H*_(indacene)_). ^13^C NMR (151 MHz, DMSO-*d*_6_): δ (ppm) = 14.1 (2C, 1-CH_3(indacene)_, 7-CH_3(indacene)_), 15.8 (2C, 3-CH_3(indacene)_, 5-CH_3(indacene)_), 27.2 (1C, C*C*H_2_CH_2_CH_2_CH_2_N_3_), 28.3 (1C, CCH_2_*C*H_2_CH_2_CH_2_N_3_), 28.6 (1C, CCH_2_CH_2_*C*H_2_CH_2_N_3_), 50.1 (1C, CCH_2_CH_2_CH_2_*C*H_2_N_3_), 121.7 (2C, C-2_(indacene)_, C-6_(indacene)_), 130.7 (2C, C-8a_(indacene)_, C-7a_(indacene)_), 140.8 (2C, C-1_(indacene)_, C-7_(indacene)_), 146.2 (1C, C-8_(indacene)_), 153.2 (2C, C-3_(indacene)_, C-5_(indacene)_). Exact mass (ESI): (*m*/*z*) = 346.2032 (calcd. 346.2014 C_17_H_22_BF_2_N_5_ [M + H]^+^). IR (neat): ṽ (cm^−1^) = 2982 (C-H, aliph), 2928 (C-H, aliph), 2091 (N=N=N), 1550 (C=C, arom), 1304 (C-N).

8-(4-Azidobutyl)-2,6-diethyl-4,4-difluoro-1,3,5,7-tetramethyl-4-bora-3a,4a-diaza-s-indacene (**13b**) [30] was prepared as follows:

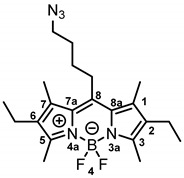



Alkyl chloride **12b** (500 mg, 1.27 mmol, 1.0 eq.) and NaN_3_ (165 mg, 2.53 mmol, 2.0 eq.) were dissolved in DMF (30 mL). The mixture was heated to 40 °C and stirred for 24 h. LiCl solution (5% wt in H_2_O, 30 mL) was added to the mixture. After stirring for 15 min, the mixture was extracted with ethyl acetate (3 × 30 mL). The organic ethyl acetate phase was washed with LiCl solution (5% wt in H_2_O, 3 × 90 mL). The organic layer was separated, dried (Na_2_SO_4_), concentrated in vacuo and purified by automatic flash column chromatography (cartridge: SNAP KP-Sil, 50 g (Biotage^®^), ethyl acetate:cyclohexane, gradient 0:100 → 15:85, 50 mL/min), R_f_ = 0.61 (cyclohexane:ethyl acetate = 4:1). Red solid, mp 124 °C, yield 256 mg (50%). Purity (HPLC, method 2): 94.1% (t_R_ = 21.6 min). C_21_H_30_BF_2_N_5_ (M_r_ = 401.3). ^1^H NMR (600 MHz, DMSO-*d*_6_): δ (ppm) = 1.00 (t, *J* = 7.5 Hz, 6H, 2-CH_2_C*H*_3(indacene)_, 6-CH_2_C*H*_3(indacene)_), 1.58–1.66 (m, 2H, CCH_2_C*H*_2_CH_2_CH_2_N_3_), 1.74 (quint, *J* = 7.0 Hz, 2H, CCH_2_CH_2_C*H*_2_CH_2_N_3_), 2.34 (s, 6H, 1-C*H*_3(indacene)_, 7-C*H*_3(indacene)_), 2.37–2.42 (m, 10H, 2-C*H*_2_CH_3(indacene)_, 6-C*H*_2_CH_3(indacene),_ 3-C*H*_3(indacene)_, 5-C*H*_3(indacene)_), 2.97–3.03 (m, 2H, CC*H*_2_CH_2_CH_2_CH_2_N_3_), 3.44 (t, *J* = 6.7 Hz, 2H, CCH_2_CH_2_CH_2_C*H*_2_N_3_). ^13^C NMR (151 MHz, DMSO-*d*_6_): δ (ppm) = 12.1 (2C, 3-CH_3(indacene)_, 5-CH_3(indacene)_), 12.8 (2C, 1-CH_3(indacene)_, 7-CH_3(indacene)_), 14.7 (2C, 2-CH_2_*C*H_3,_ 6-CH_2_*C*H_3(indacene)_), 16.5 (2C, 2-*C*H_2_CH_3(indacene)_, 6-*C*H_2_CH_3(indacene)_), 27.2 (1C, C*C*H_2_CH_2_CH_2_CH_2_N_3_), 28.3 (1C, CCH_2_*C*H_2_CH_2_CH_2_N_3_), 28.6 (1C, CCH_2_CH_2_*C*H_2_CH_2_N_3_), 50.1 (1C, CCH_2_CH_2_CH_2_*C*H_2_N_3_), 130.1 (2C, C-8a_(indacene)_, C-7a_(indacene)_), 132.4 (2C, C-2_(indacene)_, C-6_(indacene)_), 136.2 (2C, C-1_(indacene)_, C-7_(indacene)_), 144.6 (1C, C-8_(indacene)_), 151.5 (2C, C-3_(indacene)_, C-5_(indacene)_). Exact mass (ESI): (*m*/*z*) = 402.2654 (calcd. 402.2640 C_21_H_30_BF_2_N_5_ [M + H]^+^). IR (neat): ṽ (cm^−1^) = 2980 (C-H, aliph), 2933 (C-H, aliph), 2107 (N=N=N), 1563 (C=C, arom), 1190 (C-N).

8-(4-Azidobutyl)-4-fluoro-4-methoxy-1,3,5,7-tetramethyl-4-bora-3a,4a-diaza-s-indacene (**14a**) was prepared as follows:

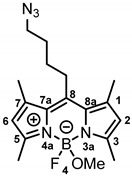



According to General Procedure A, bodipy dye **13a** (200 mg, 0.58 mmol, 1.0 eq.), TMSOTf (0.53 mL, 2.90 mmol, 5.0 eq.), methanol (2.30 mL, 57.9 mmol, 100 eq.) and DIPEA (1.0 mL, 5.79 mmol, 10 eq.) were reacted stepwise in CH_2_Cl_2_. The crude product was purified by automatic flash column chromatography (cartridge: SNAP KP-Sil, 25 g (Biotage^®^), ethyl acetate:cyclohexane, gradient 5:95 → 20:80, 25 mL/min), R_f_ = 0.24 (ethyl acetate:cyclohexane = 1:4). Orange solid, mp 93 °C, yield 128 mg (62%). Purity (HPLC, method 2): 99.7% (t_R_ = 17.0 min). C_18_H_25_BFN_5_O (M_r_ = 357.2). ^1^H NMR (600 MHz, DMSO-*d*_6_): δ (ppm) = 1.58–1.67 (m, 2H, CCH_2_C*H*_2_CH_2_CH_2_N_3_), 1.73 (quint, *J* = 7.0 Hz, 2H, CCH_2_CH_2_C*H*_2_CH_2_N_3_), 2.39 (s, 6H, 3-C*H*_3(indacene)_, 5-C*H*_3(indacene)_), 2.42 (s, 6H, 1-C*H*_3(indacene)_, 7-C*H*_3(indacene)_), 2.66 (s, 3H, OC*H*_3_), 2.94–3.00 (m, 2H, CC*H*_2_CH_2_CH_2_CH_2_N_3_), 3.43 (t, *J* = 6.7 Hz, 2H, CCH_2_CH_2_CH_2_C*H*_2_N_3_), 6.20 (s, 2H, 2-C*H*_(indacene)_, 6-C*H*_(indacene)_). ^13^C NMR (151 MHz, DMSO-*d*_6_): δ (ppm) = 14.1 (d, *J* = 2.2 Hz, 2C, 3-CH_3(indacene)_, 5-CH_3(indacene)_), 15.9 (2C, 1-CH_3(indacene)_, 7-CH_3(indacene)_), 27.2 (1C, C*C*H_2_CH_2_CH_2_CH_2_N_3_), 28.3–28.9 (m, 2C, CCH_2_*C*H_2_*C*H_2_CH_2_N_3_), 48.2 (d, *J* = 6.6 Hz, 1C, O*C*H_3_), 50.1 (1C, CCH_2_CH_2_CH_2_*C*H_2_N_3_), 121.5 (2C, C-2_(indacene)_, C-6_(indacene)_), 131.5 (2C, C-8a_(indacene)_, C-7a_(indacene)_), 139.7 (2C, C-1_(indacene)_, C-7_(indacene)_), 145.9 (1C, C-8_(indacene)_), 153.1 (2C, C-3_(indacene)_, C-5_(indacene)_). Exact mass (ESI): (*m*/*z*) = 326.1980 (calcd.326.1952 for C_18_H_25_BFN_5_O [M-OCH_3_]^+^). IR (neat): ṽ (cm^−1^) = 2953 (C-H, aliph), 2812 (C-H, aliph), 2083 (N=N=N), 1547 (C=C, arom), 1107 (C-O).

8-(4-Azidobutyl)-2,6-diethyl-4-fluoro-4-methoxy-1,3,5,7-tetramethyl-4-bora-3a,4a-diaza-s-indacene (**14b**) was prepared as follows:

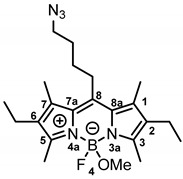



According to General Procedure A, bodipy dye **13b** (100 mg, 0.25 mmol, 1.0 eq.), TMSOTf (0.23 mL, 1.25 mmol, 5.0 eq.), methanol (1.0 mL, 24.9 mmol, 100 eq.) and DIPEA (0.43 mL, 2.49 mmol, 10 eq.) were reacted stepwise in CH_2_Cl_2_. The crude product was purified by automatic flash column chromatography; (cartridge: SNAP KP-Sil, 25 g (Biotage^®^), ethyl acetate:cyclohexane, gradient 5:95 → 18:82, 25 mL/min), R_f_ = 0.51 (ethyl acetate:cyclohexane = 1:4). Red solid, mp 98 °C, yield 69.6 mg (68%). Purity (HPLC, method 2): 79.2% (t_R_ = 20.1 min). C_22_H_33_BFN_5_O (M_r_ = 413.3). ^1^H NMR (400 MHz, DMSO-*d*_6_): δ (ppm) = 1.00 (t, *J* = 7.5 Hz, 6H, 2-CH_2_C*H*_3,_ 6-CH_2_C*H*_3(indacene)_), 1.56–1.67 (m, 2H, CCH_2_C*H*_2_CH_2_CH_2_N_3_), 1.68–1.79 (m, 2H, CCH_2_CH_2_C*H*_2_CH_2_N_3_), 2.34 (s, 6H, 1-C*H*_3(indacene)_, 7-C*H*_3(indacene)_), 2.35–2.43 (m, 10H, 2-C*H*_2_CH_3(indacene),_ 6-C*H*_2_CH_3(indacene),_ 3-C*H*_3(indacene)_, 5-C*H*_3(indacene)_), 2.63 (s, 3H, OCH_3_), 2.96–3.05 (m, 2H, CC*H*_2_CH_2_CH_2_CH_2_N_3_), 3.44 (t, *J* = 6.5 Hz, 2H, CCH_2_CH_2_CH_2_C*H*_2_N_3_). ^13^C NMR (101 MHz, DMSO-*d*_6_): δ (ppm) = 12.0 (d, *J* = 2.3 Hz, 2C, 3-CH_3(indacene)_, 5-CH_3(indacene)_), 12.9 (2C, 1-CH_3(indacene)_, 7-CH_3(indacene)_), 14.8 (2C, 2-CH_2_*C*H_3,_ 6-CH_2_*C*H_3_), 16.6 (2C, 2-*C*H_2_CH_3,_ 6-*C*H_2_CH_3_), 27.2 (1C, C*C*H_2_CH_2_CH_2_CH_2_N_3_), 28.6 (bs, 2C, CCH_2_*C*H_2_*C*H_2_CH_2_N_3_), 48.3 (bs, 1C, O*C*H_3_), 50.1 (1C, CCH_2_CH_2_CH_2_*C*H_2_N_3_), 130.9 0 (2C, C-8a_(indacene)_, C-7a _(indacene)_), 132.0 (2C, C-2_(indacene)_, C-6_(indacene)_), 134.9 (2C, C-1_(indacene)_, C-7 _(indacene)_), 144.5 (1C, C-8_(indacene)_), 151.5 (2C, C-3_(indacene)_, C-5_(indacene)_). Exact mass (ESI): (*m*/*z*) = 413.2698 (calcd. 413.2762 for C_22_H_33_BFN_5_O [M]^+^). IR (neat): ṽ (cm^−1^) = 2971 (C-H, aliph), 2803 (C-H, aliph), 2098 (N=N=N), 1544 (C=C, arom), 1111 (C-O).

2-[4-({1-[4-(4,4-Difluoro-1,3,5,7-tetramethyl-4-bora-3a,4a-diaza-s-indacen-8-yl)butyl]-1,2,3-triazol-4-yl}methoxy)phenyl]-2-(4-fluorophenyl)-2-phenylacetamide (**15a**) was prepared as follows:

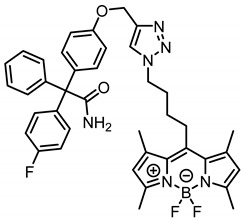



According to General Procedure B, azide **13a** (90.1 mg, 0.26 mmol, 1.0 eq.), propargyl ether **8** (93.7 mg, 0.26 mmol, 1.0 eq.), sodium ascorbate (310 mg, 1.57 mmol, 6.0 eq.) and CuSO_4_ (271 mg, 1.70 mmol, 6.5 eq.) were reacted in DMF and H_2_O. The crude product was purified by automatic flash column chromatography; Column 1: (cartridge: SNAP KP-Sil, 25 g (Biotage^®^), ethyl acetate:cyclohexane, gradient 70:30 → 90:10, 25 mL/min), Column 2: (cartridge: SNAP C18, 12 g (Biotage^®^), CH_3_CN:H_2_O, gradient 70:30 → 100:0, 12 mL/min), R_f_ = 0.40 (CH_2_Cl_2:_CH_3_OH = 95:5). Orange solid, mp 113 °C, yield 110 mg (60%). Purity (HPLC, method 2): 96.7% (t_R_ = 19.5 min). C_40_H_40_BF_3_N_6_O_2_ (M_r_ = 704.6). ^1^H NMR (599 MHz, CDCl_3_): δ (ppm) = 1.64–1.72 (m, 2H, CCH_2_C*H*_2_CH_2_CH_2_N), 2.15 (quint, *J* = 7.3 Hz, 2H, CCH_2_CH_2_C*H*_2_CH_2_N), 2.35 (s, 6H, 1-C*H*_3(indacene)_, 7-C*H*_3(indacene)_), 2.50 (s, 6H, 3-C*H*_3(indacene)_, 5-C*H*_3(indacene)_), 2.96–3.02 (m, 2H, CC*H*_2_CH_2_CH_2_CH_2_N), 4.39 (t, *J* = 7.1 Hz, 2H, CCH_2_CH_2_CH_2_C*H*_2_N), 5.19 (s, 2H, aryl-O-CH_2_), 5.74 (s, 1H, N*H*_2_), 5.77 (s, 1H, N*H*_2_), 6.04 (s, 2H, 2-C*H*_(indacene)_, 6-C*H*_(indacene)_), 6.88–6.95 (m, 2H, 3-*H*_(OPh)_, 5-*H*_(OPh)_), 6.95–7.01 (m, 2H, 3-*H*_(FPh)_, 5-*H*_(FPh)_), 7.14–7.20 (m, 2H, 2-*H*_(OPh)_, 6-*H*_(OPh)_), 7.23–7.25 (m, 2H, 2-H_(Ph)_, 6-H_(Ph)_), 7.27–7.33 (m, 5H, 2-H_(FPh)_, 6-H_(FPh)_, 3-H_(Ph)_, 4-H_(Ph)_, 5-H_(Ph)_), 7.58 (s, 1H, C*H*_(triazole)_). ^13^C NMR (151 MHz, CDCl_3_): δ (ppm) = 14.6 (s, 2C, 3-CH_3(indacene)_, 5-CH_3(indacene)_), 16.6 (2C, 1-CH_3(indacene)_, 7-CH_3(indacene)_), 27.7 (1C, C*C*H_2_CH_2_CH_2_CH_2_N), 28.8 (1C, CCH_2_*C*H_2_CH_2_CH_2_N), 30.5 (1C, CCH_2_CH_2_*C*H_2_CH_2_N), 50.2 (1C, CCH_2_CH_2_CH_2_*C*H_2_N), 62.2 (1C, aryl-O-*C*H_2_), 66.5 (1C, *C*CONH_2_), 114.3 (2C, C-3_(OPh)_, C-5_(OPh)_), 114.9 (d, *J* = 21.2 Hz, 2C, C-3_(FPh)_, C-5_(FPh)_), 122.0 (2C, C-2_(indacene)_, C-6_(indacene)_), 122.9 (1C, C-5_(triazole)_), 127.5 (1C, C-4_(Ph)_), 128.3 (2C, C-3_(Ph)_, C-5_(Ph)_), 130.3 (2C, C-2_(Ph)_, C-6_(Ph)_), 131.5 (2C, C-7a_(indacene)_, C-8a_(indacene)_), 131.7 (2C, C-2_(OPh)_, C-6_(OPh)_), 132.2 (d, *J* = 7.8 Hz, 2C, C-2_(FPh)_, C-6_(FPh)_), 135.9 (1C, C-1_(OPh)_), 139.3 (d, *J* = 3.5 Hz, 1C, C-1_(FPh)_), 140.3 (2C, C-1_(indacene)_, C-7_(indacene)_), 143.4 (1C, C-1_(Ph)_), 144.3 (1C, C-4_(triazole)_), 144.9 (1C, C-8_(indacene)_), 154.5 (2C, C-3_(indacene)_, C-5_(indacene)_), 157.4 (1C, C-4_(OPh)_), 161.8 (d, *J* = 247.3 Hz, 1C, C-4_(FPh)_), 175.8 (1C, CONH_2_). Exact mass (ESI): (*m*/*z*) = 685.3287 (calcd. 685.3268 for C_40_H_40_BF_3_N_6_O_2_ [M-F]^+^). IR (neat): ṽ (cm^−1^) = 3498 (CON-H), 2955 (C-H, aliph), 1678 (C=O), 1605 (C=C), 1547 (C=C, arom), 1227 (C-F), 1184 (C-O).

2-[4-({1-[4-(4-Fluoro-4-methoxy-1,3,5,7-tetramethyl-4-bora-3a,4a-diaza-s-indacen-8-yl)butyl]-1,2,3-triazol-4-yl}methoxy)phenyl]-2-(4-fluorophenyl)-2-phenylacetamide (**16a**) was prepared as follows:

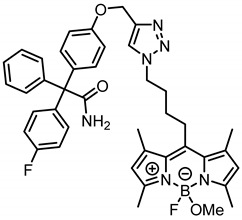



According to General Procedure A, bodipy dye **14a** (40.0 mg, 0.11 mmol, 1.0 eq.), propargyl ether **8** (40.2 mg, 0.11 mmol, 1.0 eq.), sodium ascorbate (119 mg, 0.66 mmol, 6.0 eq.) and CuSO_4_ (114 mg, 0.715 mmol, 6.5 eq.) were reacted in DMF and H_2_O. The crude product was purified by automatic flash column chromatography; Column 1: (cartridge: SNAP KP-Sil, 25 g (Biotage^®^), ethyl acetate:cyclohexane, gradient 70:30 → 90:10, 25 mL/min), Column 2: (cartridge: SNAP C18, 12 g (Biotage^®^), CH_3_CN:H_2_O, gradient 60:40 → 100:0, 12 mL/min), R_f_ = 0.34 (CH_2_Cl_2_:CH_3_OH = 95:5). Orange solid, mp 115 °C, yield 38.8 mg (49%). Purity (HPLC, Method 2): 95.2% (t_R_ = 18.1 min). C_41_H_43_BF_2_N_6_O_3_ (M_r_ = 716.6). ^1^H NMR (599 MHz, CDCl_3_): δ (ppm) = 1.65–1.74 (m, 2H, CCH_2_C*H*_2_CH_2_CH_2_N), 2.15 (quint, *J* = 7.4 Hz, 2H, CCH_2_CH_2_C*H*_2_CH_2_N), 2.36 (s, 6H, 1-C*H*_3(indacene)_, 7-C*H*_3(indacene)_), 2.50 (s, 6H, 3-C*H*_3(indacene)_, 5-C*H*_3(indacene)_), 2.84 (s, 3H, OC*H*_3_), 2.97–3.03 (m, 2H, CC*H*_2_CH_2_CH_2_CH_2_N), 4.40 (t, *J* = 7.1 Hz, 2H, CCH_2_CH_2_CH_2_C*H*_2_N), 5.19 (s, 2H, aryl-O-CH_2_), 5.74 (s, 1H, N*H*_2_), 5.77 (s, 1H, N*H*_2_), 6.04 (s, 2H, 2-C*H*_(indacene)_, 6-C*H*_(indacene)_), 6.90–6.95 (m, 2H, 3-*H*_(OPh)_, 5-*H*_(OPh)_), 6.95–7.01 (m, 2H, 3-*H*_(FPh)_, 5-*H*_(FPh)_), 7.15–7.20 (m, 2H, 2-*H*_(OPh)_, 6-*H*_(OPh)_), 7.22–7.25 (m, 2H, 2-H_(Ph)_, 6-H_(Ph)_), 7.27–7.34 (m, 5H, 2-H_(FPh)_, 6-H_(FPh)_, 3-H_(Ph)_, 4-H_(Ph)_, 5-H_(Ph)_), 7.59 (s, 1H, C*H*_(triazole)_). ^13^C NMR (151 MHz, CDCl_3_): δ (ppm) = 14.7 (d, *J* = 2.4 Hz, 2C, 3-CH_3(indacene)_, 5-CH_3(indacene)_), 16.6 (2C, 1-CH_3(indacene)_, 7-CH_3(indacene)_), 27.8 (1C, C*C*H_2_CH_2_CH_2_CH_2_N), 28.9 (1C, CCH_2_*C*H_2_CH_2_CH_2_N), 30.6 (1C, CCH_2_CH_2_*C*H_2_CH_2_N), 49.2 (d, *J* = 6.6 Hz, 1C, O*C*H_3_), 50.2 (1C, CCH_2_CH_2_CH_2_*C*H_2_N), 62.2 (1C, aryl-O-*C*H_2_), 66.5 (1C, *C*CONH_2_), 114.3 (2C, C-3_(OPh)_, C-5_(OPh)_), 114.9 (d, *J* = 21.3 Hz, 2C, C-3_(FPh)_, C-5_(FPh)_), 122.0 (2C, C-2_(indacene)_, C-6_(indacene)_), 122.9 (1C, C-5_(triazole)_), 127.5 (1C, C-4_(Ph)_), 128.3 (2C, C-3_(Ph)_, C-5_(Ph)_), 130.3 (2C, C-2_(Ph)_, C-6_(Ph)_), 131.7 (2C, C-2_(OPh)_, C-6_(OPh)_), 132.1–132.3 (m, 4C, C-7a_(indacene)_, C-8a_(indacene)_, C-2_(FPh)_, C-6_(FPh)_), 135.9 (1C, C-1_(OPh)_), 139.2–139.5 (m, 3C, 1C, C-1_(FPh)_, C-1_(indacene)_, C-7_(indacene)_), 143.4 (1C, C-1_(Ph)_), 144.3 (1C, C-4_(triazole)_), 144.6 (1C, C-8_(indacene)_), 154.6 (2C, C-3_(indacene)_, C-5_(indacene)_), 157.4 (1C, C-4_(OPh)_), 161.8 (d, *J* = 247.0 Hz, 1C, C-4_(FPh)_), 175.8 (1C, CONH_2_). Exact mass (ESI): (*m*/*z*) = 685.3322 (calcd. 685.3268 C_41_H_43_BF_2_N_6_O_3_ [M-OMe]^+^). IR (neat): ṽ (cm^−1^) = 3497 (CON-H), 2945 (C-H, aliph), 1678 (C=O), 1605 (C=C), 1547 (C=C, arom), 1300 (C-F), 1184 (C-O).

2-[4-({1-[4-(2,6-Diethyl-4-fluoro-4-methoxy-1,3,5,7-tetramethyl-4-bora-3a,4a-diaza-s-indacen-8-yl)butyl]-1,2,3-triazol-4-yl}methoxy)phenyl]-2-(4-fluorophenyl)-2-phenylacetamide (**16b**) was prepared as follows:

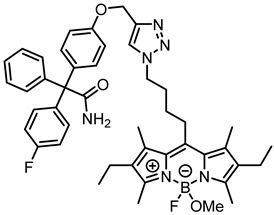



According to General Procedure B, bodipy dye **14b** (26.1 mg, 0.07 mmol, 1.0 eq.), propargyl ether **8** (28.9 mg, 0.07 mmol, 1.0 eq.), sodium ascorbate (86.3 mg, 0.44 mmol, 6.2 eq.) and CuSO_4_ (75.3 mg, 0.47 mmol, 6.7 eq.) were reacted in DMF and H_2_O. The crude product was purified by automatic flash column chromatography; Column 1: (cartridge: SNAP KP-Sil, 10 g (Biotage^®^), ethyl acetate:cyclohexane, gradient 50:50 → 80:20, 12 mL/min), R_f_ = 0.37 (CH_2_Cl_2_:CH_3_OH = 95:5). Red solid, mp 117 °C, yield 44.0 mg (81%). Purity (HPLC, Method 2): 97.3% (t_R_ = 20.4 min). C_45_H_51_BF_2_N_6_O_3_ (M_r_ = 772.7). ^1^H NMR (599 MHz, CDCl_3_): δ (ppm) = 1.03 (t, *J* = 7.6 Hz, 6H, 2-CH_2_C*H*_3,_ 6-CH_2_C*H*_3_), 1.70 (bs, 2H, CCH_2_C*H*_2_CH_2_CH_2_N), 2.16 (quint, *J* = 7.5 Hz, 2H, CCH_2_CH_2_C*H*_2_CH_2_N), 2.28 (s, 6H, 1-C*H*_3(indacene)_, 7-C*H*_3(indacene)_), 2.39 (q, *J* = 7.6 Hz, 4H, 2-C*H*_2_CH_3,_ 6-C*H*_2_CH_3_), 2.49 (s, 6H, 3-C*H*_3(indacene)_, 5-C*H*_3(indacene)_), 2.82 (s, 3H, OC*H*_3_), 3.02–3.08 (m, 2H, CC*H*_2_CH_2_CH_2_CH_2_N), 4.41 (bs, 2H, CCH_2_CH_2_CH_2_C*H*_2_N), 5.19 (s, 2H, aryl-O-CH_2_), 5.74 (s, 1H, N*H*_2_), 5.77 (s, 1H, N*H*_2_) 6.90–6.94 (m, 2H, 3-*H*_(OPh)_, 5-*H*_(OPh)_), 6.95–7.01 (m, 2H, 3-*H*_(FPh)_, 5-*H*_(FPh)_), 7.18 (d, *J* = 8.4 Hz, 2H, 2-*H*_(OPh)_, 6-*H*_(OPh)_), 7.23–7.25 (m, 2H, 2-H_(Ph)_, 6-H_(Ph)_), 7.27–7.33 (m, 5H, 2-*H*_(FPh)_, 6-*H*_(FPh)_, 3-*H*_(Ph)_, 4-*H*_(Ph)_, 5-H_(Ph)_), 7.59 (s, 1H, C*H*_(triazole)_). ^13^C NMR (151 MHz, CDCl_3_): δ (ppm) = 12.5 (d, *J* = 2.8 Hz, 2C, 3-CH_3(indacene)_, 5-CH_3(indacene)_), 13.6 (2C, 1-CH_3(indacene)_, 7-CH_3(indacene)_), 15.0 (2C, 2-CH_2_*C*H_3,_ 6-CH_2_*C*H_3_), 17.4 (2C, 2-*C*H_2_CH_3,_ 6-*C*H_2_CH_3_), 27.8 (1C, C*C*H_2_CH_2_CH_2_CH_2_N), 28.9 (1C, CCH_2_*C*H_2_CH_2_CH_2_N), 30.6 (1C, CCH_2_CH_2_*C*H_2_CH_2_N), 49.2 (d, *J* = 6.3 Hz, 1C, OCH_3_), 50.3 (1C, CCH_2_CH_2_CH_2_*C*H_2_N), 62.2 (1C, aryl-O-*C*H_2_), 66.5 (1C, *C*CONH_2_), 114.3 (2C, C-3_(OPh)_, C-5_(OPh)_), 114.9 (d, *J* = 21.3 Hz, 2C, C-3_(FPh)_, C-5_(FPh)_), 123.0 (1C, C-5_(triazole)_), 127.5 (1C, C-4_(Ph)_), 128.3 (2C, C-3_(Ph)_, C-5_(Ph)_), 130.3 (2C, C-2_(Ph)_, C-6_(Ph)_), 131.7 (4C, C-2_(OPh)_, C-6_(OPh)_, C-1_(indacene)_, C-7_(indacene)_), 132.2 (d, *J* = 7.9 Hz, 2C, C-2_(FPh)_, C-6_(FPh)_), 132.9 (2C, C-7a_(indacene)_, C-8a_(indacene)_), 134.6 (2C, C-2_(indacene)_, C-6_(indacene)_), 135.9 (1C, C-1_(OPh)_), 139.3 (d, *J* = 3.4 Hz, 1C, C-1_(FPh)_), 143.0 (1C, C-8_(indacene)_), 143.4 (1C, C-1_(Ph)_), 144.2 (1C, C-4_(triazole)_), 152.9 (2C, C-3_(indacene)_, C-5_(indacene)_), 157.4 (1C, C-4_(OPh)_), 161.8 (d, *J* = 247.1 Hz, 1C, C-4_(FPh)_), 175.8 (1C, CONH_2_). Exact mass (ESI): (*m*/*z*) = 741.3953 (calcd. 741.3900 for C_45_H_51_BF_2_N_6_O_3_ [M-OMe]^+^). IR (neat): ṽ (cm^−1^) = 3497 (CON-H), 2931 (C-H, aliph), 2859 (C-H, aliph), 1680 (C=O), 1586 (C=C), 1485 (C-H), 1253 (C-F), 1111 (C-O).

### 2.6. Synthesis of Fluorescein-Labeled Senicapoc Derivatives

2-{4-[2-(2-Azidoethoxy)ethoxy]phenyl}-2-(4-fluorophenyl)-2-phenylacetamide (**18a**) was prepared as follows:

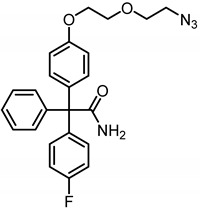



Phenol **17** (200 mg, 0.62 mmol, 1.0 eq.), 2-(2-azidoethoxy)ethyl tosylate TsOCH_2_CH_2_OCH_2_CH_2_N_3_, 178 mg, 0.62 mmol, 1 eq.) and Cs_2_CO_3_ (101 mg, 0.32 mmol, 0.5 eq.) were dissolved in DMF (8 mL). The mixture was stirred at 80 °C overnight. After cooling down to room temperature, the mixture was concentrated in vacuo. Ethyl acetate (10 mL) was added and the mixture was washed with LiCl solution (5% wt in H_2_O, 3 × 10 mL). The organic layer was dried (Na_2_SO_4_), filtered and concentrated in vacuo. The residue was used without further purification. R_f_ = 0.38 (cyclohexane:ethyl acetate = 1:1). Colorless oil, yield 190 mg (70%). Purity (HPLC, Method 1): 82.7% (t_R_ = 22.5 min). C_24_H_23_FN_4_O_3_ (M_r_ = 434.5). ^1^H NMR (600 MHz, CD_3_OD): δ (ppm) = 3.38 (t, *J* = 4.9 Hz, 2H, OCH_2_C*H*_2_N_3_), 3.71–3.74 (m, 2H, OC*H*_2_CH_2_N_3_), 3.83–3.87 (m, 2H, OCH_2_C*H*_2_O), 4.12–4.16 (m, 2H, OC*H*_2_CH_2_O), 6.87–6.91 (m, 2H, 3-*H*_(OPh)_, 5-*H*_(OPh)_), 6.97–7.04 (m, 2H, 3-*H*_(FPh)_, 5-*H*_(FPh)_), 7.13–7.18 (m, 2H, 2-*H*_(OPh)_, 6-*H*_(OPh)_), 7.25–7.31 (m, 7H, 2-*H*_(FPh)_, 6-*H*_(FPh)_, 2-*H*_(Ph)_, 3-*H*_(Ph)_, 4-*H*_(Ph)_, 5-*H*_(Ph)_, 6-*H*_(Ph)_). ^13^C NMR (151 MHz, CD_3_OD): δ (ppm) = 51.8 (1C, OCH_2_*C*H_2_N_3_), 68.7 (1C, *C*CONH_2_), 68.7 (1C, O*C*H_2_CH_2_O), 70.8 (1C, OCH_2_*C*H_2_O), 71.3 (1C, O*C*H_2_CH_2_N_3_), 115.0 (2C, C-3_(OPh)_, C-5_(OPh)_), 115.3 (d, *J* = 21.4 Hz, 2C, C-3_(FPh)_, C-5_(FPh)_), 128.1 (1C, C-4_(Ph)_), 129.0 (2C, C-3_(Ph)_, C-5_(Ph)_), 131.4 (2C, C-2_(Ph)_, C-6_(Ph)_), 132.7 (2C, C-2_(OPh)_, C-6_(OPh)_), 133.5 (d, *J* = 7.6 Hz, 2C, C-2_(FPh)_, C-6_(FPh)_), 136.9 (1C, C-1_(OPh)_), 141.2 (d, *J* = 3.4 Hz, 1C, C-1_(FPh)_), 145.0 (1C, C-1_(Ph)_), 146.5, 159.2 (1C, C-4_(OPh)_), 162.2 (d, *J* = 246.1 Hz, 1C, C-4_(FPh)_). A signal for the C-atom *C*ONH_2_ is not seen in the spectrum. Exact mass (APCI): (*m*/*z*) = 435.1769 (calcd. 435.1832 for C_24_H_23_FN_4_O_3_ [M + H^+^]^+^). IR (neat): ṽ (cm^−1^) = 3471(N-H), 2920 (C-H, aliph), 2866 (C-H, aliph), 2094 (N=N=N), 1678 (C=O), 1601 (C=C, arom), 1504 (C=C, arom).

*tert*-Butyl N-(2-{2-[2-(2-{4-[α-carbamoyl-α-(4-fluorophenyl)benzyl]phenoxy} ethoxy)ethoxy]ethoxy}ethyl)carbamate (**19b**) was prepared as follows:

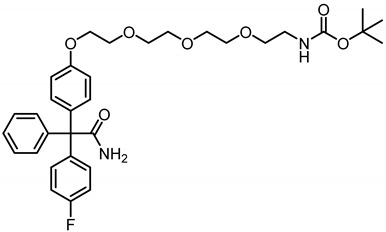



Phenol **17** (50 mg, 0.16 mmol, 1.0 eq.), tosylate TsO(CH_2_CH_2_O)_3_CH_2_CH_2_NHBoc, (69.6 mg, 0.16 mmol, 1.0 eq.) and Cs_2_CO_3_ (23.4 mg, 0.08 mmol, 0.5 eq.) were dissolved in DMF (5 mL) and the mixture was heated to reflux overnight. The mixture was allowed to cool down to room temperature and the solvent was removed under reduced pressure. Ethyl acetate (10 mL) was added to the residue and the mixture was washed with LiCl solution (5% wt in H_2_O, 3 × 10 mL). The organic layer was dried (Na_2_SO_4_), filtered and concentrated in vacuo. The residue was used without further purification. R_f_ = 0.14 (cyclohexane:ethyl acetate = 33:67). Colorless oil, yield 93.4 mg (100%). Purity (HPLC, method 1): 66.3% (t_R_ = 22.3 min). C_33_H_41_FN_2_O_7_ (M_r_ = 596.7). ^1^H NMR (600 MHz, CD_3_OD): δ (ppm) = 1.42 (s, 9H, 3 x C*H*_3_), 3.19–3.21 (m, 2H, CH_2_C*H*_2_NHBoc), 3.48–3.50 (m, 2H, C*H*_2_CH_2_NHBoc), 3.58–3.59 (m, 2H, OC*H*_2_), 3.62–3.64 (m, 2H, OCH_2_), 3.65–3.66 (m, 2H, OCH_2_), 3.68–3.72 (m, 2H, OCH_2_), 3.82–3.85 (m, 2H, PhOCH_2_C*H*_2_), 4.12–4.15 (m, 2H, PhOC*H*_2_CH*_2_*), 6.89 (d, *J* = 8.9 Hz, 2H, 3-*H*_(OPh)_, 5-*H*_(OPh)_), 6.98–7.04 (m, 2H, 3-*H*_(FPh)_, 5-*H*_(FPh)_), 7.16 (d, *J* = 9.0 Hz, 2H, 2-*H*_(OPh)_, 6-*H*_(OPh)_), 7.24–7.32 (m, 7H, 2-*H*_(FPh)_, 6-*H*_(FPh)_, 2-*H*_(Ph)_, 3-*H*_(Ph)_, 4-*H*_(Ph)_, 5-*H*_(Ph)_, 6-*H*_(Ph)_). ^13^C NMR (151 MHz, CD_3_OD): δ (ppm) = 28.8 (3C, 3 x *C*H_3_), 41.3 (1C, OCH_2_*C*H_2_NHBoc), 68.6 (1C, PhO*C*H_2_CH_2_), 69.8 (1C, *C*CONH_2_), 70.8 (1C, PhOCH_2_*C*H_2_), 71.0 (1C, O*C*HCH_2_NHBoc), 71.3 (1C, O*C*H_2_), 71.6 (1C, O*C*H_2_), 71.6 (1C, O*C*H_2_), 71.7 (1C, O*C*H_2_), 80.1 (1C, *C*(CH_3_)_3_), 115.0 (2C, C-3_(OPh)_, C-5_(OPh)_), 115.3 (d, *J* = 21.4 Hz, 2C, C-3_(FPh)_, C-5_(FPh)_), 128.1 (1C, C-4_(Ph)_), 129.0 (2C, C-3_(Ph)_, C-5_(Ph)_), 131.4 (2C, C-2_(Ph)_, C-6_(Ph)_), 132.7 (2C, C-2_(OPh)_, C-6_(OPh)_), 133.5 (d, *J* = 7.7 Hz, 2C, C-2_(FPh)_, C-6_(FPh)_), 136.9 (1C, C-1_(OPh)_), 141.2 (d, *J* = 3.5 Hz, 1C, C-1_(FPh)_), 145.0 (1C, C-1_(Ph)_), 158.4 (1C, C=O_Boc_), 159.2 (1C, C-4_(OPh)_), 163.0 (d, *J* = 245.6 Hz, 1C, C-4_(FPh)_), 178.5 (1C, *C*ONH_2_). Exact mass (APCI): (*m*/*z*) = 497.2488 (calcd. 497.2452 for C_33_H_41_FN_2_O_7_ [M-C_5_H_10_O_2_/+H^+^]^+^). IR (neat): ṽ (cm^−1^) = 3478 (N-H), 2937 (C-H, aliph), 2870 (C-H, aliph), 1678 (C=O), 1607 (C=C, arom), 1504 (C=C, arom).

2-{4-[2-(2-Aminoethoxy)ethoxy]phenyl}-2-(4-fluorophenyl)-2-phenylacetamide (**20a**) was prepared as follows:

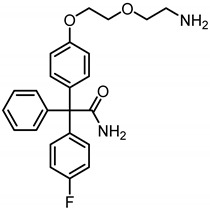



Azide **18a** (150 mg, 0.35 mmol, 1.0 eq) and 10% Pd/C (3.5 mg) were added to ethyl acetate (5 mL) and the mixture was stirred under H_2_-atmosphere (5 bar) for 1 h. The suspension was filtered over Celite^®^ and the solution was concentrated in vacuo. The primary amine **20a** was used without further purification. Colorless oil, yield 140 mg (98%). C_24_H_25_FN_2_O_3_ (M_r_ = 408.5). Exact mass (APCI): (*m*/*z*) = 409.1902 (calcd. 409.1928 for C_24_H_25_FN_2_O_3_ [M + H^+^]^+^).

2-[4-(2-{2-[3-(3′,6′-Dihydroxy-3-oxo-3*H*-spiro[[2]benzofuran-1,9′-xanthen]-5-yl)thioureido]ethoxy}ethoxy)phenyl]-2-(4-fluorophenyl)-2-phenylacetamide (**21a**) was prepared as follows:

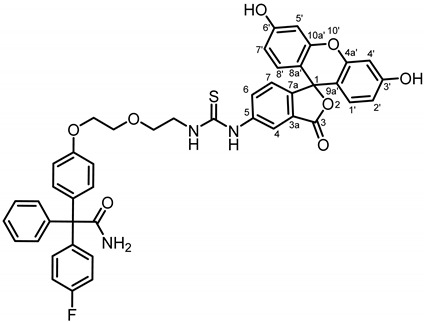



Primary amine **20a** (100 mg, 0.24 mmol, 1.0 eq.) and fluorescein isothiocyanate (FITC, 114 mg, 0.29 mmol, 1.2 eq.) were dissolved in CH_2_Cl_2_ (5 mL). Et_3_N (0.04 mL, 0.29 mmol, 1.2 eq.) was added and the mixture was stirred overnight at room temperature. The mixture was concentrated in vacuo and the residue was purified by automatic flash column chromatography (cartridge: SNAP C18, 12 g (Biotage^®^), H_2_O:CH_3_CN, gradient 90:10 → 20:80, 12 mL/min), R_f_ = 0.68 (CH_3_OH:CH_2_Cl_2_ = 10:90). Orange solid, mp 168 °C, yield 83.0 mg (43%). Purity (HPLC, Method 1): 96.3% (t_R_ = 20.9 min). C_45_H_36_FN_3_O_8_S (M_r_ = 797.9). ^1^H NMR (600 MHz, CD_3_OD): δ (ppm) = 3.75–3.79 (m, 2H, OC*H*_2_CH_2_N), 3.82–3.89 (m, 4H, OCH_2_C*H*_2_OCH_2_C*H*_2_N), 4.15 (t, *J* = 4.6 Hz, 2H, OC*H*_2_CH_2_O), 6.57 (dd, *J* = 8.9/2.3 Hz, 2H, 2′-H_(FITC)_, 7′-H_(FITC)_), 6.67 (d, *J* = 2.3 Hz, 2H, 4′-H_(FITC)_, 5′-H_(FITC)_), 6.84–6.87 (m, 2H, 3-*H*_(OPh)_, 5-*H*_(OPh)_), 6.89 (d, *J* = 8.9 Hz, 2H, 1′-H_(FITC)_, 8′-H_(FITC)_), 6.96 (t, *J* = 8.6 Hz, 2H, 3-*H*_(FPh)_, 5-*H*_(FPh)_), 7.09 (s, 1H, 7-H_(FITC)_), 7.10–7.12 (m, 2H, 2-*H*_(OPh)_, 6-*H*_(OPh)_), 7.19–7.26 (m, 7H, 2-*H*_(FPh)_, 6-*H*_(FPh)_, 2-*H*_(Ph)_, 3-*H*_(Ph)_, 4-*H*_(Ph)_, 5-*H*_(Ph)_, 6-*H*_(Ph)_), 7.70 (dd, *J* = 8.2/2.1 Hz, 1H, 6-H_(FITC)_), 8.03 (s, 1H, 4-H_(FITC)_). Signals for OH and NH protons are not seen in the spectrum. ^13^C NMR (151 MHz, CD_3_OD): δ (ppm) = 45.6 (1C, OCH_2_*C*H_2_N), 67.8 (1C, *C*CONH_2_), 68.7 (1C, O*C*H_2_CH_2_O), 70.7 (2C, *C*H_2_O*C*H_2_),103.7 (2C, C-4′_(FITC)_, C-5′_(FITC)_), 115.1 (2C, C-3_(OPh)_, C-5_(OPh)_), 115.3 (d, *J* = 21.4 Hz, 2C, C-3_(FPh)_, C-5_(FPh)_), 116.5 (2C, C-2′_(FITC)_, C-7′_(FITC)_), 121.9 (1C, C-4_(FITC)_), 128.0 (1C, C-4_(Ph)_), 128.1 (1C, C-7_(FITC)_), 128.8 (1C, C-6_(FITC)_), 129.0 (2C, C-3_(Ph)_, C-5_(Ph)_), 131.3–131.4 (m, 4C, C-1′_(FITC)_, C-8′_(FITC)_, C-2_(Ph)_, C-6_(Ph)_), 132.7 (2C, C-2_(OPh)_, C-6_(OPh)_), 133.4 (d, *J* = 7.8 Hz, 2C, C-2_(FPh)_, C-6_(FPh)_), 136.9 (1C, C-1_(OPh)_), 141.1 (1C, C-1_(FPh)_), 145.0 (1C, C-1_(Ph)_), 156.4 (2C, C-3′_(FITC)_, C-6′_(FITC)_), 159.1 (1C, C-4_(OPh)_), 162.9 (d, *J* = 245.3 Hz, 1C, C-4_(FPh)_), 171.9 (1C, C-3_(FITC)_), 178.4 (1C, *C*ONH_2_). Due to the low intensity, signals for C-1_(FITC)_, C-3a_(FITC)_, C-5a_(FITC)_, C-7a_(FITC)_, C-4a′_(FITC)_, C-8a′_(FITC)_, C-9a′_(FITC)_, C-10a′_(FITC)_ and S=C could not be identified. Exact mass (ESI): (*m*/*z*) = 798.2362 (calcd. 798.2285 for C_45_H_36_FN_3_O_8_S [M + H^+^]^+^). IR (neat): ṽ (cm^−1^) = 3400–3100 (O-H, N-H), 3050 (C-H arom), 2927 (C-H, aliph), 2120 (S=C), 1751 (C=O), 1581 (C=C, arom), 1501 (C=C, arom).

2-(4-{12-[3-(3′,6′-Dihydroxy-3-oxo-3*H*-spiro[[2]benzofuran-1,9′-xanthen]-5-yl)thioureido]-1,4,7,10-tteraoxadodecan-1-yl}phenyl)-2-(4-fluorophenyl)-2-phenylacetamide (**21b**) was prepared as follows:

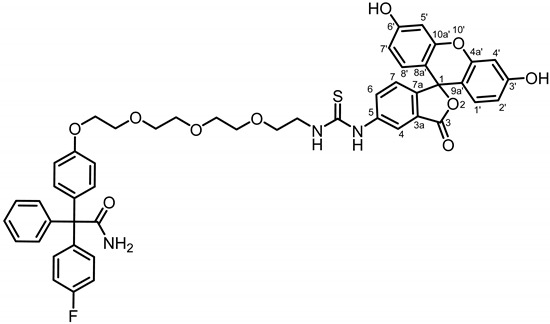



Boc derivative **19b** (84.0 mg, 0.14 mmol, 1.0 eq.) was dissolved in CH_2_Cl_2_ (10 mL). After addition of TFA (2 drops), rising gas bubbles were observed. The mixture was stirred overnight at room temperature. The solvent was removed under reduced pressure yielding primary amine **20b** as yellow oil. The crude product (**20b**, 50 mg) and fluorescein isothiocyanate (FITC, 54.9 mg, 0.14 mmol, 1.4 eq.) were dissolved in CH_2_Cl_2_. Et_3_N (0.02 mL, 0.14 mmol, 1.4 eq.) was added and the mixture was stirred overnight at room temperature. The mixture was concentrated in vacuo and *t*he residue was purified by automatic flash column chromatography; Column 1 (cartridge: SNAP C18, 12 g (Biotage^®^), H_2_O:CH_3_CN, gradient 90:10 → 0:100, 12 mL/min), Column 2 (cartridge: SNAP C18, 12 g (Biotage^®^), H_2_O:CH_3_CN, gradient 90:10 → 20:80, 12 mL/min), R_f_ = 0.68 (CH_3_OH: CH_2_Cl_2_ = 10:90). Orange solid, mp 150 °C, yield 49.2 mg (40%). Purity (HPLC, Method 1): 63.4% (t_R_ = 21.0 min). C_49_H_44_FN_3_O_10_S (M_r_ = 886.0). ^1^H NMR (600 MHz, DMSO-*d*_6_): δ (ppm) = 3.63–3.67 (m, 8H, 4 × OCH_2_), 3.69 (t, *J* = 5.2 Hz, 2H, OC*H*_2_CH_2_NH), 3.79 (t, *J* = 4.7 Hz, 2H, PhOCH_2_C*H*_2_O), 3.80–3.86 (m, 2H, OCH_2_C*H*_2_NH), 4.06 (t, *J* = 4.7 Hz, 2H, PhOC*H*_2_CH_2_O), 6.56 (dd, *J* = 8.9/2.3 Hz, 2H, 2′-H_(FITC)_, 7′-H_(FITC)_), 6.66 (d, *J* = 2.4 Hz, 2H, 4′-H_(FITC)_, 5′-H_(FITC)_), 6.82 (d, *J* = 8.9 Hz, 2H, 3-*H*_(OPh)_, 5-*H*_(OPh)_), 6.85 (d, *J* = 9.0 Hz, 2H, 1′-H_(FITC)_, 8′-H_(FITC)_), 6.98 (t, *J* = 8.8 Hz, 2H, 3-*H*_(FPh)_, 5-*H*_(FPh)_), 7.10–7.14 (m, 3H, 7-H_(FITC)_, 2-*H*_(OPh)_, 6-*H*_(OPh)_), 7.22–7.28 (m, 7H, 2-*H*_(FPh)_, 6-*H*_(FPh)_, 2-*H*_(Ph)_, 3-*H*_(Ph)_, 4-*H*_(Ph)_, 5-*H*_(Ph)_, 6-*H*_(Ph)_), 7.76 (dd, *J* = 8.6/2.3 Hz, 1H, 6-H_(FITC)_), 8.07 (s, 1H, 4-H_(FITC)_). Signals for OH and NH protons are not seen in the spectrum. ^13^C NMR (151 MHz, DMSO-*d*_6_): δ (ppm) = 45.5 (1C, OCH_2_*C*H_2_N), 67.8 (1C, *C*CONH_2_), 68.6 (1C, PhO*C*H_2_CH_2_O), 69.8 (1C, O*C*H_2_CH_2_N), 70.8 (1C, PhOCH_2_*C*H_2_N), 71.6 (4C, 4 × O*C*H_2_), 103.7 (2C, C-4′_(FITC)_, C-5′_(FITC)_), 115.0 (2C, C-3_(OPh)_, C-5_(OPh)_), 115.3 (d, *J* = 21.3 Hz, 2C, C-3_(FPh)_, C-5_(FPh)_), 116.3 (2C, C-2′_(FITC)_, C-7′_(FITC)_), 121.4 (1C, C-4_(FITC)_), 128.0 (1C, C-4_(Ph)_), 128.1 (1C, C-7_(FITC)_), 129.0 (2C, C-3_(Ph)_, C-5_(Ph)_), 129.0 (1C, C-6_(FITC)_), 131.1 (2C,C-1′_(FITC)_, C-8′_(FITC)_), 131.4 (2C, C-2_(Ph)_, C-6_(Ph)_), 132.7 (2C, C-2_(OPh)_, C-6_(OPh)_), 133.5 (d, *J* = 7.9 Hz, 2C, C-2_(FPh)_, C-6_(FPh)_), 136.8 (1C, C-1_(OPh)_), 141.1 (d, *J* = 3.4 Hz, 1C, C-1_(FPh)_), 145.0 (1C, C-1_(Ph)_), 159.1 (1C, C-4_(OPh)_), 162.9 (d, *J* = 245.8 Hz, 1C, C-4_(FPh)_), 171.8 (1C, C-3_(FITC)_), 178.5 (1C, *C*ONH_2_). Due to the low intensity, signals for C-1_(FITC)_, C-3a_(FITC)_, C-5a_(FITC)_, C-7a_(FITC)_, C-3′_(FITC)_, C-4a′_(FITC)_, C-6′_(FITC)_, C-8a′_(FITC)_, C-9a′_(FITC)_, C-10a′_(FITC)_ and S=C could not be identified. Exact mass (APCI): (*m*/*z*) = 886.2892 (calcd. 886.2809 for C_49_H_44_FN_3_O_10_S [M + H^+^]^+^). IR (neat): ṽ (cm^−1^) = 3400–3200 (O-H), 2933 (C-H, aliph), 2864 (C-H, aliph), 2120 (S=C), 1751 (C=O, lactone), 1663 (C=O), 1582 (C=C, arom), 1501 (C=C, arom).

### 2.7. Determination of the logD_7.4._ Value

The logD_7.4_ values were recorded using the micro shake flask method [31,32]. In this method, the test compound is distributed between *n*-octanol and MOPS buffer pH 7.4, and the amount of the compound in the buffer layer is determined by mass spectrometry.

### 2.8. Determination of the logP Value

The logP values were recorded using HPLC analysis [33]. Thus, the retention time of the test compound in a standardized reversed phase HPLC system is correlated with the retention times of compounds with known logP values.

### 2.9. UV–Vis Absorption and Time-Resolved Photoluminescence Spectroscopy

All solvents used were of spectrometric grade (Uvasol^®^). UV-visible absorption spectra were measured using a Shimadzu UV–VIS spectrophotometer (UV-3600i). Photoluminescence quantum yields were measured with a Hamamatsu Photonics absolute PL quantum yield measurement system (C9920-02) equipped with a L9799-01 CW Xe light source (150 W), a monochromator, a C7473 photonic multi-channel analyzer, an integrating sphere and employing U6039-05 software, version 3.6.0. (Hamamatsu Photonics, Ltd., Shizuoka, Japan). Steady-state excitation and emission spectra were recorded on a FluoTime 300 spectrometer from PicoQuant equipped with a 300 W ozone-free Xe lamp (200–1100 nm), a 10 W Xe-flash lamp (200–1100 nm, pulse width ca. 1 µs) with repetition rates of 1–300 Hz, a double-grating excitation monochromator (Czerny–Turner type, grating with 1200 lines/mm, blaze wavelength: 300 nm), diode lasers (pulse width < 20 ps) operated by a computer-controlled laser driver PDL-828 “Sepia II” (repetition rate up to 80 MHz, burst mode for slow and weak decays), two emission monochromators (Czerny–Turner, selectable between double-grating blazed at 500 nm with 2.7 nm/mm dispersion and 1200 lines/mm, or single-grating blazed at 1250 nm with 5.4 nm/mm dispersion and 600 lines/mm) with adjustable slit width between 25 μm and 7 mm. Glam–Thompson polarizers for excitation (after the Xe-lamps) and emission (after the sample) were used. Different sample holders (Peltier-cooled mounting unit ranging from −15 to 110 °C or an adjustable front-face sample holder), along with two detectors (namely a PMA Hybrid-07 from PicoQuant with transit time spread FWHM < 50 ps, 200–850 nm, or a H10330C-45-C3 NIR detector with transit time spread FWHM 0.4 ns, 950–1400 nm from Hamamatsu) were used. Steady-state spectra and photoluminescence lifetimes were recorded in TCSPC mode by a PicoHarp 300 (minimum base resolution 4 ps) or in MCS mode by a TimeHarp 260 (where up to several ms can be traced). Emission and excitation spectra were corrected for source intensity (lamp and grating) by standard correction curves. For samples with lifetimes in the ns order, an instrument response function calibration (IRF) was performed using a diluted Ludox^®^ dispersion. Lifetime analysis was performed using the commercial EasyTau 2 software, version 2.3. (PicoQuant, Berlin, Germany). The quality of the fit was assessed by minimizing the reduced chi squared function (χ^2^) and visual inspection of the weighted residuals and their autocorrelation.

### 2.10. Cell Staining Experiments

#### 2.10.1. Reagents for Cell Staining Experiments

*Stock solutions:* Stock solutions of the fluorescently labeled senicapoc derivatives **9a**, **9b**, **10a**, **10b**, **15a**, **16a**, **16b**, **21a**, and **21b** and corresponding azide precursors were prepared with a concentration of 10 mM in DMSO. Stock solutions of senicapoc (**3**) with concentrations of 30 mM and 60 mM in DMSO were prepared as well.

Staining/blocking solutions: For the staining solutions, the stock solutions of the senicapoc derivatives **9a**, **9b**, **10a**, **10b**, **15a**, **16a**, **16b**, **21a**, and **21b** and corresponding azide precursors were diluted with either PBS 1:1000 for a 10 µM solution or 2:1000 for a 20 µM solution. The senicapoc stock solutions were diluted with PBS in a 1:1000 ratio to generate 30 µM and 60 µM senicapoc blocking solutions. All staining/blocking solutions were prepared in Eppendorf^®^ tubes and were vortexed for 1 min by using a Vortex (Scientific^®^).

Permeabilization solution: For the permeabilization solution, 0.1% saponin was dissolved in PBS supplemented with 10% FCS (fetal calf serum, FCS Superior).

Primary antibody staining solution: A rabbit anti-K_Ca_3.1 antibody (#AV35098, Sigma Aldrich, St. Louis, MO, USA) was diluted in a ratio of 1:300 or 1:500 in the permeabilization solution.

Secondary antibody staining solution: A goat anti-rabbit antibody labeled with Cyanin 3 (Cy3) was diluted in a ratio of 1:500 or 1:1000 in the permeabilization solution.

#### 2.10.2. Cell Culture

The cells were cultured and prepared in cooperation with the Instiute of Physiology II by Ilka Neumann.

The A549-3R and HEK293 cell lines were cultured in DMEM-high glucose (Dulbecco’s modified Eagle’s medium—high glucose, Sigma Aldrich) supplemented with 10% FBS (fetal bovine serum, FBS Superior, Biochrom GmbH, Berlin, Germany) and incubated at 37 °C and 5% CO_2_.

For the experiments, glass bottom dishes were covered with 250 µL of 0.1% poly-L-lysine and incubated for 30–45 min at room temprature. In the meantime, the cell lines (A549-3R or HEK293) were trypsinized and counted. Subsequently, after removing the poly-L-lysin and washing the glass bottom dish with 1 mL of PBS, 15,000 cells in 2 mL DMEM-high glucose and 10% FBS were added to the glass bottom dish. The cells were incubated for 24 h at 37 °C and 5% CO_2_. After incubation, the cells were used for the next procedures.

#### 2.10.3. Fixation of the Cells

For the fixation of the cells, the DMEM medium was removed from the glass bottom dish after incubation for 24 h, and the cells were washed with 1 mL of PBS. Subsequently, 1 mL of 4% paraformaldehyde in PBS solution was added to the glass bottom dish and the mixture was incubated for 30 min at room temperature. Thereafter, the cells were washed five times with 1 mL of PBS and used for the cell staining experiments.

#### 2.10.4. Cell Staining Protocols

Protocol 1: standard staining protocol

After fixation of the cells (Section 2.10.2), 300 µL of the 10 µM staining solution was added to the glass bottom dish. The cells were incubated for 10 min at 4 °C in a dark chamber. After incubation, the cells were washed five times with 1 mL of PBS and used subsequently for fluorescence microscopy.

Protocol 2: staining of living cells

The cells were used without fixation. After incubation of the cells for 24 h (Section 2.10.1), the medium was removed, and the cells were washed three times with 1 mL of PBS. Subsequently, the cells were stained as described in Protocol 1.

Protocol 3: blocking with senicapoc using fixed cells

After fixation of the cells (Section 2.10.2), 300 µL of a 30 µM solution of senicapoc blocking solution was added to the glass bottom dish. The glass bottom dish was incubated for 5 min at 4 °C. After incubation, the solution was removed and a mixture of 150 µL of 60 µM senicapoc solution and 150 µL of 20 µM staining solution were added to the glass bottom dish. The cells were incubated for 10 min at 4 °C in a dark chamber. Subsequently, the cells were washed five times with PBS and then used for fluorescence microscopy.

Protocol 4: blocking with senicapoc using living cells

The cells were used without fixation. After incubating the cells for 24 h (Section 2.10.1), the medium was removed, and the cells were washed three times with 1 mL of PBS. Subsequently, the cells were stained as described in Protocol 3.

Protocol 5: staining after permeabilization

After fixation of the cells (Section 2.10.2), 1 mL of permeabilization solution was added to the glass bottom dish. The cells were incubated for 30 min at 4 °C and afterwards washed three times with PBS. Subsequently, Protocol 1 was performed.

Protocol 6: staining with antibodies

After fixation of the cells (Section 2.10.2), the cells were permeabilized with 1 mL of permeabilization solution for 30 min. The solution was removed from the cells and 200 µL of primary antibody staining solution was added. The cells were incubated for 2 h at 4 °C in a dark chamber. After incubation, the cells were washed three times with PBS and 200 µL of the secondary antibody staining solution was added to the glass bottom dish. The cells were incubated for 30 min at 4 °C in a dark chamber and followed by three-time washing with 1 mL of PBS. Subsequently, the cells were used for fluorescence microscopy with the TRITC filter set.

Protocol 7: co-staining with fluorescently labeled ligands and antibodies

Protocol 7a: Labeling with the antibody first and subsequently with the fluorescently labeled senicapoc derivative

The cells were stained with antibodies as described in Protocol 6. Afterwards, the cells were stained with 300 µL of staining solution and the mixture was incubated for 10 min at 4 °C in a dark chamber. Subsequently, the cells were washed five times with 1 mL of PBS and were used for fluorescence microscopy.

Protocol 7b: labeling with fluorescently labeled senicapoc derivative first and second with the antibody

The cells were treated as described in Protocol 5 to permeabilize the cells. Subsequently, staining solution was added and the cells were incubated for 10 min as described in Protocol 1. After washing the cells three times with PBS, Protocol 6 for the antibody staining was performed. Subsequently, the cells were used for fluorescence microscopy.

#### 2.10.5. Fluorescence Microscopy

An Axiovert 200 inverted microscope (Carl Zeiss AG, Jena, Germany) with a ×100 oil immersion objective and a digital camera (SPOT RT SE from Diagnostic Instruments Inc., Livingston, UK) were used. The camera was controlled by MetaVue software (version 6.3r6, Visitron, Germany). Two different filter sets were employed, the FITC filter set (Carl Zeiss Filter Set 10 bandwidth excitation filter 450–490, color splitter 510, bandpass filter 515–565) and the TRITC filter set (Carl Zeiss Filter Set 15, bandpass excitation filter 546/12, dichroic mirror 580, emission filter Long Pass 590).

Using the free imaging software ImageJ (1.53f51, NIH, USA), squares with an edge length of 50 × 50 pixels, corresponding to an area of 9 µm^2^, were randomly placed in a cell and signals with Full Width at Half Maximum (FWHM) of ≤5 pixels (~300 nm) were counted as a K_Ca_3.1 channel. For the evaluation, 7 squares were analyzed.

For all experiments, at least three biological replicates (N = 3) were performed, and for each replicate 3 cells (n = 3) were analyzed.

### 2.11. Patch Clamp Experiments

#### 2.11.1. Cell Culture

A549-3R cells were used for the experiments. The cells were cultured in DMEM with 4.5 g/L glucose and supplemented with 10% FCS at 37 °C and 5% CO_2_ in cell culture dishes (Ø = 10 cm).

#### 2.11.2. Reagents for Patch Clamp Experiments

Bath solution: 140 mM NaCl, 5 mM KCl, 10 mM HEPES, 1 mM MgCl_2_ and 1 mM CaCl_2_ in an aqueous solution; pH = 7.4 with NaOH.

Pipette solution: 140 mM KCl, 10 mM HEPES, 1.3 mM EGTA, 1 mM MgCl_2_ and 1.217 mM CaCl_2_ in an aqueous solution; pH = 7.4 with NaOH; free Ca^2+^ concentration of 1 µM.

1-EBIO solution: 100 mM stock solution of 1-ethylbenzimidazolone (1-EBIO) in DMSO was diluted with the bath solution to 50 µM.

Test solution: A 10 mM stock solution of imaging compound **10b** in DMSO was diluted with the bath solution to 1 µM.

Senicapoc solution: 10 mM stock solution of senicapoc (**3**) in DMSO was diluted with bath solution to 1 µM.

The final concentration of DMSO in the application solutions was 0.15%.

#### 2.11.3. Whole-Cell Patch Clamp Experiment

Patch clamp experiments were performed in cooperation with the Institute of Physiology I by Elke Naß and Dr. Laura Vinnenberg.

Whole-cell patch clamp experiments were conducted at room temperature using borosilicate glass pipettes (GC150TF-10, Harvard Ltd., Boston, MA, USA) connected to an EPC-10 amplifier (HEKA Electronics, Lambrecht, Germany). The typical electrode resistance was 3–4 MOhm, while the series resistance was in the range of 5–15 MOhm. Series resistance compensation of >30% was routinely used. Voltage-clamp experiments on cultured cells were controlled by PatchMaster software (HEKA Electronics, Germany, https://patchmaster.com/ accessed on 10 January 2025). Current density was calculated by dividing the current amplitude determined at the end of the depolarization voltage ramp to +60 mV by the membrane capacitance obtained from the slow capacitance compensation. The calculated free Ca^2+^ concentration of the pipette solution was 1 µmol/L to obtain full activation of the K_Ca_ channels during the patch clamp experiments. The test solutions were applied for a time period of 1–2 min. A multi-barreled application pipette with a tip diameter of ~100 µm was used for test solution applications close to the recorded cell. Recordings were analyzed using FitMaster and statistical analysis was calculated by Origin (OriginPro 2023b).

## 3. Results and Discussion

### 3.1. Synthesis of Fluorescently Labeled Senicapoc Derivatives

**Scheme 1:**
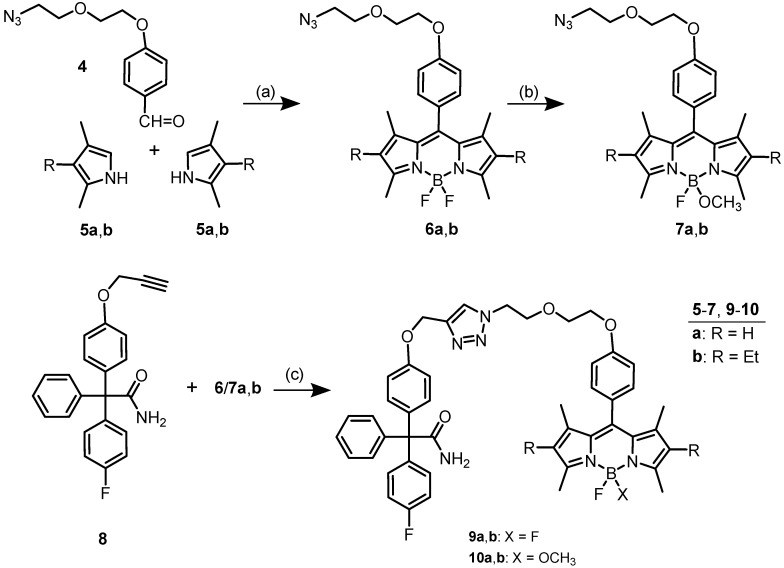
Synthesis of senicapoc bodipy conjugates containing a phenylene moiety in the linker. Reagents and reaction conditions: (a) 1. F_3_CCO_2_H, CH_2_Cl_2_, rt, 24 h; 2. DDQ, CH_2_Cl_2_, rt, 30 min; 3. BF_3_^.^OEt_2_, NEt_3_, rt, 16 h, yield over three steps: 13% (**6a**), 17% (**6b**). (**b**) TMSOTf, CH_2_Cl_2_, 0 °C, 2.5 min; then CH_3_OH, DIPEA, rt, 20 min, 39% (**7a**), 46% (**7b**). (c) CuSO_4_, Na-ascorbate, DMF, H_2_O, rt, 16 h, 43% (**9a**), 18% (**9b**), 25% (**10a**), 30% (**10b**). Compounds of the **a**-series have already been reported [27].

In our first approach [26], fluorescently labeled probes of type **9** (see Scheme 1) with one or four oxyethylene moieties between the phenyl and 1,2,3-triazolyl moieties were described. The dimethylpyrrole (**5a**)-based probe with one oxyethylene moiety showed clear staining of single K_Ca_3.1 channels. A longer linker (four oxyethylene moieties) exhibited reduced interactions with the binding site within the ion channel and the corresponding ethyl-dimethylpyrrole (**5b**) derivatives led to unspecific labeling due to increased lipophilicity. However, bodipy dyes derived from ethyldimethylpyrrole **5b** displayed higher photoluminescence quantum yields and longer fluorescence life times compared to dimethylpyrrole (**5a**)-derived bodipy dyes. Therefore, ethyl-substituted senicapoc derivatives **9b** and **10b** with two oxyethylene moieties between the bodipy phenyl ring and the central 1,2,3-triazolyl ring were envisaged. The additional polar oxyethylene moiety in the linker should not reduce the interaction with the binding site, but together with the OCH_3_ moiety at the B-atom of **10b** should compensate the lipophilic contribution of the additional ethyl moiety at the bodipy system. The corresponding dimethylpyrrole-derived fluorescent probes **9a** and **10a** already showed promising staining properties [27] (Scheme 1).

The preparation of bodipy-labeled senicapoc derivatives **9** and **10** started with a three-step one-pot synthesis of azido-substituted bodipy derivatives **6a** and **6b**. Condensation of benzaldehyde **4** with two equivalents of pyrroles **5** was followed by DDQ oxidation and reaction with BF_3_ to afford the bodipy derivatives **6a** and **6b** in 13% and 17% yield, respectively. One F-atom of difluoro derivatives **6** was replaced by a methoxy moiety upon activation with TMSOTf and subsequent treatment with methanol. Finally, 1,3-dipolar cycloaddition of senicapoc derived propargyl ether **8** with azido-substituted bodipys **7** and **8** provided the bodipy-labeled senicapoc derivatives **9** and **10**.

In the next series of fluorescent probes, the phenyl moiety attached at the 8-position of the bodipy dyes **9** and **10** together with its oxyethoxyethyl linker should be replaced by a simple alkyl linker. In contrast to probes **9** and **10**, the flexibility of the linker of the envisaged probes **15** and **16** starts directly at the bodipy system. A linker length of four CH_2_ moieties was chosen, mimicking the linker length of successful probes [26] without increasing the lipophilicity too much.

**Scheme 2:**
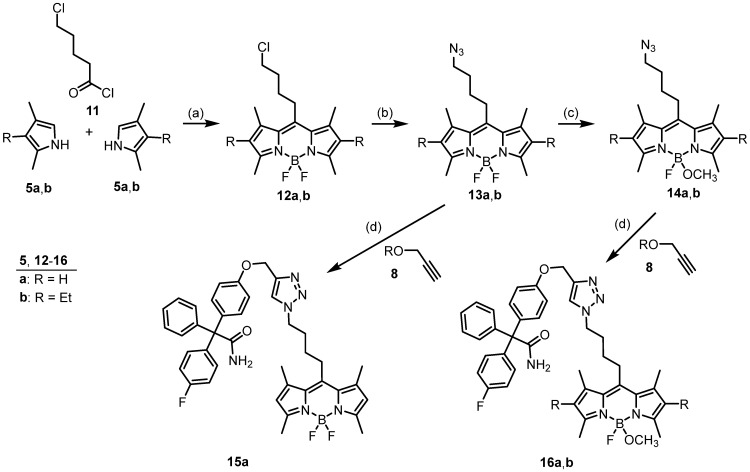
Synthesis of senicapoc derivatives linked with a bodipy dye via an aliphatic linker. Reagents and reaction conditions: (a) 1. CH_2_Cl_2_, 0 °C, 30 min then rt, 30 min; 2. Net_3_, BF_3_ Oet_2_, rt, 14 h, 54% (**12a**), 56% (**12b**). (b) NaN_3_, DMF, 40 °C, 24 h, 88% (**13a**), 50% (**13b**). (c) CH_2_Cl_2_, TMSOTf, CH_3_OH, DIPEA, 0 °C → rt, 20 min, 62% (**14a**), 68% (**14b**). (d) DMF, H_2_O, sodium ascorbate, CuSO_4_, rt, 16 h, 60% (**15a**), 49% (**16a**), 81% (**16b**).

For the synthesis of probes **15** and **16** with a tetramethylene linker, 5-chlorovaleryl chloride (**11**) was reacted with two equivalents of pyrroles **5** and subsequently with BF_3_. Starting with an acid chloride (e.g., **11**) did not require oxidation of the first intermediate, providing the 4-chlorobutyl-substituted bodipy derivatives **12a** and **12b** in 54% and 56% yields, respectively. Nucleophilic substitution of chlorides **12** by azide led to azides **13** and subsequent replacement of one F-atom at the B-atom of **13** by a methoxy moiety yielded monomethoxy derivatives **14**. Cu^+^-catalyzed 1,3-dipolar cycloaddition of propargyl ether **8** with azides **13a** and **14a**,**b** provided the bodipy-labeled senicapoc derivatives **15a** and **16a**,**b** in 49–81% yield (Scheme 2).

In the third series of probes, fluorescein should be used for labeling of senicapoc. Compared to bodipy dyes, fluorescein is more polar, better soluble in aqueous systems, and it shows different absorption (λ_max_ = 485 nm) and emission maxima (λ_max_ = 514 nm).

**Scheme 3:**
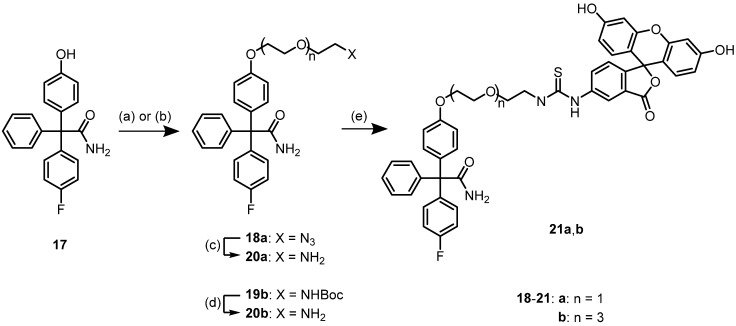
Synthesis of fluorescein-labeled senicapoc derivatives. Reagents and reaction conditions: (a) TsOCH_2_CH_2_OCH_2_CH_2_N_3_, Cs_2_CO_3_, DMF, 80 °C, 16 h, 70%. (b) TsO(CH_2_CH_2_O)_3_CH_2_CH_2_NHBoc, Cs_2_CO_3_, DMF, reflux, 16 h, 100%. (c) H_2_, 5 bar, Pd/C, EtOAc, rt, 1 h, 98%. (d) TFA, CH_2_Cl_2_, rt, 16 h. (e) fluorescein isothiocyanate (FITC), CH_2_Cl_2_, NEt_3_, rt, 16 h, 43% (**8a**), 40% (**8b**, over two steps from **6b**).

Since fluorescein isothiocyanate (FITC) should be used for the introduction of the dye, senicapoc had to be provided with a primary amine. For this purpose, senicapoc-phenol **17** was alkylated with azidoethoxyethyl tosylate to obtain azide **18a**. A linker with four oxyethylene subunits was introduced by alkylation of senicapoc–phenol **17** with the corresponding Boc-protected tosylate to provide Boc-derivative **19b**. Reduction of azide **18a** with H_2_ and Pd/C and cleavage of the Boc moiety of **19b** with trifluoroacetic acid led to primary amines **20a** and **20b**, respectively. Reaction of primary amines **20a** and **20b** with fluorescein isothiocyanate provided thiourea derivatives **21a** and **21b** with different linker lengths between the senicapoc warhead and the fluorescent dye (Scheme 3).

### 3.2. Lipophilicity of Fluorescently Labeled Senicapoc Derivatives

The new fluorescent dyes were designed to increase the polarity and reduce the non-specific labeling of lipophilic membranes of cells. Moreover, a higher polarity should lead to increased water solubility and thus improved handling of the dyes. The logD_7.4_ value was recorded as a measure of the polarity/lipophilicity using our recently published micro-shake flask method. According to this method, the amount of compound in the aqueous 3-morpholinopropanesulfonic acid (MOPS) buffer pH 7.4 was determined by LC-MS [31,32]. Usually, compounds with logD_7.4_ values below 0 exhibit permeability issues, whilst those with a logD7.4 value above 5 have low solubility, complicating their application in aqueous systems [34].

In Table 1, the recorded logD_7.4_ values of the dye-labeled senicapoc derivatives are summarized and compared with the previously reported logD_7.4_ values for **9a** and **10a**. The diethyl-substituted dye **10b** with an additional methoxy moiety at the B-atom could not be detected in the aqueous layer, indicating a logD_7.4_ value > 5. This logD_7.4_ value is higher than the logD_7.4_ values of the bodipy derivatives **9a** and **10a**. Obviously, the methoxy moiety at the B-atom could not compensate the lipophilic contribution of the two ethyl moieties at the bodipy system. The logD_7.4_ value of the difluoro derivative **9b** was not determined, since it was expected that it is even higher than the logD_7.4_ value of monomethoxy derivative **10b**.

Introduction of the lipophilic tetramethylene linker between the triazole and the bodipy core system increased the lipophilicity of **15** and **16** further. Even the least lipophilic dye **16a** could not be detected in the MOPS layer, indicating a logD_7.4_ value > 5.

The idea to increase the polarity and thus the solubility of dye-labeled senicapoc derivatives led to the synthesis of fluorescein-labeled senicapoc derivatives **21a** and **21b**. **21a** with the shorter PEG linker showed a logD_7.4_ value of 1.69, which is in a promising range. Indeed, handling of the fluorescein-labeled senicapoc derivatives **21a** and **21b** is more convenient due to their improved solubility.

In addition to the logD_7.4_ values, the logP values of the fluorescent probes were recorded by HPLC, i.e., after calibration of the method, the retention times were correlated with the logP values or the compounds [33]. For ligands **9**/**10** and **15**/**16**, it was shown that replacement of an F-atom by a OCH_3_ moiety at the B-atom decreased the logP value by one log-unit, whereas introduction of an additional ethyl moiety at the pyrrolidine ring increased the logP value by 1.4 log-units. Fluorescein-labeled ligands **21a** and **21b** are considerably more polar than the corresponding bodipy-labeled senicapoc derivatives. They exhibit very low logP values of 1.5 and 1.3, respectively.

### 3.3. Photophysical Properties of Fluorescently Labeled Senicapoc Derivatives

Fluorescence microscopy was used for the analysis of K_Ca_3.1 channel labeling of cells with fluorescent dyes. Therefore, the photophysical properties of fluorescently labeled senicapoc derivatives **15a**, **21a**, and **22** were determined. Since the fluorophores are separated from the rest of the molecule by a linker, the photophysical properties could be recorded using azide precursors **6a**, **6b**, and **7a** as well. Table 2 displays important photophysical characteristics of the analyzed dyes.

Table 2 clearly indicates that the absorption and emission wavelengths as well as the Stokes shift are independent of the substituents at the B-atom (BF_2_ or BFOCH_3_; compare **6a** and **6b**) and the substituent in the 8-position of the bodipy dye (aryl or alkyl substituent; compare **15a** and **22**). Moreover, it is not relevant whether the data are recorded with the azide precursors or the complete dyes (compare **6a** and **22**). However, the introduction of two ethyl moieties at the bodipy core (**6b**) increased the absorption and emission maxima by 23 nm, but did not influence the Stokes shift (still 13 nm). The fluorescein–senicapoc conjugate 21a slightly reduced the absorption maximum and slightly increased the emission maximum, which overall resulted in a larger Stokes shift of 25 nm.

One alkyl substituent in the 8-position (e.g., **15a**) or two alkyl substituents in 2- and 6-positions (e.g., **6b**) led to increased photoluminescence lifetimes and higher quantum yields. This effect was particularly pronounced for the senicapoc–bodipy conjugate **15a** with an alkyl linker between the bodipy core and the triazole ring: the photoluminescence life time was 5.77 ns and the quantum yield was almost 100% (96%, see Table 2). **6a** with a methoxy moiety at the B-atom and fluorescein conjugate **21a** revealed the lowest quantum yields of these ligands.

### 3.4. Imaging of K_Ca_3.1 Channels in Cells

For the staining experiments, the non-small cell lung cancer cell line A549-3R with a high expression of the K_Ca_3.1 channel was employed [27]. An optimized staining protocol (Protocol 1) described previously was followed to analyze the imaging properties of the fluorescent probes. In brief, the 10 µM solution of the senicapoc–dye conjugate in PBS was added to adherent A549-3R cells in a glass bottom dish and the set-up was incubated for 10 min at 4 °C in a dark chamber. After careful washing, the cells were analyzed with a fluorescence microscope.

Staining of the fixed A549-3R tumor cells with the ethyl-substituted difluorobodipy-labeled senicapoc derivative **9b** and conjugates **15a**, **16a**, and **16b** with an aliphatic tetramethylene linker led to unspecific staining (see Figure S6, Supplementary Materials). Strong green fluorescence was detected around the nucleus of the cells, but a specific punctate staining pattern could not be recognized. Instead, big green fluorescent dots, which are larger than expected for a single ion channel, were observed. A similar observation was made with the more polar fluorescein-labeled senicapoc derivatives **21a** and **21b**. A weak green fluorescence of the membrane and an intense staining of the nucleus were observed (see Figure S7, Supplementary Materials).

**Figure 3:**
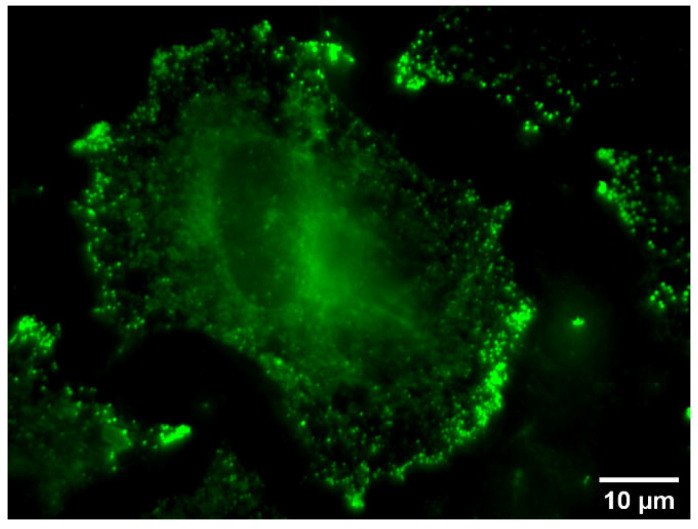
Staining of fixed A549-3R tumor cells with bodipy-labeled senicapoc derivative **10b**.

However, promising results were obtained after incubation of the fixed A549-3R tumor cells with the bodipy-labeled senicapoc derivative **10b** showing the expected punctate staining pattern of the membrane (Figure 3). The images were analyzed with respect to size and density of the fluorescent dots. For this purpose, a square with a size of 50 × 50 pixels was randomly placed on the cell images using ImageJ. The intensities of the signals within the square were analyzed to calculate the Full Width at Half Maximum (FWHM) for each selected dot, which represents a measure for the signal size. For the accurate representation of a single K_Ca_3.1 channel, the FWHM should be in the range of five pixels. Signals with a FWHM of ≤5 pixels within the square were regarded as labeled K_Ca_3.1 channels and the number of identified channels was used to determine the density of the ion channels on the cells (Figure S8, Supplementary Materials). Analysis of the images obtained by staining of fixed A549-3R cells with **10b** resulted in a FWHM of 5.27 ± 0.22 pixels and a density of 1.22 ± 0.26 K_Ca_3.1 channels per µm^2^.

As negative control, HEK293 cells, which do not express K_Ca_3.1 channels, were incubated with bodipy-labeled senicapoc derivative **10b**. As expected, a punctate staining pattern was not observed (Figure S9, Supplementary Materials). In a second control experiment, azide precursor **7b** without the senicapoc warhead was incubated with fixed A549-3R tumor cells. The cells showed high fluorescence intensity inside and around the nucleus. However, a punctate staining pattern characteristic for the interaction with the ion channel was not observed, since the targeting senicapoc unit was missing (Figure S10, Supplementary Materials).

**Figure 4:**
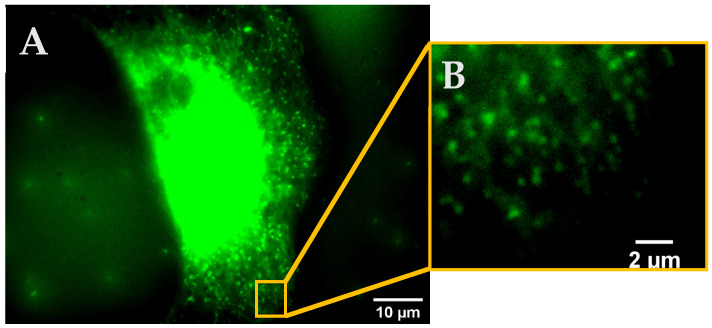
(**A**) Staining of living A549-3R tumor cells with bodipy-labeled senicapoc derivative **10b** (protocol 2). (**B**) Magnification of the marked square.

In the next experiment, living A549-3R cells were stained with **10b**. Staining was performed as described above (Protocol 2), leading to a distinct punctate staining pattern. Analysis of the image as detailed above resulted in a FWHM of 4.89 ± 0.50 pixels and a density of 1.11 ± 0.11 K_Ca_3.1 channels per µm^2^ (Figure 4).

In order to show the specificity of labeling K_Ca_3.1 channels by the fluorescent probe **10b**, fixed and living cells were preincubated with 30 µM senicapoc (3) for 5 min at 4 °C. Subsequent treatment of the cells with bodipy-labeled senicapoc derivative **10b** did not lead to a punctate staining pattern neither for fixed nor for living A549-3R tumor cells (Figure S11, Supplementary Materials). The complete blockade of the punctate staining pattern confirms that the K_Ca_3.1 channel was labeled with probe **10b**.

The specificity of the bodipy-labeled senicapoc derivative **10b** was further analyzed via conducting co-staining experiments with antibodies. For staining with antibodies, A549-3R cells had to be permeabilized with saponin, since the antibody binds at the intracellular part of the ion channel only after entering the cell [35]. Labeling of the K_Ca_3.1 channel of permeabilized cells with fluorescent dye **10b** led to the expected punctate staining pattern with a density of 1.15 ion channels/µm^2^ (see Figure S12, Supplementary Materials). For antibody-based immunofluorescence staining of the K_Ca_3.1 channels of permeabilized A549-3R cells, various concentrations of the primary and secondary antibody were tested. Optimal images were obtained with a dilution of 1:300 for the primary antibody and 1:500 for the secondary antibody (see Figure S13, Supplementary Materials). The images obtained by immunofluorescence staining were analyzed as described above for the images with fluorescent probe **10b**. This analysis led to a FWHM of 4.80 ± 0.83 pixels and a density of 1.75 ± 0.19 K_Ca_3.1 channels/µm^2^. It can be concluded that the FWHM obtained with **10b** staining (5.27 pixels) is in the same range as the FWHM obtained with immunofluorescence (4.80 pixels), but the density of the ion channel was higher after staining with the antibodies (1.75 channels/µm^2^) compared to 1.22 channels/µm^2^ after staining with **10b**.

**Figure 5:**
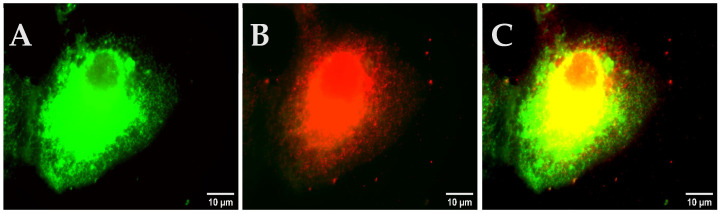
Co-staining of permeabilized A549-3R tumor cells first with antibodies (anti-K_Ca_3.1 and cy3-labeled goat anti-rabbit antibody) (red) and second with bodipy-labeled senicapoc derivative **10b** (green). (**A**) Analysis of **10b** fluorescence using the FITC filter set. (**B**) Analysis of the fluorescence antibody binding using the TRITC filter set. (**C**) Merged images of (**A**,**B**).

**Figure 6:**
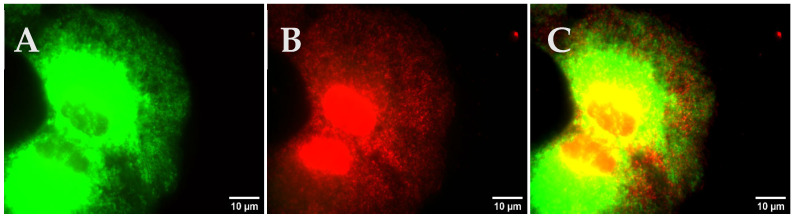
Co-staining of permeabilized A549-3R tumor cells first with bodipy-labeled senicapoc derivative **10b** (green) and second with antibodies (anti-K_Ca_3.1 and cy3-labeled goat anti-rabbit antibody) (red). (**A**) Analysis of **10b** fluorescence using the FITC filter set. (**B**) Analysis of the fluorescence antibody binding using the TRITC filter set. (**C**) Merged images of (**A**,**B**).

For the co-staining experiments, the permeabilized A549-3R cells were first incubated with the antibodies and subsequently stained with bodipy-labeled senicapoc derivative **10b** for 10 min (protocol 7a). The images were analyzed with the FITC filter set to observe the fluorescence generated by **10b** and the TRITC filter set to observe the fluorescence emitted by the secondary antibody (Figure 5). In a further experiment, the order of staining modalities was reversed, i.e., the cells were first labeled with fluorescent probe **10b** for 10 min and second with the antibodies (Figure 6).

The obtained green, red and yellow dots were analyzed as described in the cell staining experiments above. The density of antibody-stained K_Ca_3.1 channels (red dots) was higher than the density of K_Ca_3.1 channels stained with bodipy-labeled senicapoc derivative **10b** independent of the order of staining modality (Table 3). Co-staining of a single K_Ca_3.1 channel with both imaging tools visualized by yellow dots was hardly observed. A successful co-staining was only counted when the distance between the signal peak maxima was ≤1 pixel [36].

It was concluded that the K_Ca_3.1 channels accept only one imaging modality, either the fluorescent probe or the antibody. This observation supports the hypothesis that the bodipy-labeled senicapoc derivative **10b** can only interact with K_ca_3.1 channels in the open state, as it has to enter the ion channel to reach its binding site within the pore. On the other hand, the primary antibody can only react with the K_Ca_3.1 channel in its closed conformation, which inhibits the fluorescent probe reaching its binding site. Moreover, in the open conformation, the binding site for the antibody is occupied by calmodulin [35].

Table 3 reveals a ratio of ion channels labeled with senicapoc–dye conjugate **10b** (green) and antibodies (red) of approximately 41.5:58.5. This ratio reflects the ratio of K_Ca_3.1 channels in open and closed conformations. According to the literature, the maximal open probability of the K_Ca_3.1 channel at saturating Ca^2+^ concentrations is 44% [37], which correlates well with these imaging results.

### 3.5. Patch Clamp Experiments with Fluorescent Probe **10b**

In order to analyze the channel inhibitory activity of fluorescent probe **10b**, patch clamp experiments were conducted using whole A549-3R cells. As senicapoc acts an inhibitor at K_Ca_3.1 channels, it was expected that the bodipy-labeled senicapoc derivative **10b** behaves as an inhibitor as well. For measuring inhibitory activity, the ion channel was opened with the positive K_ca_3.1 modulator 1-ethylbenzimidazolone (1-EBIO). At a concentration of 1 µM, **10b** inhibited the ion channel considerably. Its inhibitory activity was only slightly lower than the inhibitory activity of senicapoc (see Figure S14, Supplementary Materials). It was concluded that the bodipy-conjugated senicapoc derivative **10b** was able to bind at the senicapoc binding site within the ion channel pore of the K_Ca_3.1 channel inhibiting the ionic current across the cell membrane.

## 4. Conclusions

In order to further improve fluorescent probes to image K_Ca_3.1 channels, senicapoc was coupled with various fluorescent dyes. Connecting the bodipy fluorophore via an aliphatic linker increased the polarity of the probes but did not lead to labeling of single ion channels. Introduction of fluorescein instead of a bodipy dye increased the polarity considerably, but the probes were not suitable for single-channel labeling. Bodipy dyes with an additional ethyl moiety at the pyrrole structure showed increased quantum yields but higher lipophilicity leading to unspecific labeling of the membrane. However, replacement of one F-atom at the B-atom by a OCH_3_ moiety compensated for the increased lipophilicity of the probes. Thus, ethylbodipy-labeled senicapoc derivative **10b** with a OCH_3_ moiety at the B-atom displayed excellent imaging properties.

**10b** was able to selectively label single K_Ca_3.1 channels of fixed and living non-small cell lung cancer cells A549-3R. The specificity was shown with HEK cells, which do not express the K_Ca_3.1 channel, by preincubation with senicapoc blocking the ion channel and by using the azide precursor **7b** for staining. Moreover, **10b** inhibited the ion current after activation of the K_Ca_3.1 channel with selective channel opener 1-EBIO.

Costaining of A540-3R tumor cells with **10b** and antibodies resulted in non-overlapping signals. The ratio of channels labeled with **10b** and with antibodies was 41.5:58.5, independent of the order of addition of staining solutions. This ratio reflects the natural ratio of the channel in open and closed conformations. Antibodies cannot interact with the open conformation of the ion channel, since their binding site is occupied by calmodulin. On the other hand, bodipy-labeled senicapoc derivative **10b** has to enter the open ion channel from the cytoplasmic side to reach its binding site within the ion channel pore.

Altogether, the newly developed senicapoc–bodipy conjugate **10b** represents a useful imaging tool for fast and efficient labeling of K_Ca_3.1 channels in fixed, permeabilized and living cells. Compared to staining with antibodies, staining with **10b** is cheaper and faster (10 min instead of 6 h). Moreover, **10b** can label K_Ca_3.1 channels of non-permeabilized cells. Since antibodies bind at the cytosolic part of the K_Ca_3.1 channel, they have to enter the cell before labeling the ion channel. Thus, they are not able to interact with K_ca_3.1 channels of living cells.

## Figures and Tables

**Table 1:** logD_7.4_ values of selected bodipy- and fluorescein-labeled senicapoc derivatives.

Compd.	logD_7.4_ ± SD (Micro Shake Flask)	logP (HPLC)
**9a** [27]	4.73 ± 0.03	5.4
**9b**	nd	6.7
**10a** [27]	4.60 ± 0.23	4.4
**10b**	>5	5.8
**15a**	nd	4.8
**16a**	>5	4.0
**16b**	nd	5.4
**21a**	1.69 ± 0.04	1.5 ^#^
**21b**	nd	1.3 _#_

SD: standard deviation. nd: not determined. ^#^ HPLC at pH 6.0.

**Table 2:** Photophysical properties of azide precursors **6a**, **6b**, **7a** and complete imaging probes **15a**, **21a** and **22**.

Compd.	Compd. Characteristics	Absorption *λ*_max_ (nm)	Emission *λ*_max_ (nm)	Stokes Shift (nm)	*τ*^a^ (ns)	*Φ*_F_^b^
**6a** [27]	BF_2_	501	515	14	3.80 ± 0.02	0.48 ± 0.02
**6b ^c^** [26]	BF_2_, Et ^e^	524	537	13	6.32 ± 0.05	0.81 ± 0.02
**7a** [27]	BFOCH_3_	501	516	15	3.50 ± 0.01	0.43 ± 0.02
**15a**	BF_2_, -(CH_2_)_4_-	501	513	12	5.77 ± 0.02	0.96 ± 0.04
**21a** ^d^	Fluorescein	496	521	25	4.21 ± 0.02	0.32 ± 0.02
**22** [26]	BF_2_^e^	501	512	11	3.38 ± 0.01	0.62 ± 0.03

Data were recorded in solution (CH_2_Cl_2_, CH_3_CN (**15**) or H_2_O (**21a**) *c* = 10^−5^ M) at 298 K. The structure of the previously reported senicapoc–bodipy conjugate **22** is shown in Figure S1, Supplementary Materials. Selected absorption and emission spectra are shown in Figures S2–S5, Supplementary Materials. ^a^ *τ*: photoluminescence lifetime. ^b^ *Φ*_F_: absolute quantum yield. ^c^ measured in CH_3_CN. ^d^ measured in H_2_O. ^e^ CH_2_CH_2_O linker instead of CH_2_CH_2_OCH_2_CH_2_O linker.

**Table 3:** Average of green and red signals after co-staining with fluorescent dye **10b** and antibodies (Protocols 7a and 7b).

Compd.	Staining Protocol	Average Green (in % ± SD)	Average Red (in % ± SD)
10b	7a (antibody first)	41.5 ± 4.75%	58.5 ± 9.60%
10b	7b (**10b** first)	41.4 ± 5.56%	58.6 ± 8.52%

## Data Availability

Most of the original data are given in the manuscript and the Supplementary Materials. Further details will be made available on request.

## Supplementary Materials

The following supporting information can be downloaded at: https://www.mdpi.com/article/10.3390/pharmaceutics17020154/s1. Supporting Information contains detailed information about the photophysical properties of the novel senicapoc dye conjugates, the analysis of single dots, figures of different imaging experiments, details of the patch clamp experiments, all ^1^H and ^13^C NMR spectra as well as all HPLC chromatograms.

## Abbreviations

APCI	atmospheric pressure chemical ionization
DDQ	2,3-dichloro-5,6-dicyano-1,4-benzoquinone
DIPEA	diisopropylethylamine
DMSO	dimethyl sulfoxide
1-EBIO	1-ethylbenzimidazolone
FITC	fluorescein isothiocyanate
FWHM	full width at half maximum height
MOPS	3-morpholinoproanesuofonic acid
PMA	photomultiplier assembly
SD	standard deviation
SNAP	name for the columns used for purification
TFA	trifluoroacetic acid
TRITC	5/6-tetramethylrhodamine isothiocyanate
