# Supplementary material for: Fluorescent Probes to Image the KCa3.1 Channel in Tumor Cells
Source: Pharmaceutics. 2025 Jan 23;17(2):154. doi: 10.3390/pharmaceutics17020154 (PMC11859423; doi:10.3390/pharmaceutics17020154)
Supplement: Supplementary file 1 [file pharmaceutics-17-00154-s001.zip › pharmaceutics-3410529-supplementary.pdf]

Supporting Information

Fluorescent probes to image the $K_{Ca}3.1$ channel in tumor cells

Insa Thale,[a,b] Elke Naß,[c] Laura Vinnenberg,[a,c] Luca Matteo Todesca,[a,d,e] Thomas Budde,[a,c] Iván Maisuls,[f] Cristian A. Strassert,[f] Albrecht Schwab,[a,d] Bernhard Wünsch* [a,b]

a Universität Münster, GRK 2515, Chemical biology of ion channels (Chembion), Corrensstraße 48, D-48149 Münster, Germany

b Universität Münster, Institut für Pharmazeutische und Medizinische Chemie, Corrensstraße 48, D-48149 Münster, Germany

c Universität Münster, Universitätsklinikum Münster, Institut für Physiologie I, Robert-Koch-Straße 27a, D-48149 Münster, Germany

d Universität Münster, Universitätsklinikum Münster, Institut für Physiologie II, Robert-Koch-Straße 27b, D-48149 Münster, Germany

e Current address: University of Padua, Department of Biology, VIA U. BASSI, 58/B

f Universität Münster, Institut für Anorganische und Analytische Chemie, CiMIC, SoN, Corrensstraße 28, D-48149 Münster, Germany; Universität Münster, CeNTech, Heisenbergstraße 11, 48149, Münster, Germany.

Outline

Outline	page
1. Photophysical properties of dye-labeled senicapoc derivatives	S2
2. Cel staining with senicapoc-dye conjugates	S5
3. Analysis of single dots	S6
4. Staining of HEK293 cells with **10b**	S8
5. Staining of A549-3R cells with azide precursor **7b**	S8
6. Blocking experiments by preincubation of senicapoc	S9
7. Staining of permeabilized A549-3R tumor cells	S9
8. Immunofluorescence staining of permeabilized A549-3R tumor cells	S10
9. Patch clamp experiments witn **10b**	S11
10. References	S11
11. ^{1}H and ^{13}C-NMR spectra	S12
12. HPLC chromatograms	S29

1. Photophysical properties of dye-labeled senicapoc derivatives

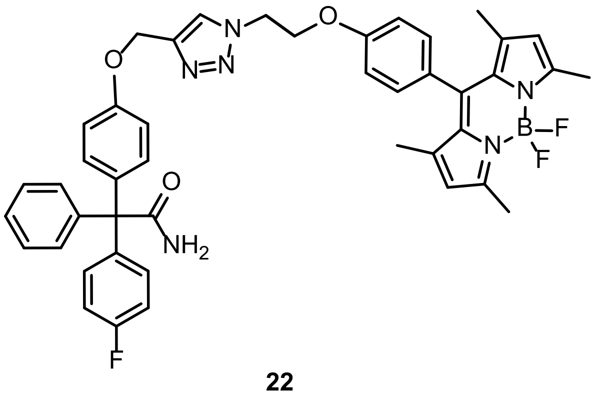

22

Figure S1: Structure of previously reported probe **22**.[1]

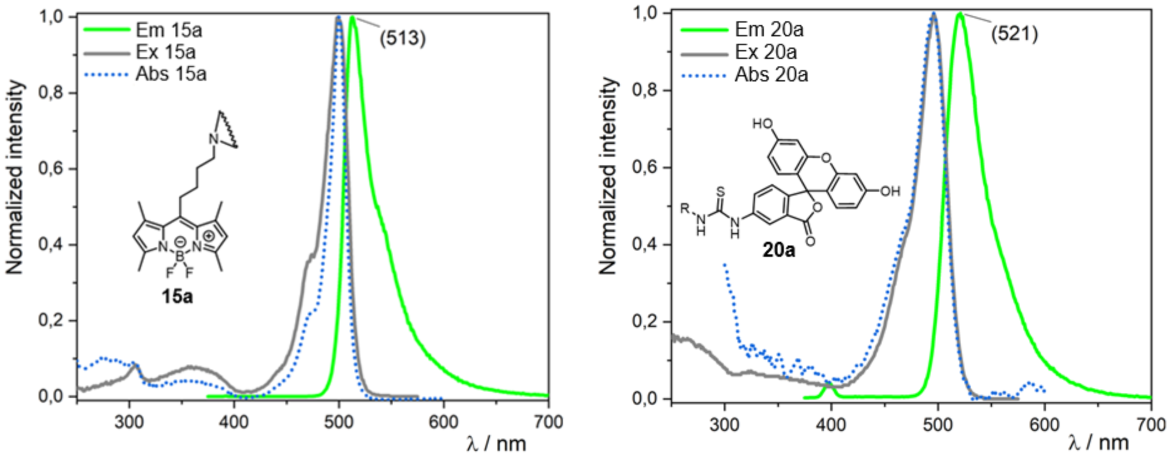

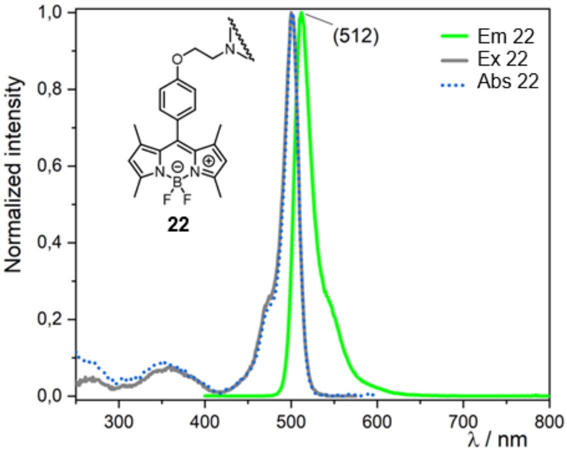

Figure S2: Absorption, excitation and emission spectra in liquid CH_2Cl_2 at 298 K of fluorescent probes **15a**, **20a**, and **22** (c = 10^{-5} M). For all samples λ_{ex} = 350 nm.

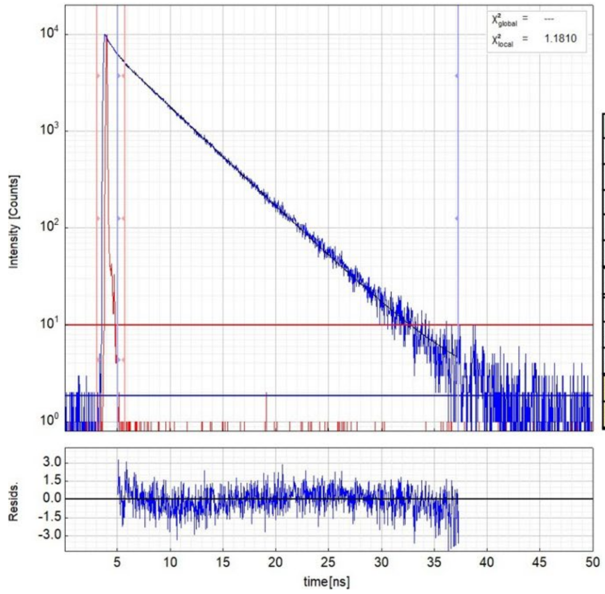

Parameter	Value	Δ	δ
A_1[kCnts/Chnl]	7.70	±0.13	1.7%
τ_1[ns]	4.213	±0.021	0.5%
I_1[kCnts]	1 014	±13	1.2%
A_{Rel1}[%]	100.0	---	---
I_{Rel1}[%]	100.0	---	---
$Bkgr_{Dec}$[kCnts]	0.0019	±0.0004	17%
$Bkgr_{IRF}$[Cnts/Chnl]	10.0000	---	---
$Shift_{IRF}$[ps]	265	±38	14%
A_{Scatt}[kCnts]	-1 020	±180	17%
τ_{AvInt}[ns]	4.213	±0.021	0.5%
τ_{AvAmp}[ns]	4.213	±0.021	0.5%

Figure S3: Left: Raw (experimental) time-resolved photoluminescence decay of **15a** in liquid CH_2Cl_2 at 298 K (air-equilibrated, blue), including the residuals (λ_{ex} = 376, λ_{em} = 515 nm) and the instrumental response function (IRF, red). Right: Fitting parameters including pre-exponential factors and confidence limits.

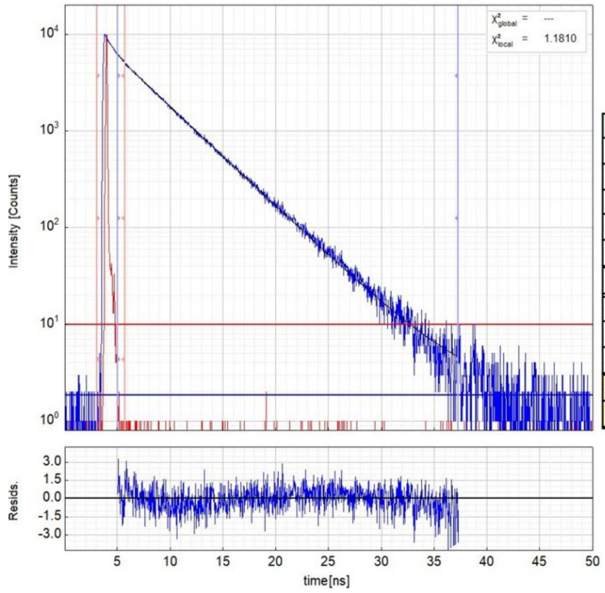

Parameter	Value	Δ	δ
A_1[kCnts/Chnl]	7.70	±0.13	1.7%
τ_1[ns]	4.213	±0.021	0.5%
I_1[kCnts]	1 014	±13	1.2%
A_{Rel1}[%]	100.0	---	---
I_{Rel1}[%]	100.0	---	---
$Bkgr_{Dec}$[kCnts]	0.0019	±0.0004	17%
$Bkgr_{IRF}$[Cnts/Chnl]	10.0000	---	---
$Shift_{IRF}$[ps]	265	±38	14%
A_{Scatt}[kCnts]	-1 020	±180	17%
τ_{AvInt}[ns]	4.213	±0.021	0.5%
τ_{AvAmp}[ns]	4.213	±0.021	0.5%

Figure S4: Left: Raw (experimental) time-resolved photoluminescence decay of **21a** in liquid CH_2Cl_2 at 298 K (air-equilibrated, blue), including the residuals (λ_{ex} = 376, λ_{em} = 520 nm) and the instrumental response function (IRF, red). Right: Fitting parameters including pre-exponential factors and confidence limits.

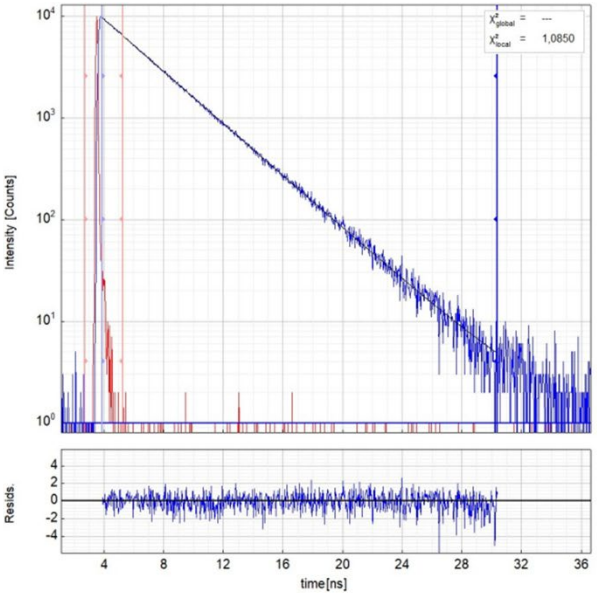

Parameter	Value	Δ	δ
A_1[kCnts/Chnl]	10,55	±0,18	1,7%
τ_1[ns]	3,3833	±0,0078	0,2%
I_1[kCnts]	1 115	±17	1,5%
A_{Rel1}[%]	100,0	---	---
I_{Rel1}[%]	100,0	---	---
$Bkgr_{Dec}$[kCnts]	0,0010	±0,0004	31%
$Bkgr_{IRF}$[Cnts/Chnl]	-15,3	±5,2	34%
$Shift_{IRF}$[ps]	-93	±30	31%
A_{Scatt}[kCnts]	266	±43	16%
τ_{AvInt}[ns]	3,3833	±0,0078	0,2%
τ_{AvAmp}[ns]	3,3833	±0,0078	0,2%

Figure S5: Left: Raw (experimental) time-resolved photoluminescence decay of **22** in liquid CH_2Cl_2 at 298 K (air-equilibrated, blue), including the residuals (λ_{ex} = 376, λ_{em} = 515 nm) and the instrumental response function (IRF, red). Right: Fitting parameters including pre-exponential factors and confidence limits.

2. Cell staining with senicapoc-dye conjugates

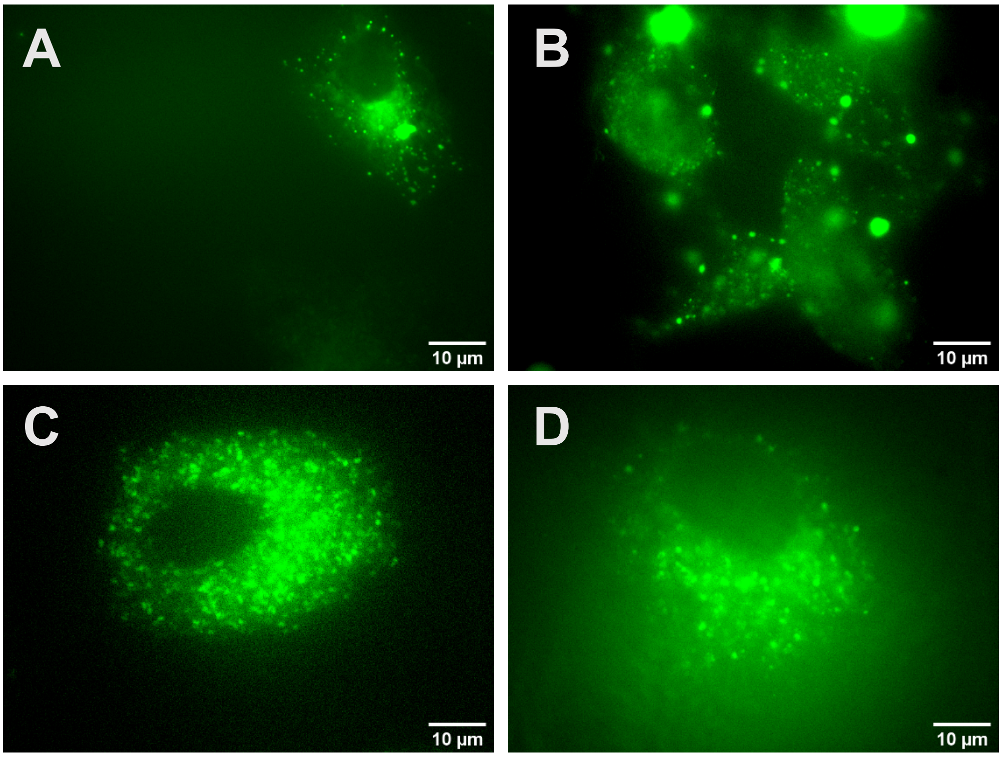

Figure S6: Staining of A549-3R tumor cells with **9b** (A), **15a** (B), **16a**,(C) and **16b** (D) led to unspecific labeling of the cells.

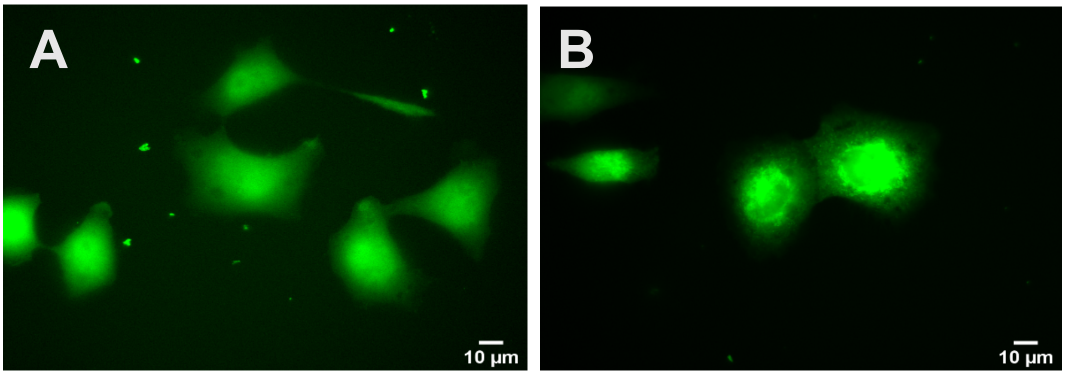

Figure S7: Staining of A549-3R tumor cells with fluorescein-laeled senicapoc derivatives **21a** (A) and **21b** (B). The fluorescein-labeled senicapoc derivatives **21a** and **21b** were imaged with an x40 oil objective of the TIRF microscope.

3. Exemplary analysis of single dots

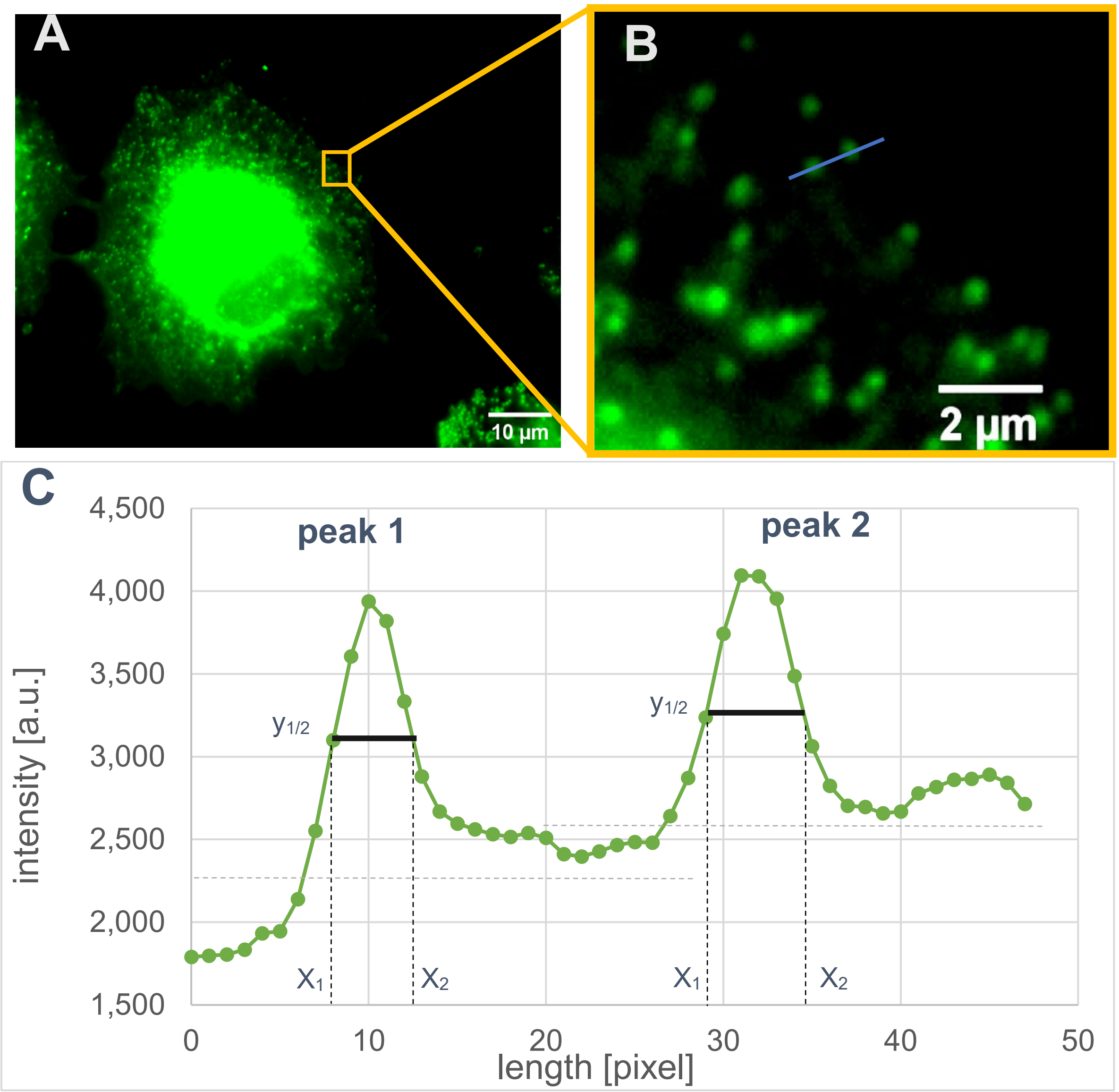

Figure S8: Example for the analysis of the FWHM of single dots.

In Figure S5 the evaluation of the single dots is shown exemplarily. Figure S5A shows the punctate staining pattern of the membrane. Magnification of the dots in Figure S5A leads to Figure S5B showing single dots for single ion channels. Measuring the fluorescence intensity along the line shown in Figure S5B results in the signal intensity of two dots, which is displayed in Figure S5C. The x-axis in Figure S5C represents the length in pixel and the y-axis the fluorescent intensity of the signal. This figure allows the calculation of the “full width at half maximal height (FWHM)” of the dignal.

With the height of the baseline and the maximum of the intensity, the half maximum of the signal was calculated by the following equation.

$$y_{1/2} = [0.5\ (I_{max} + I_{min})]$$

I_{max}: Intensity maximum
I_{min}: Intensity baseline

The width of the signals can be measured in pixel at the determined position. Table S1 displays the calculated FWHM of the two selected signals as well as the fluorescent intensity of the signals.

Table S1: Results of the FWHM calculation of the two selected signals displayed in Figure S5.

peak	$y_{1/2}$ (intensity)	X_1	X_2	FWHM [pixel]
peak 1	3152.5	8.07	12.46	4.39
peak 2	3335	29.22	34.39	5.17

4. Staining of HEK293 cells with 10b

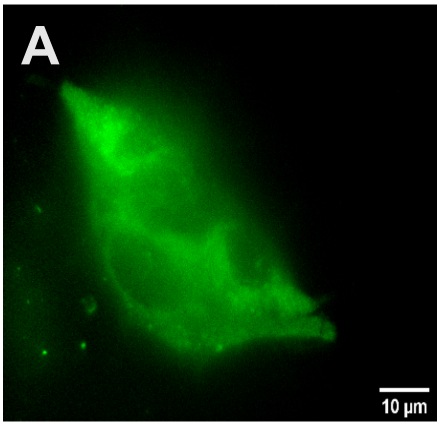

Figure S9: Imaging of HEK293 cells with bodipy-conjugated senicapoc derivative **10b**. Since HEK293 cells do not express $K_{Ca}3.1$ ion channels, a punctate staining pattern could not be observed. For visualization of the cells, a longer exposure time was necessary, since the HEK203 cells showed a low uptake of the fluorescent dye.

5. Staining of A549-3R cells with azide precursor 7b

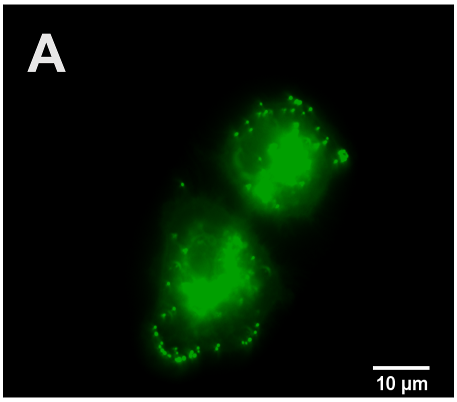

Figure S10: Imaging of A549-3R tumor cells with azide precursor **7b**. The cells showed a high fluorescence intensity inside and around the nucleus and some big green fluorescent dots at the cell membrane, but a punctate staining pattern could not be observed.

6. Blocking experiments by preincubation of senicapoc

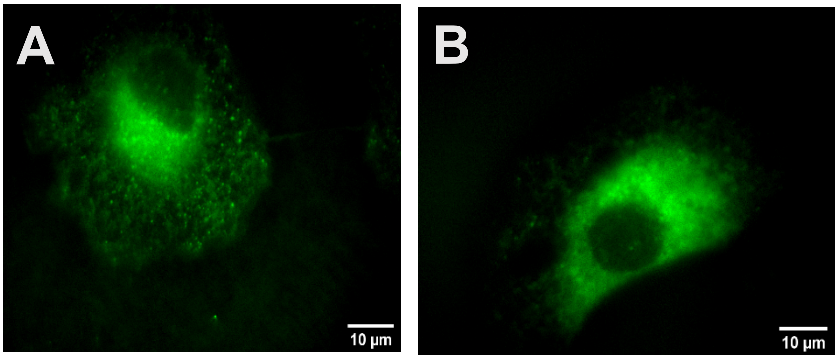

Figure S11: Imaging of fixed (A) and living (B) A549-3R tumor cells with **10b** after preincubation with senicapoc.

7. Staining of permeabilized A549-3R tumor cells with 10b

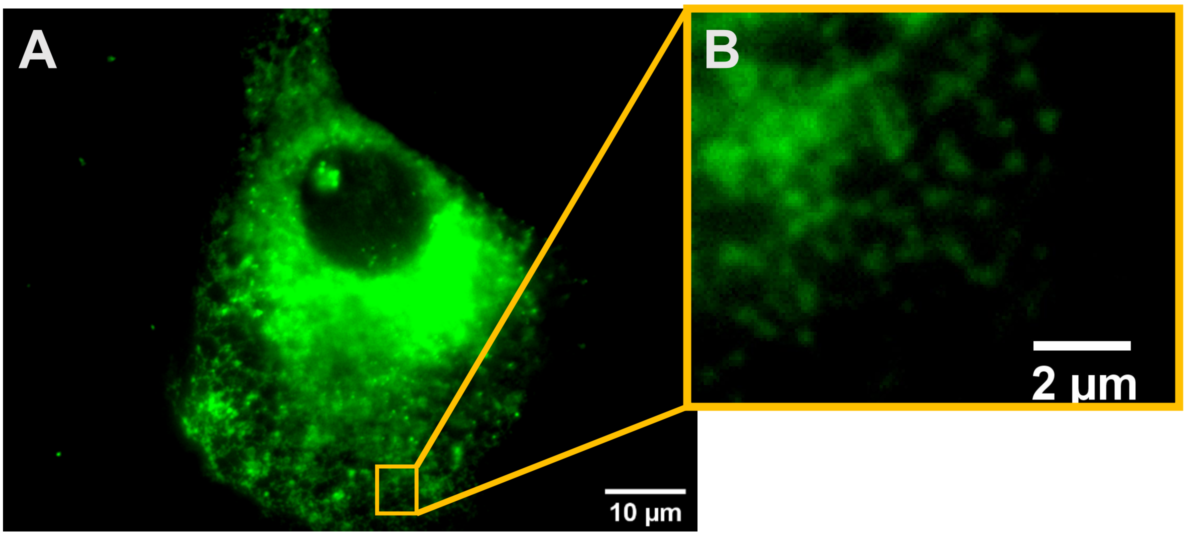

Figure S12: (A) Imaging of permeabilued A549-3R tumor cells with **10b**. (B) Magnification of the marked square.

8. Immunofluorescence staining of permeabilized A549-3R tumor cells

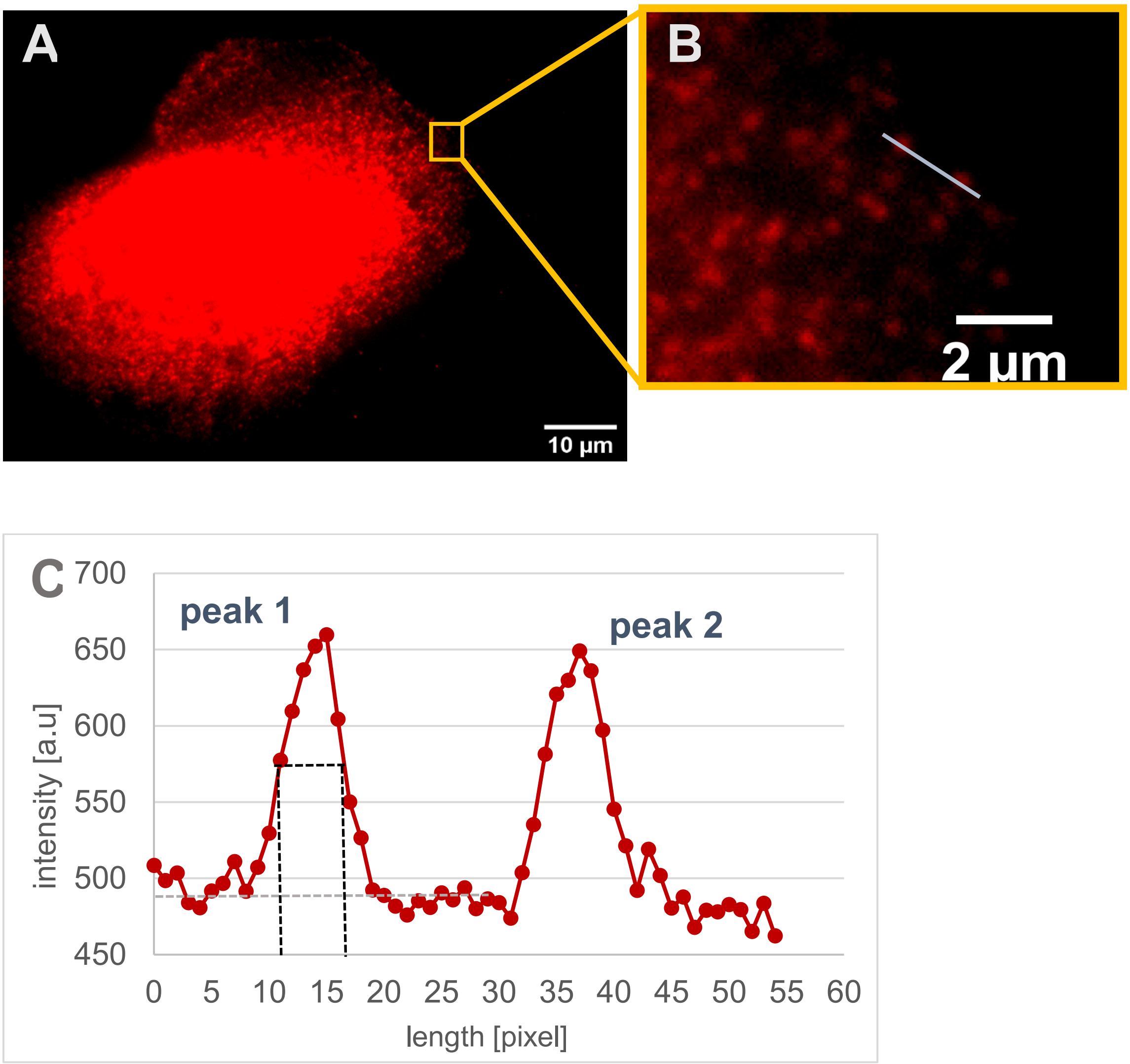

Figure S13: (A) Imaging of permeabilued A549-3R tumor cells with antibodies. Primary antibody 1 : 300, second antibody 1 : 500. (B) Magnification of the marked square. (C) Exemplary presentation of the analysis of a single dots. Peak 1: $y_{1/2}$ = 571, x = 16.3 – 10.9 = 5.4 pixels.

9. Parch clamp experiments with 10b

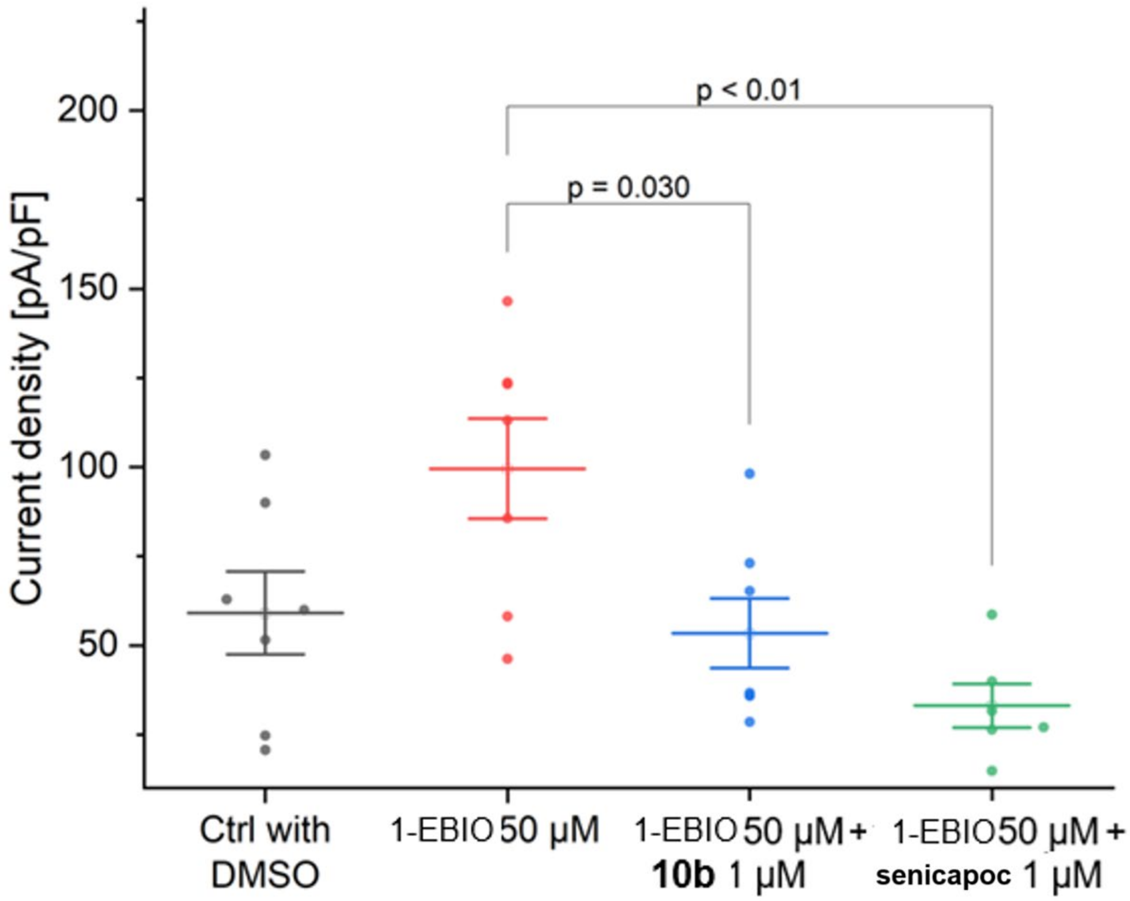

Figure S14: Patch clamp results of bodipy-labeled senicapoc derivative **10b**. Current density [pA/pF] ± SEM; n = 6.

10. References

[1] Brömmel, K.; Maskri, S.; Maisuls, I.; Konken, C.P.; Rieke, M.; Pethö, Z.; Strassert, C.A.; Koch, O.; Schwab, A.; Wünsch, B. Synthesis of small-molecule fluorescent probes for the in vitro imaging of calcium-activated potassium channel $K_{Ca}3.1$. *Angew. Chem.* **2020**, *132*, 8354–8361; *Angew. Chem. Int. Ed.* **2020**, *59*, 8277–8284.

11. ^{1}H and ^{13}C NMR spectra

^{1}H NMR spectrum of epoxide **6b** in $CDCl_3$

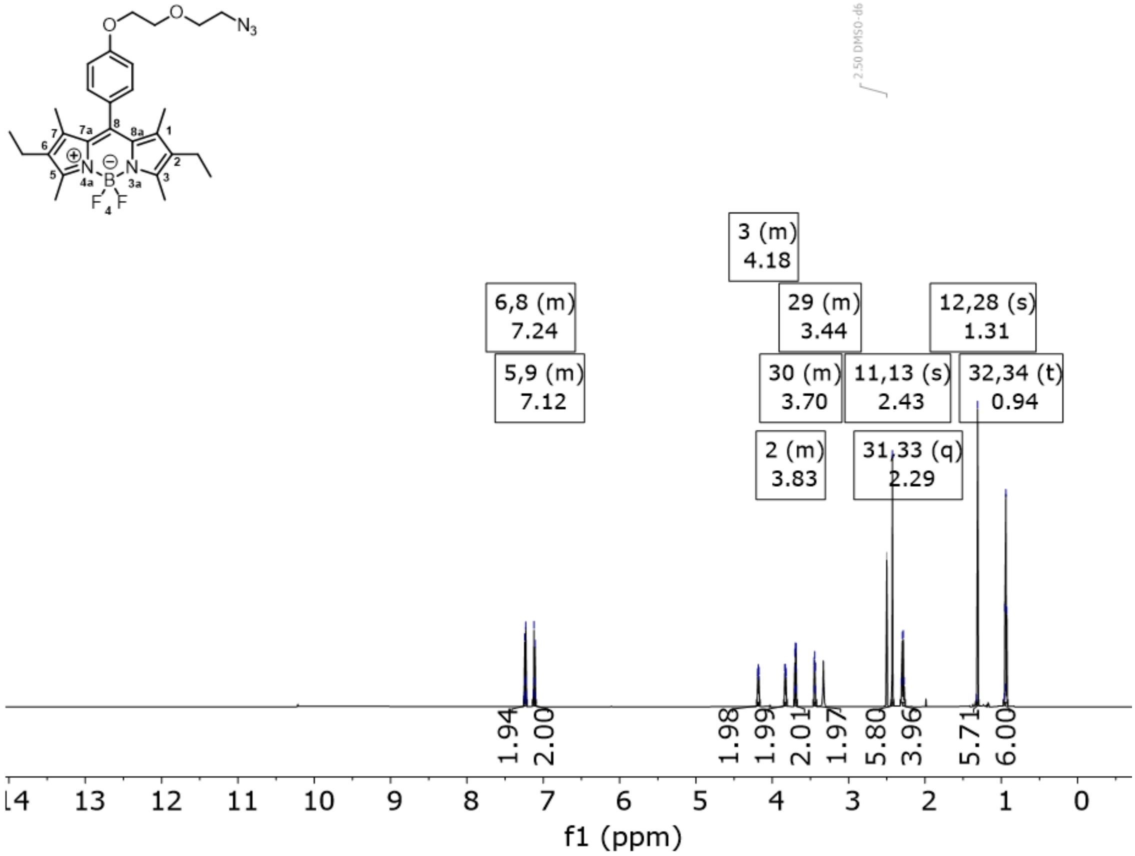

^{13}C NMR spectrum of epoxide **6b** in $CDCl_3$

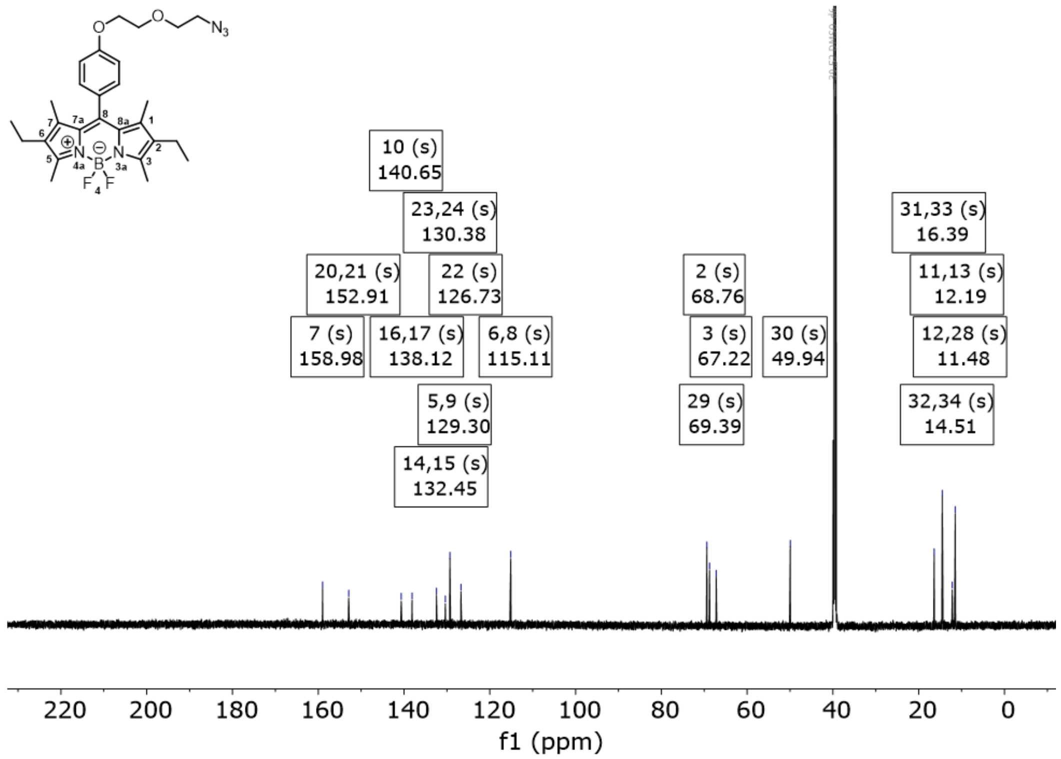

^{1}H NMR spectrum of epoxide **7b** in $CDCl_3$

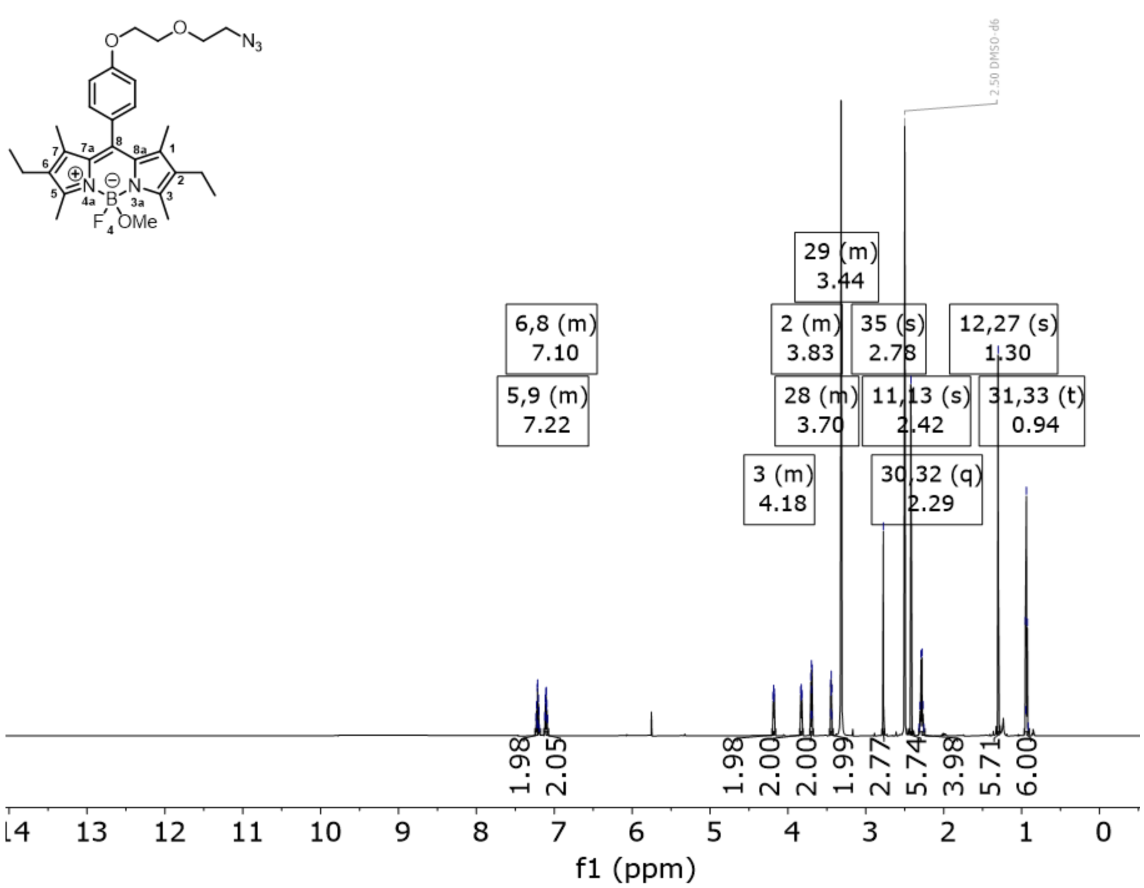

^{13}C NMR spectrum of epoxide **7b** in $CDCl_3$

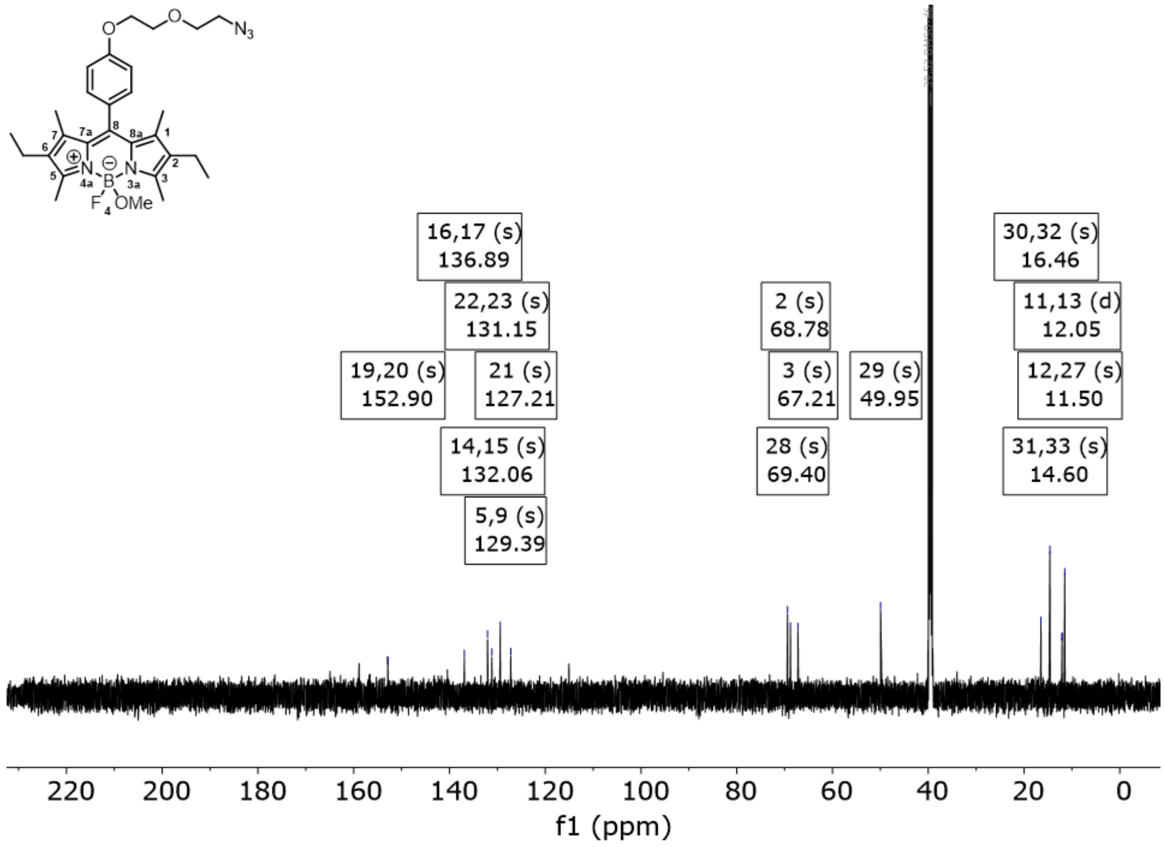

^{1}H NMR spectrum of epoxide **9b** in $CDCl_3$

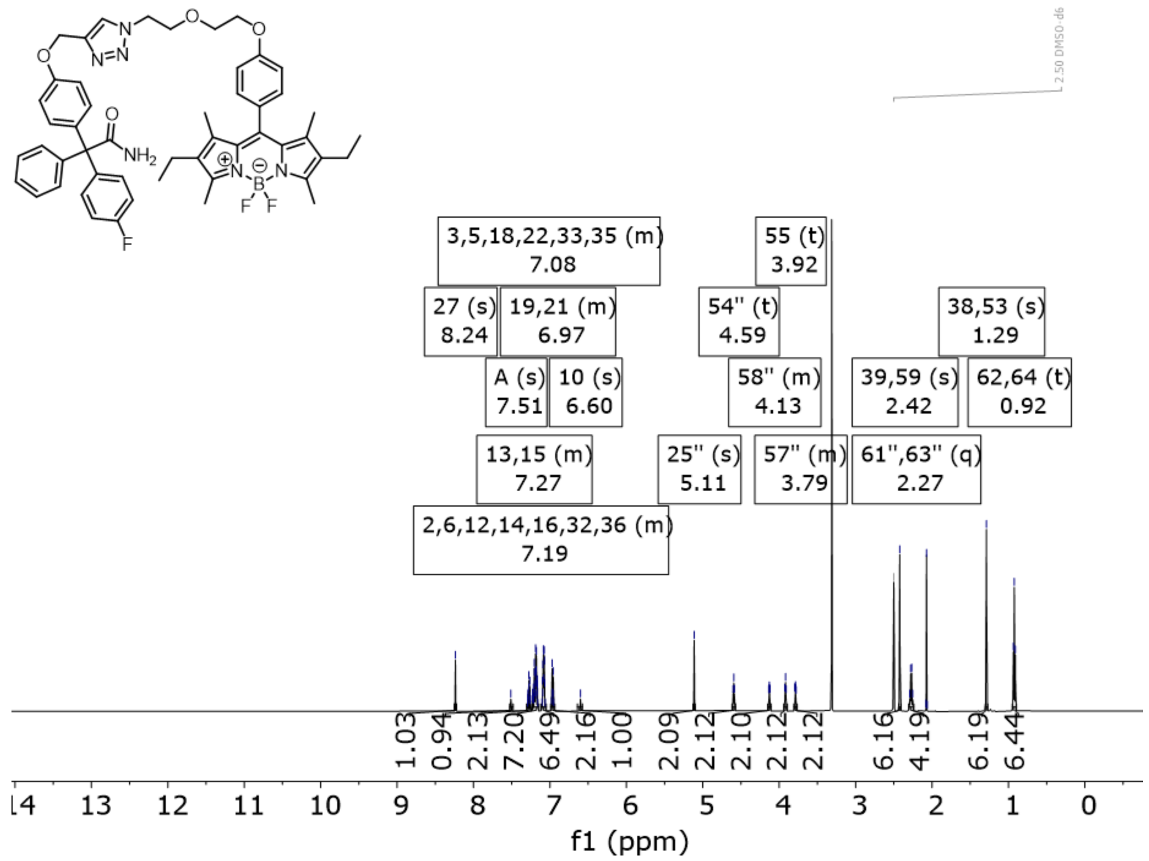

^{13}C NMR spectrum of epoxide **9b** in $CDCl_3$

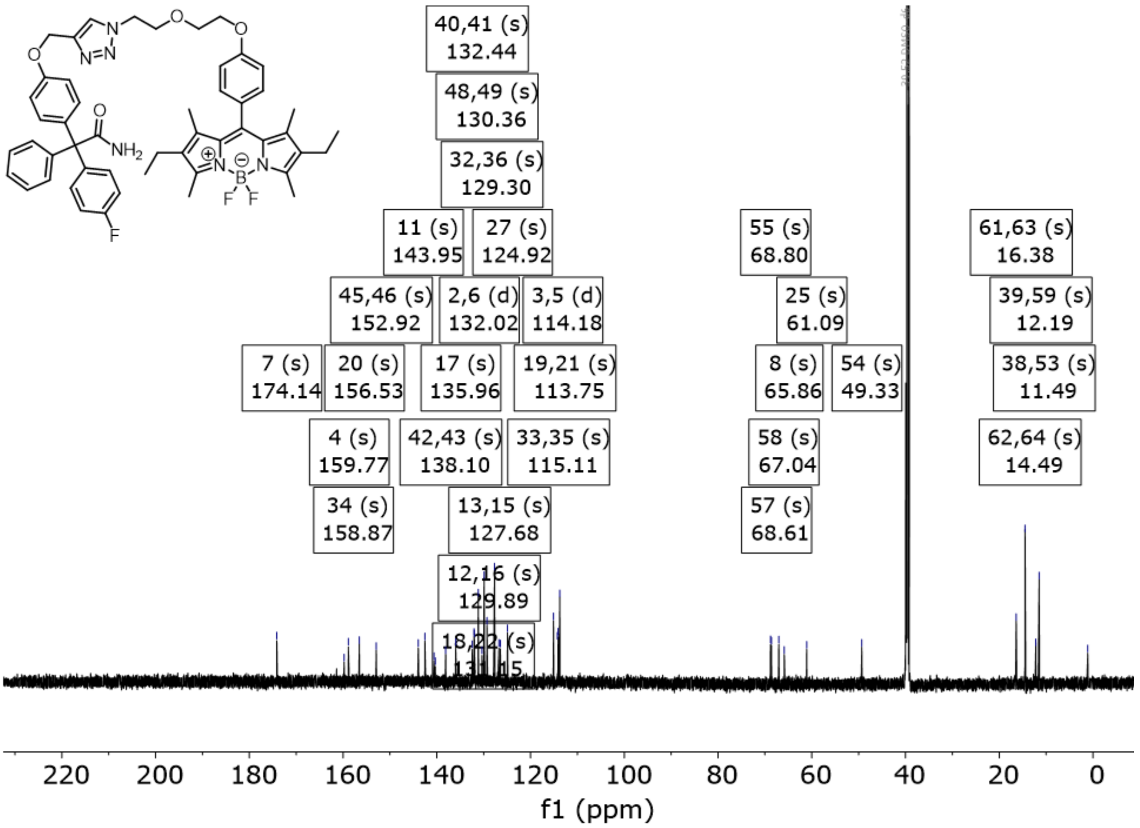

^{1}H NMR spectrum of epoxide **10b** in $CDCl_3$

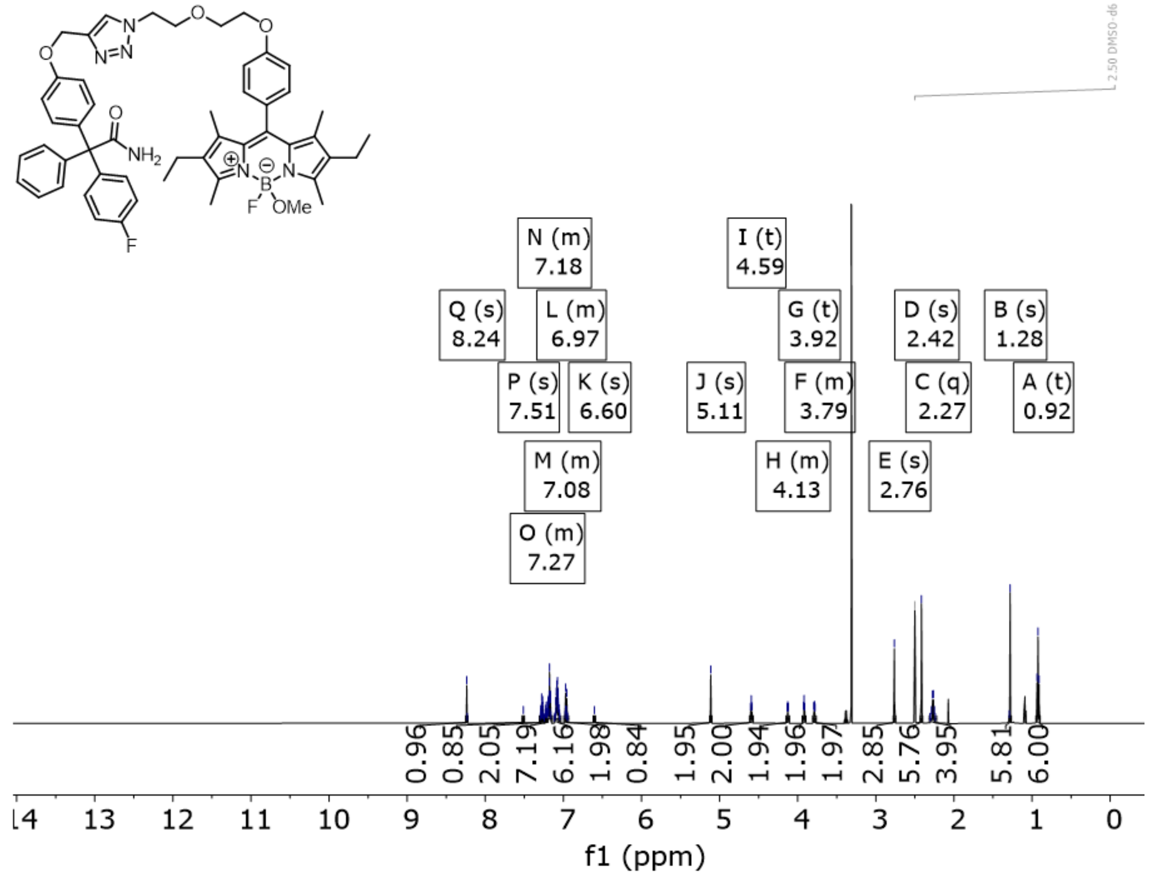

^{13}C NMR spectrum of epoxide **10b** in $CDCl_3$

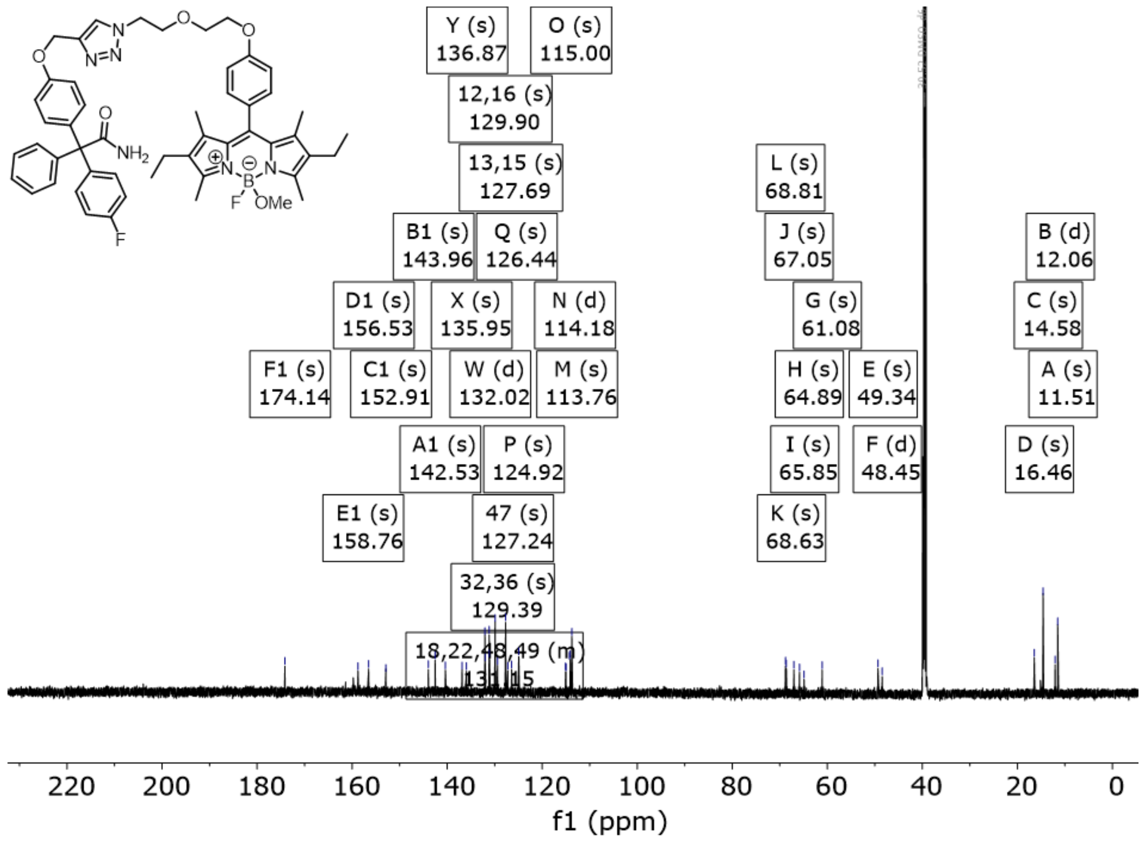

^{1}H NMR spectrum of epoxide **12a** in $CDCl_3$

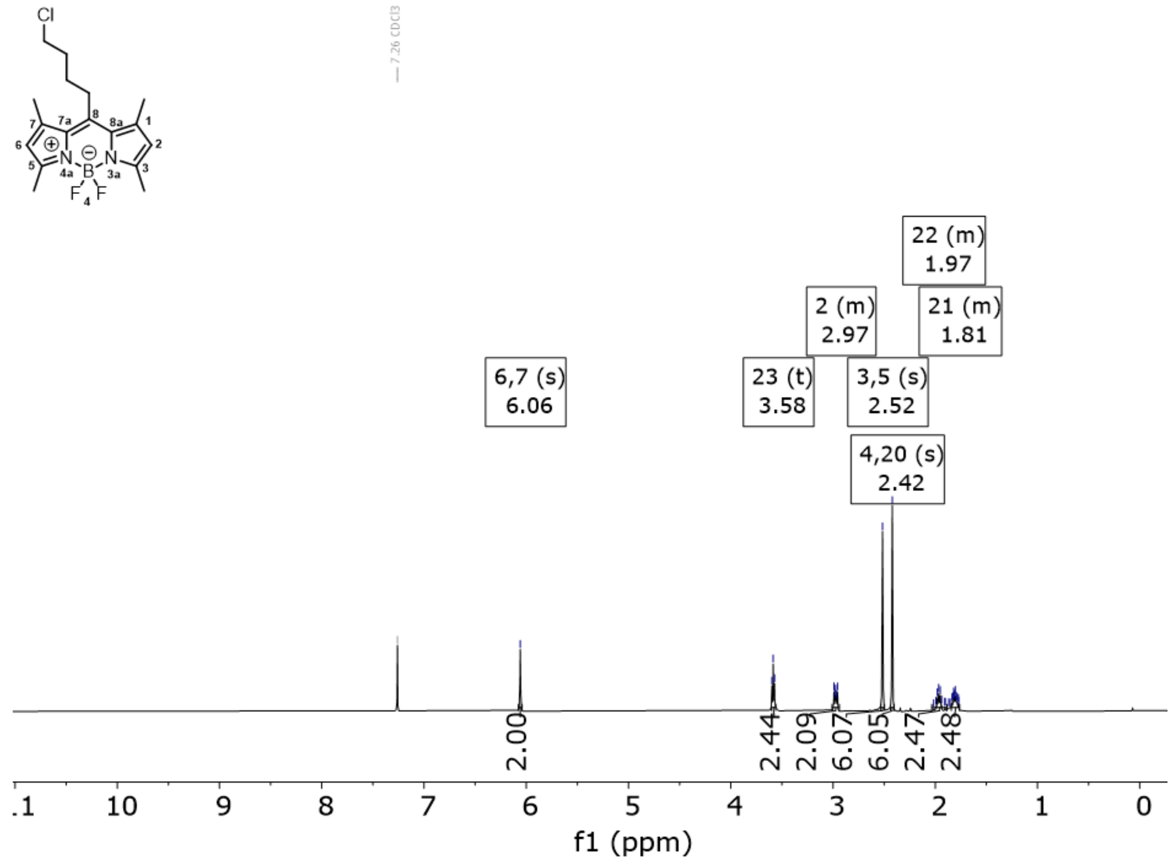

^{13}C NMR spectrum of epoxide **12a** in $CDCl_3$

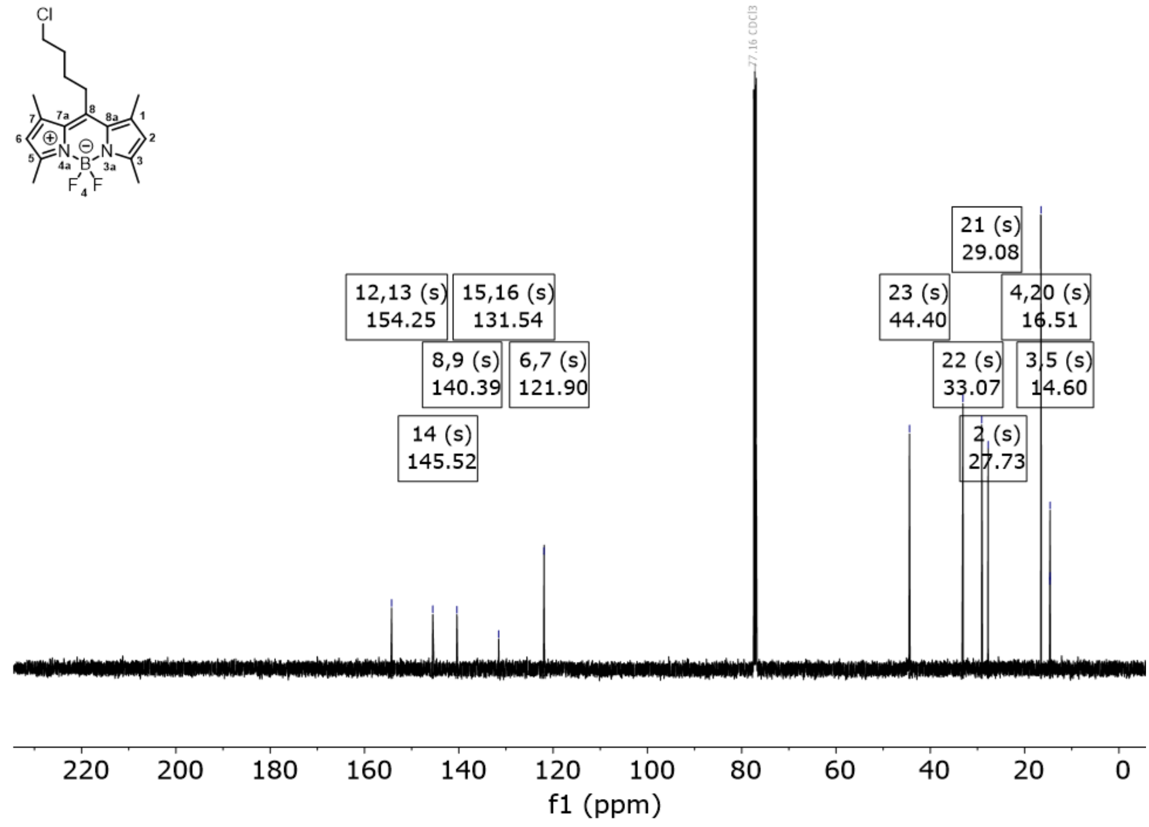

^{1}H NMR spectrum of epoxide **12b** in $CDCl_3$

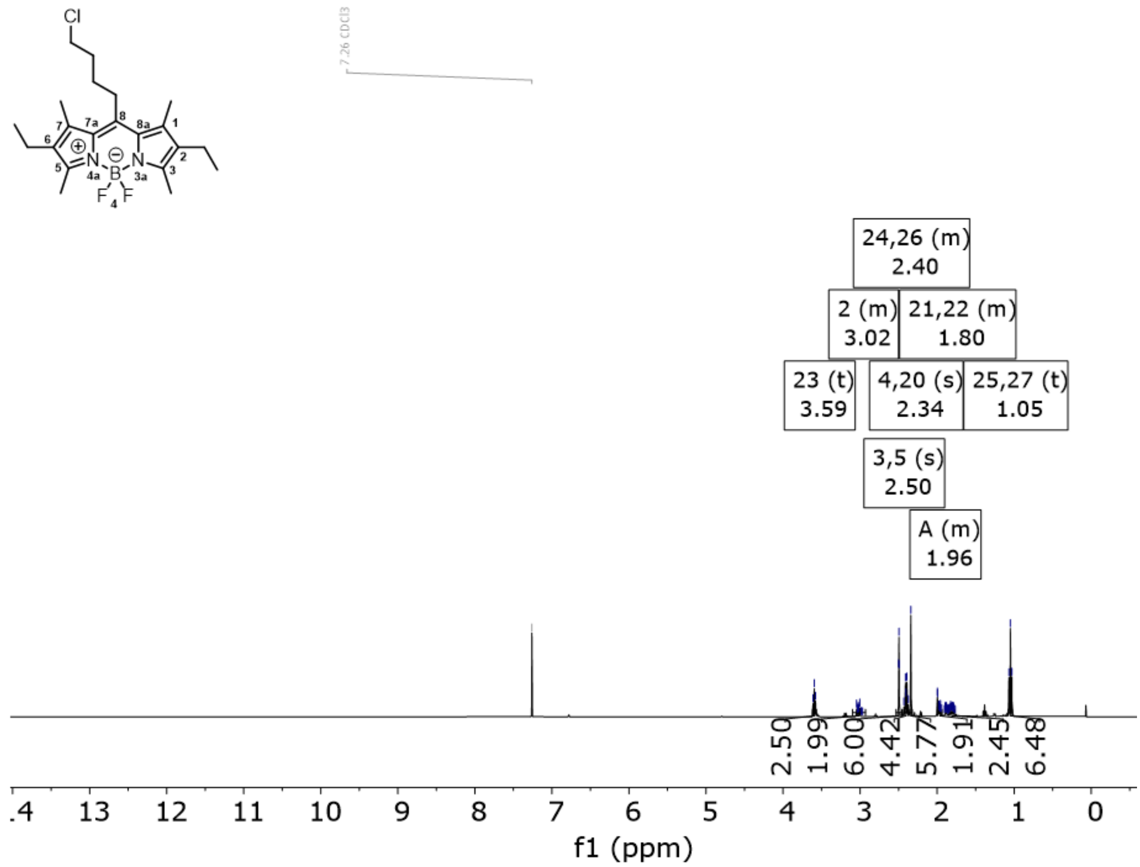

^{13}C NMR spectrum of epoxide **12b** in $CDCl_3$

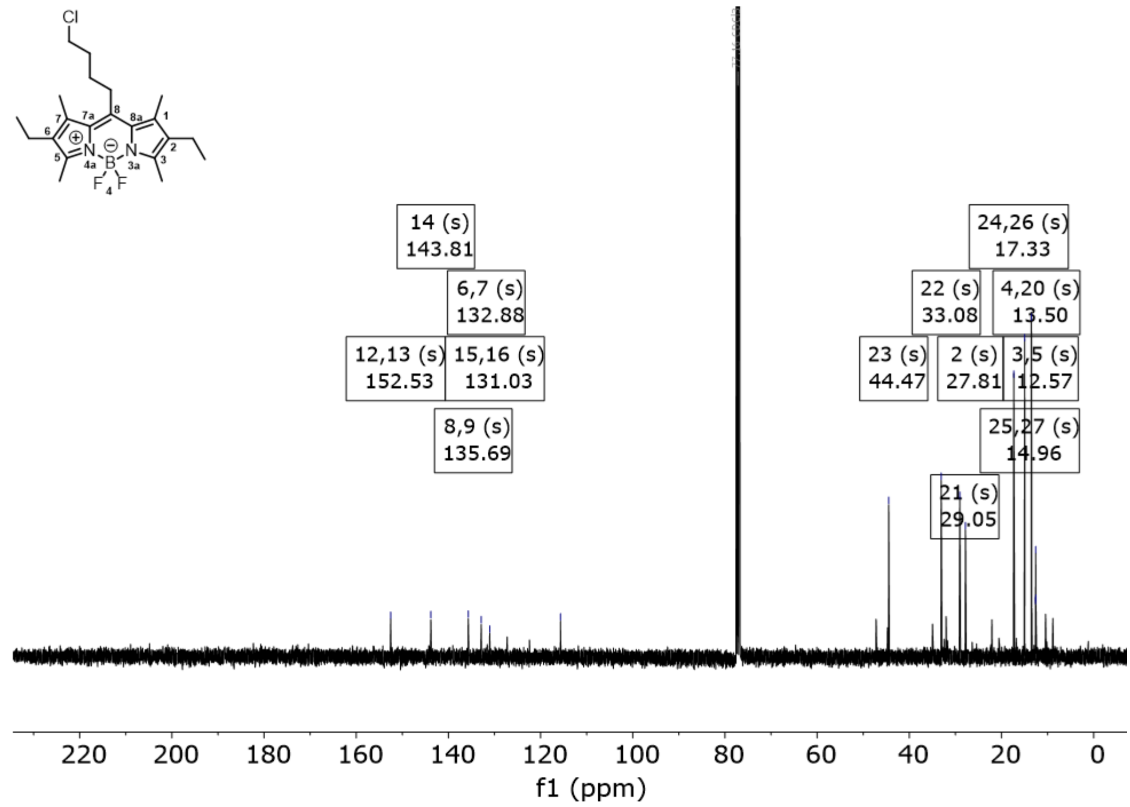

^{1}H NMR spectrum of epoxide **13a** in $CDCl_3$

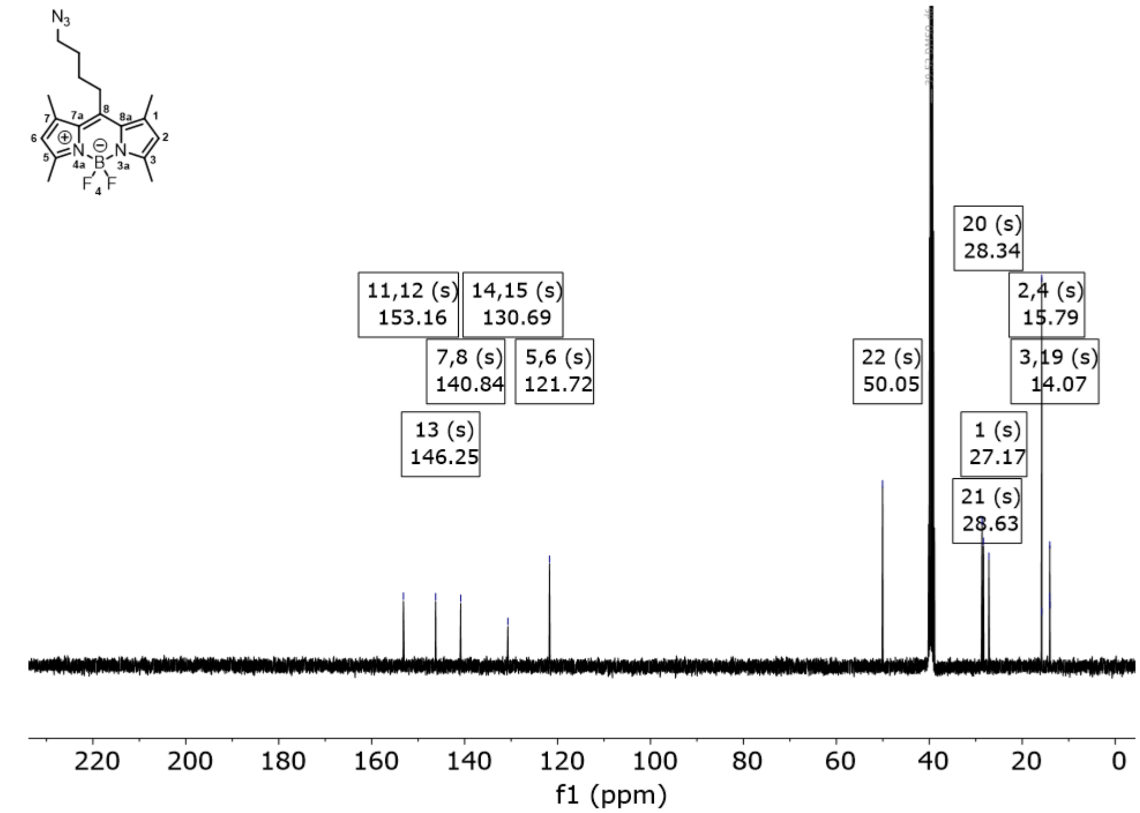

^{13}C NMR spectrum of epoxide **13a** in $CDCl_3$

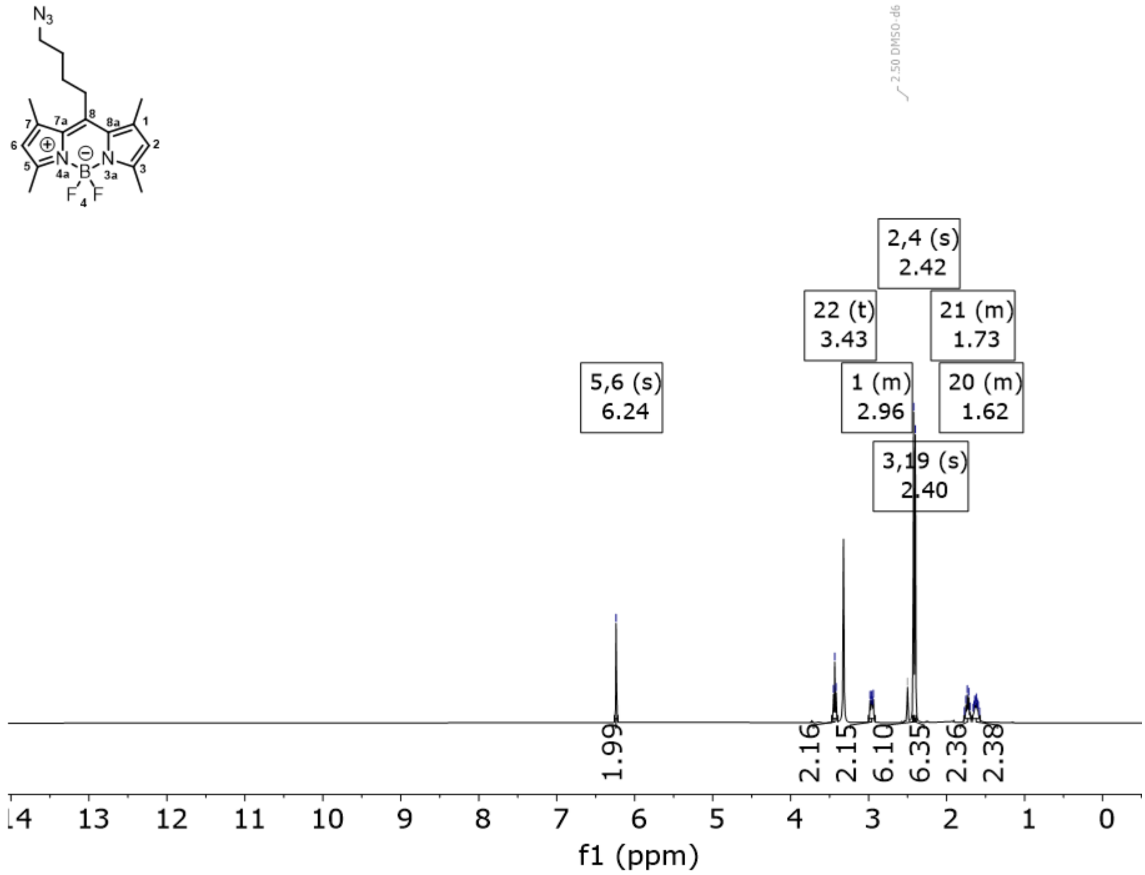

^{1}H NMR spectrum of epoxide **13b** in $CDCl_3$

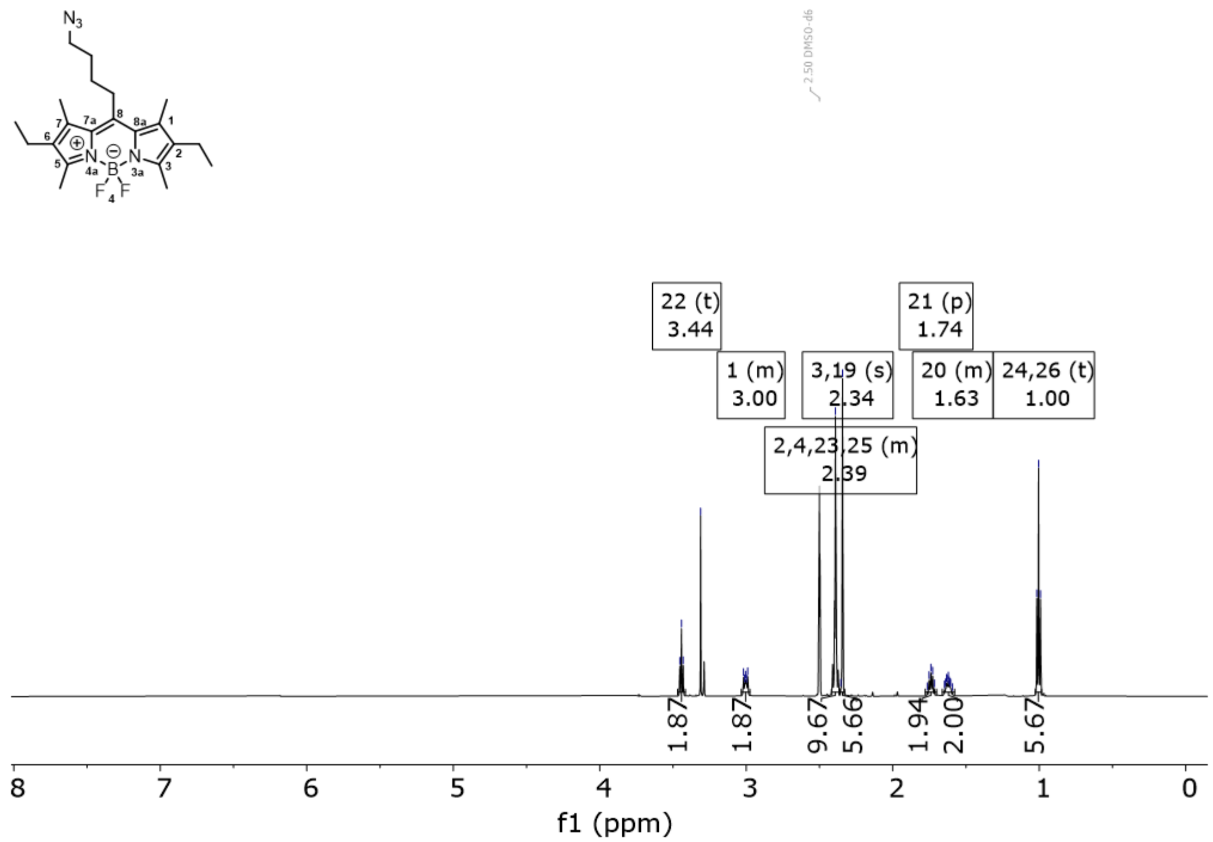

^{13}C NMR spectrum of epoxide **13b** in $CDCl_3$

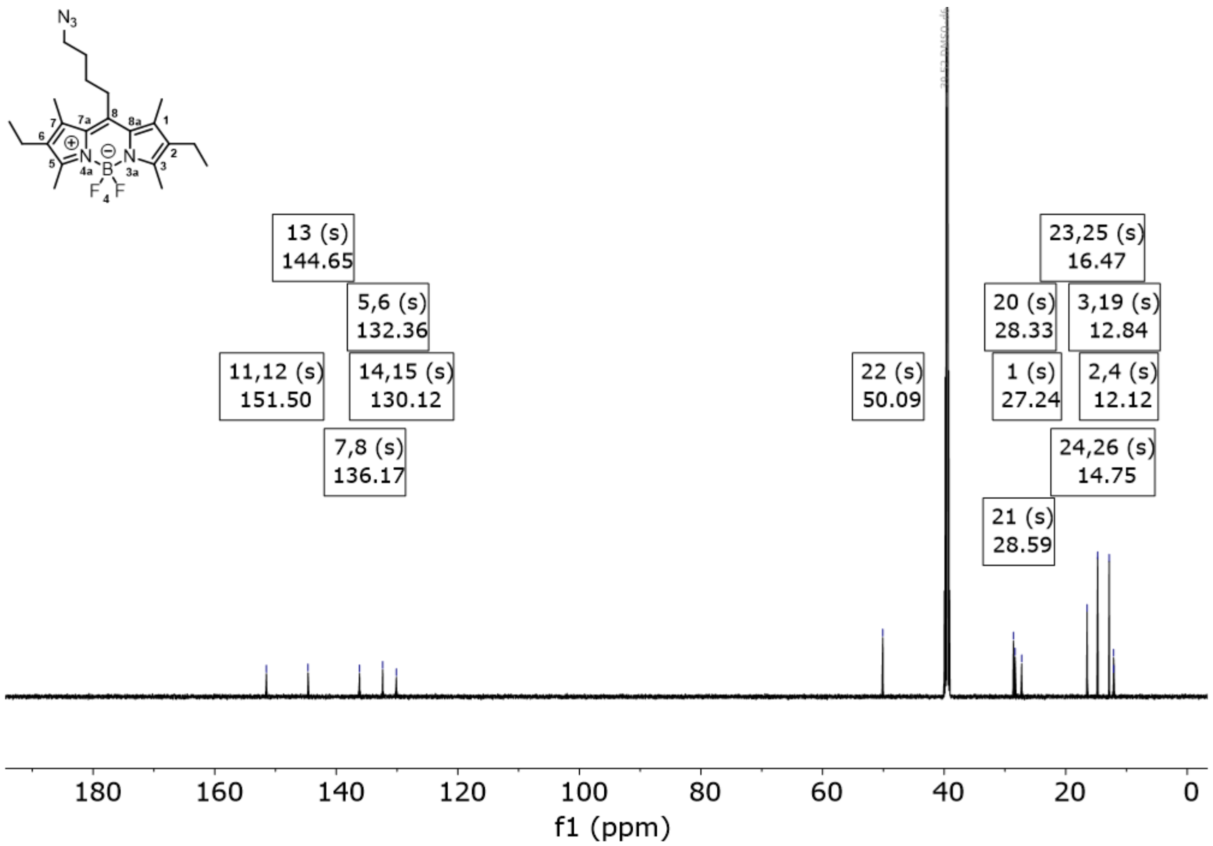

^{1}H NMR spectrum of epoxide **14a** in $CDCl_3$

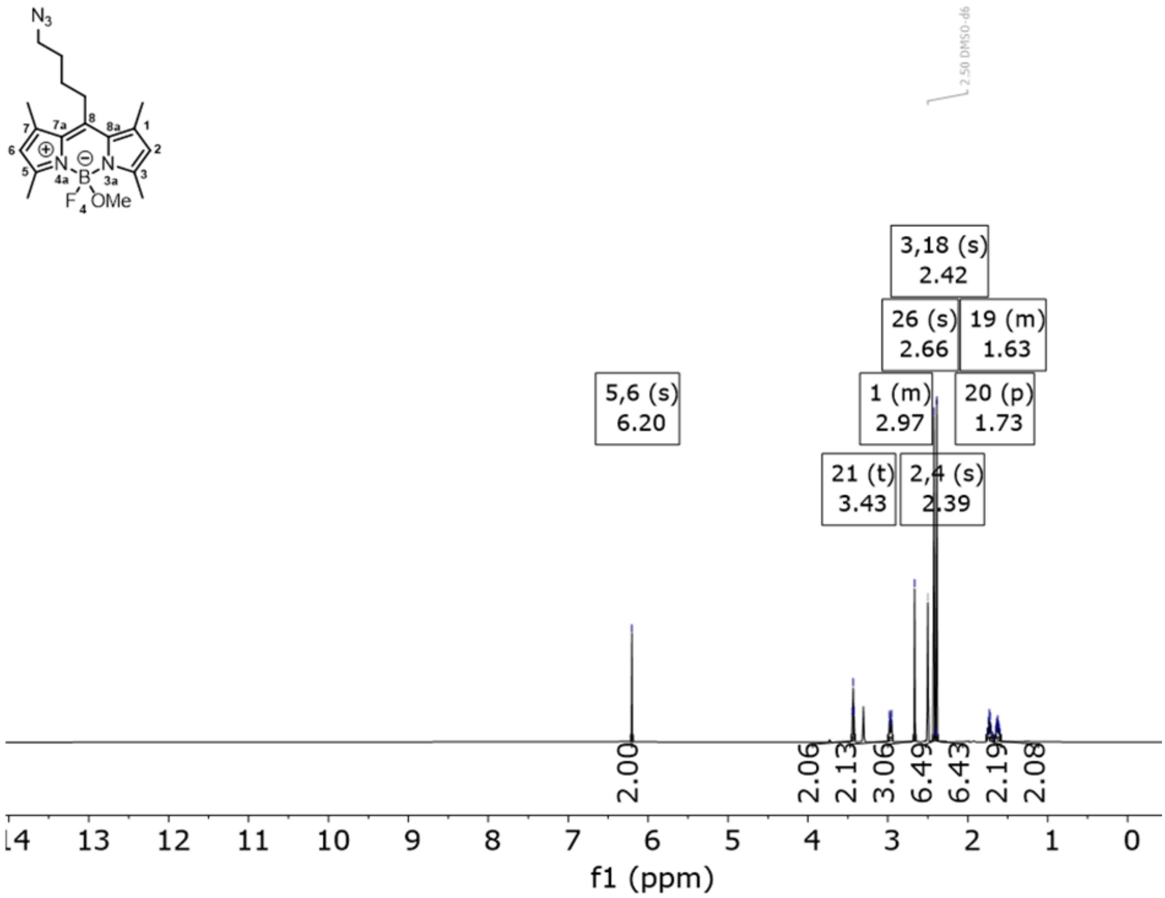

^{13}C NMR spectrum of epoxide **14a** in $CDCl_3$

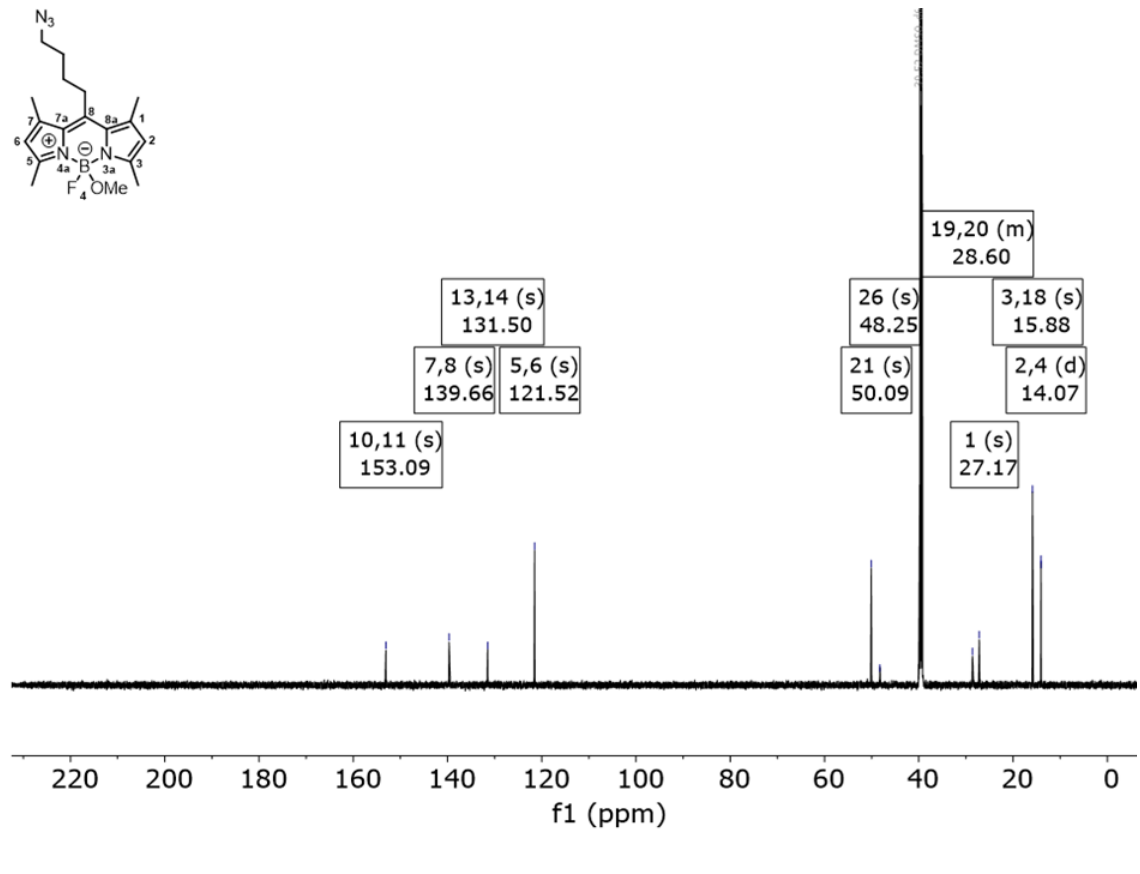

^{1}H NMR spectrum of epoxide **14b** in $CDCl_3$

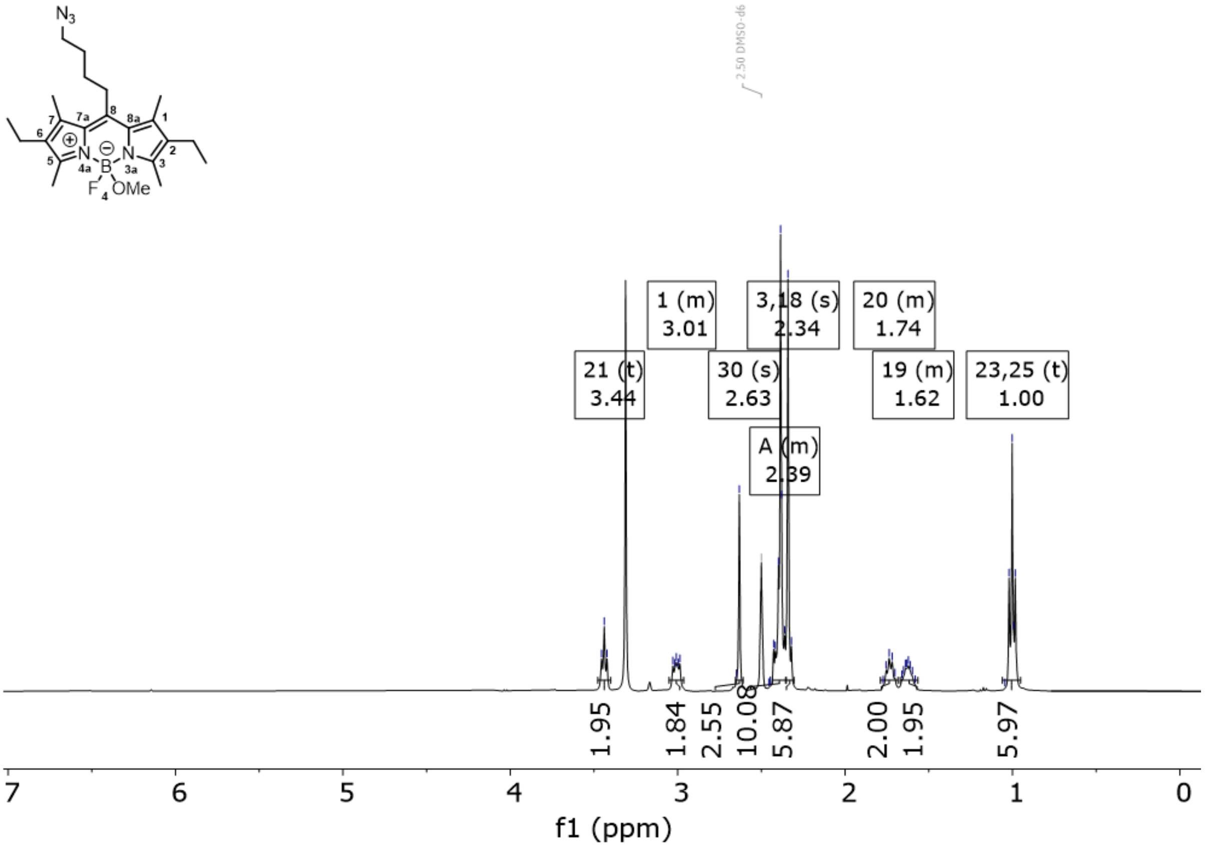

^{13}C NMR spectrum of epoxide **14b** in $CDCl_3$

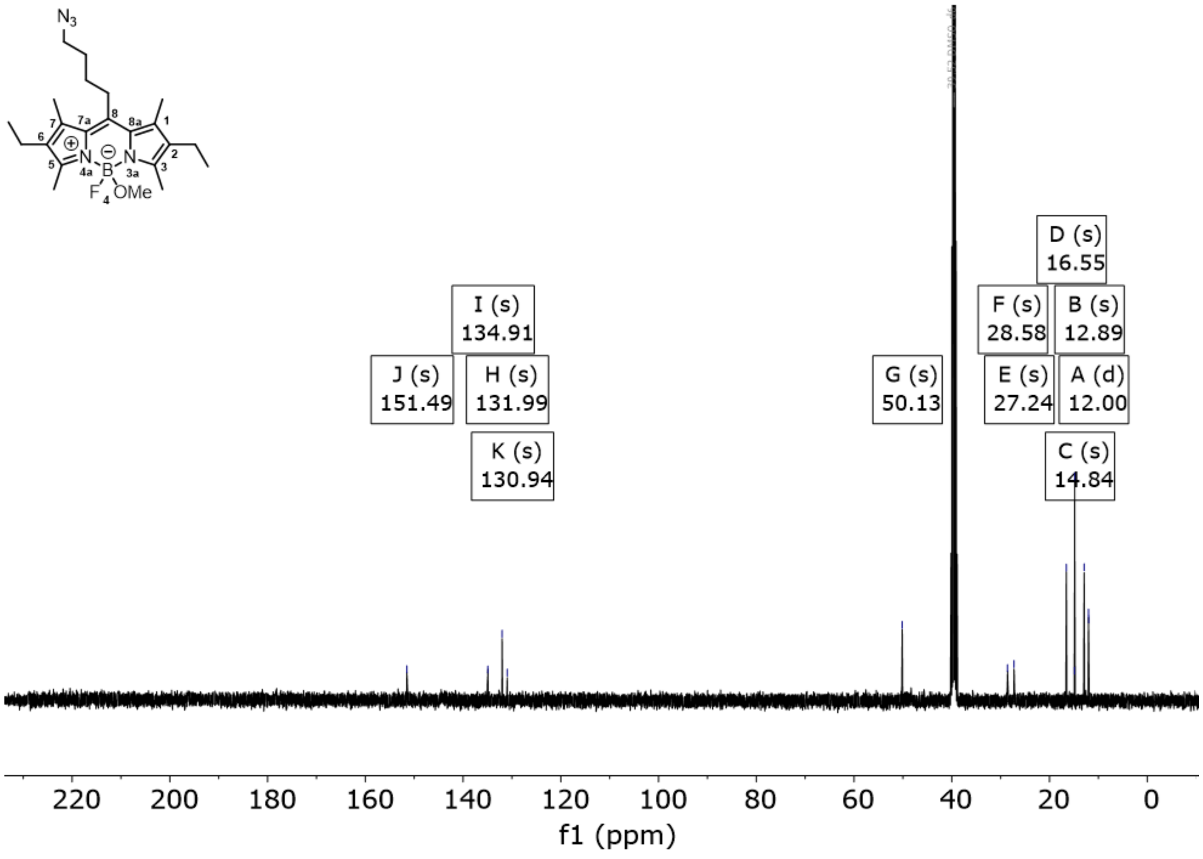

^{1}H NMR spectrum of epoxide **15a** in $CDCl_3$

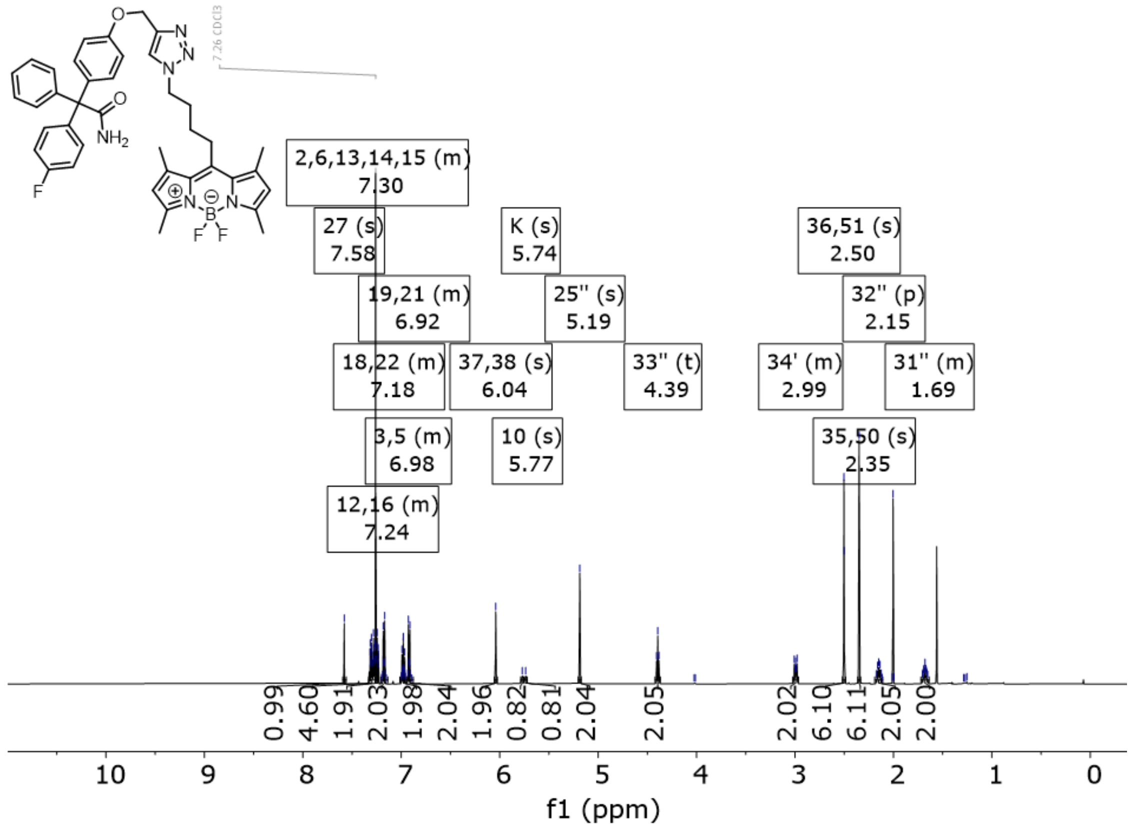

^{13}C NMR spectrum of epoxide **15a** in $CDCl_3$

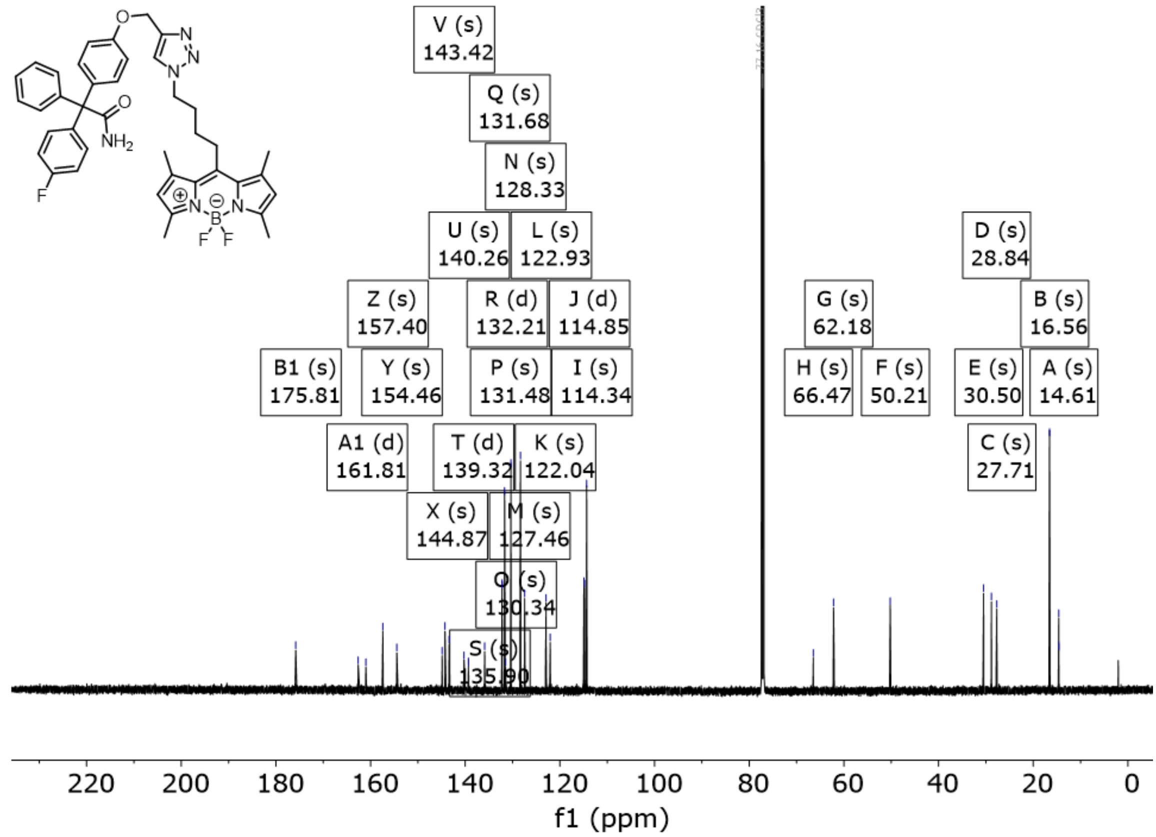

^{1}H NMR spectrum of epoxide **16a** in $CDCl_3$

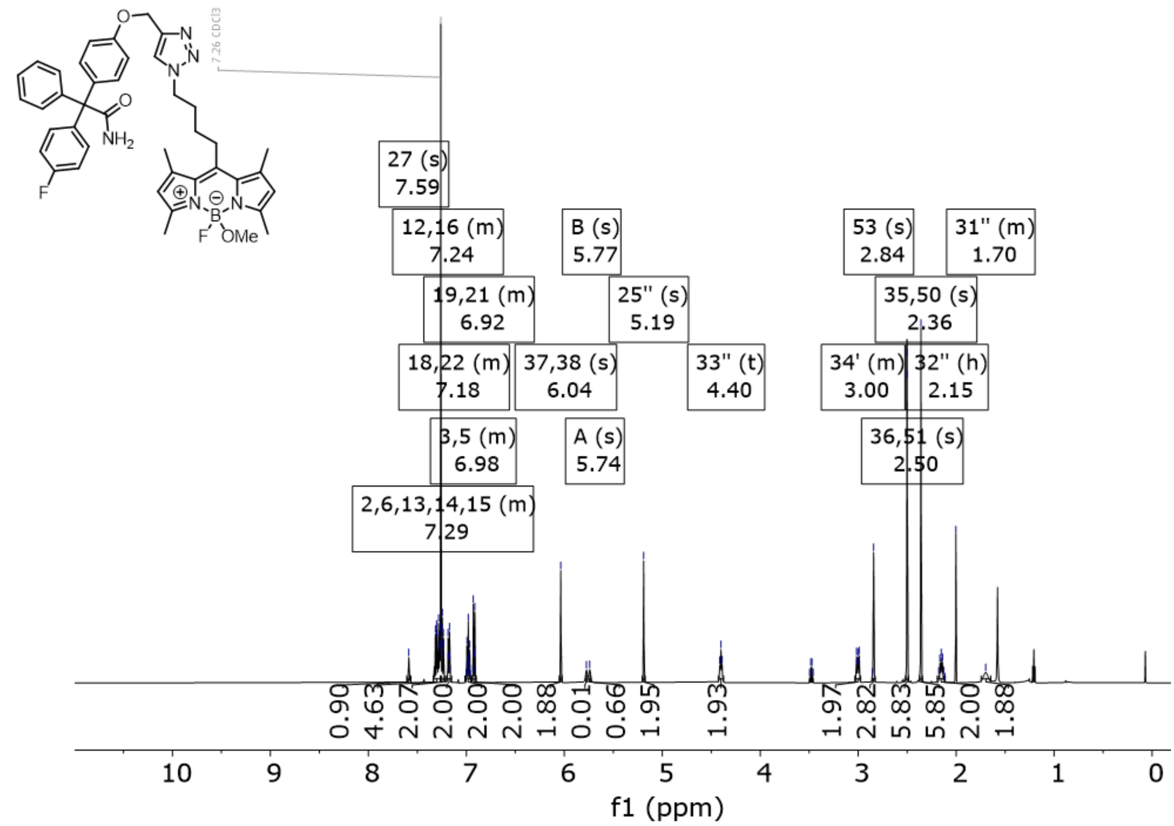

^{13}C NMR spectrum of epoxide **16a** in $CDCl_3$

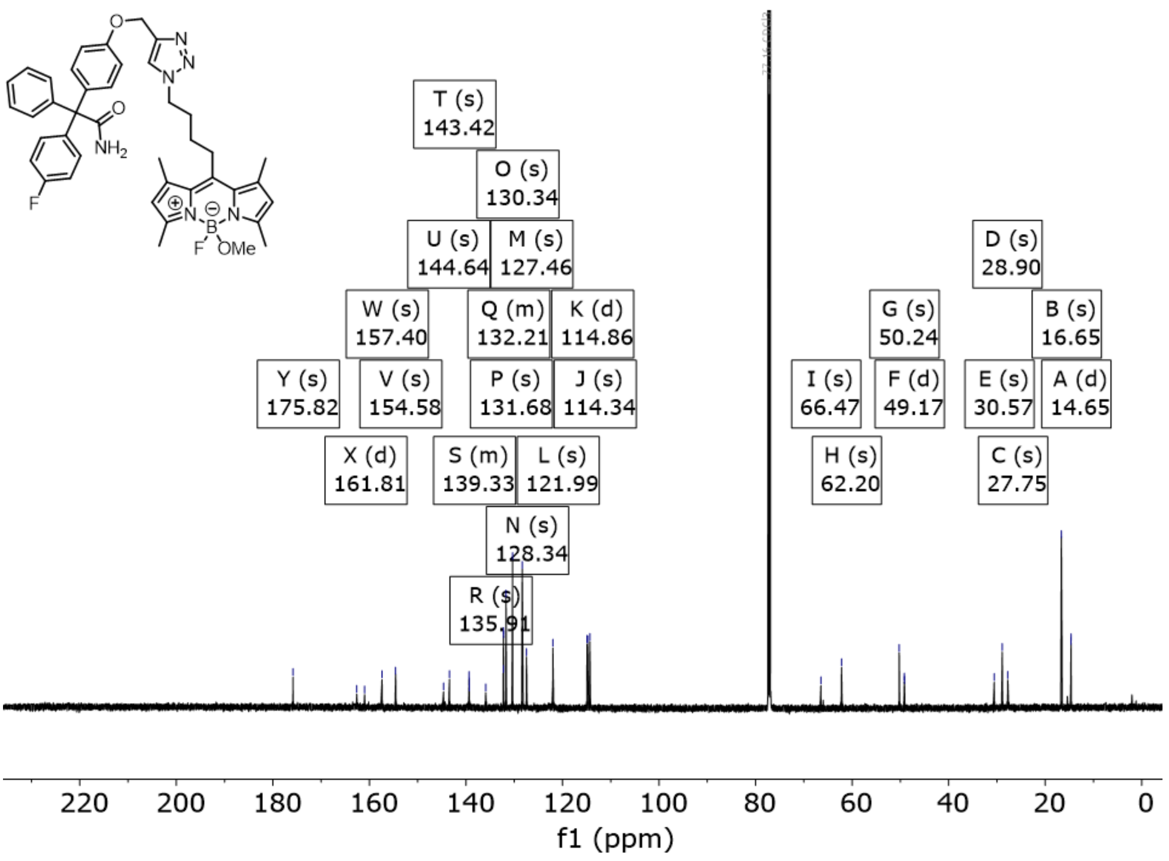

^{1}H NMR spectrum of epoxide **16b** in $CDCl_3$

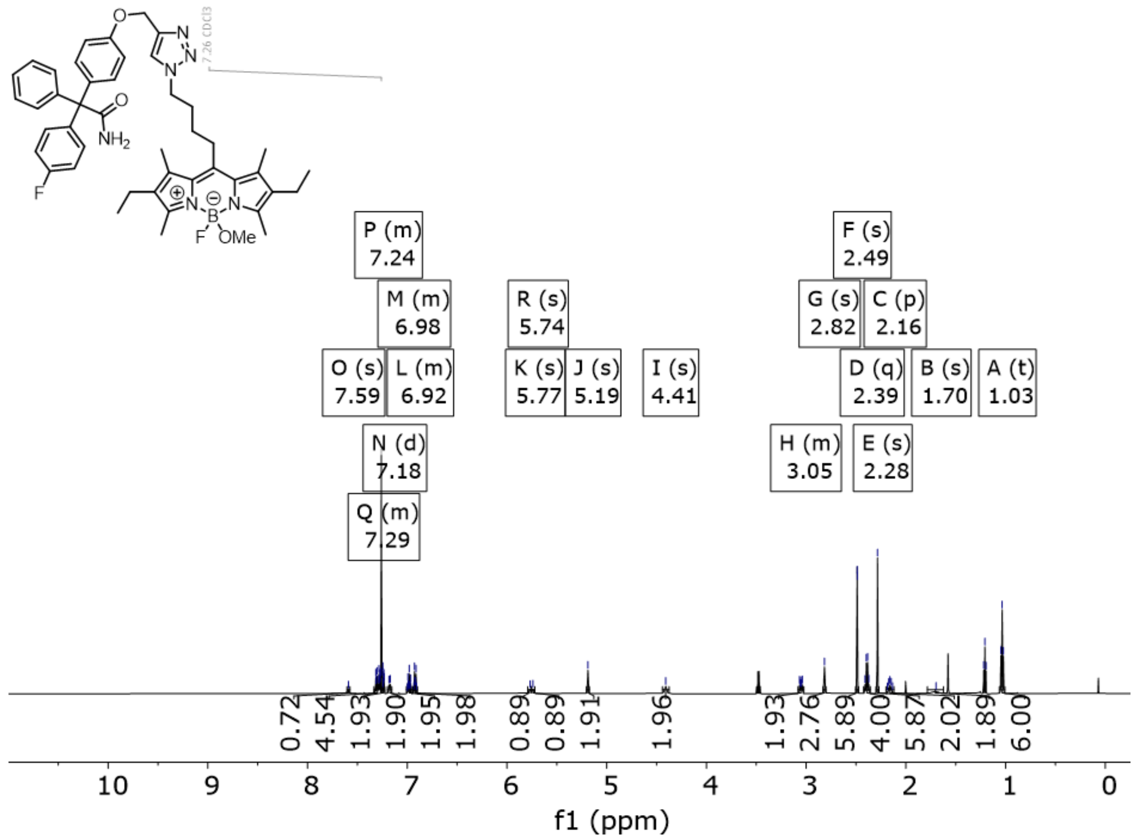

^{13}C NMR spectrum of epoxide **16b** in $CDCl_3$

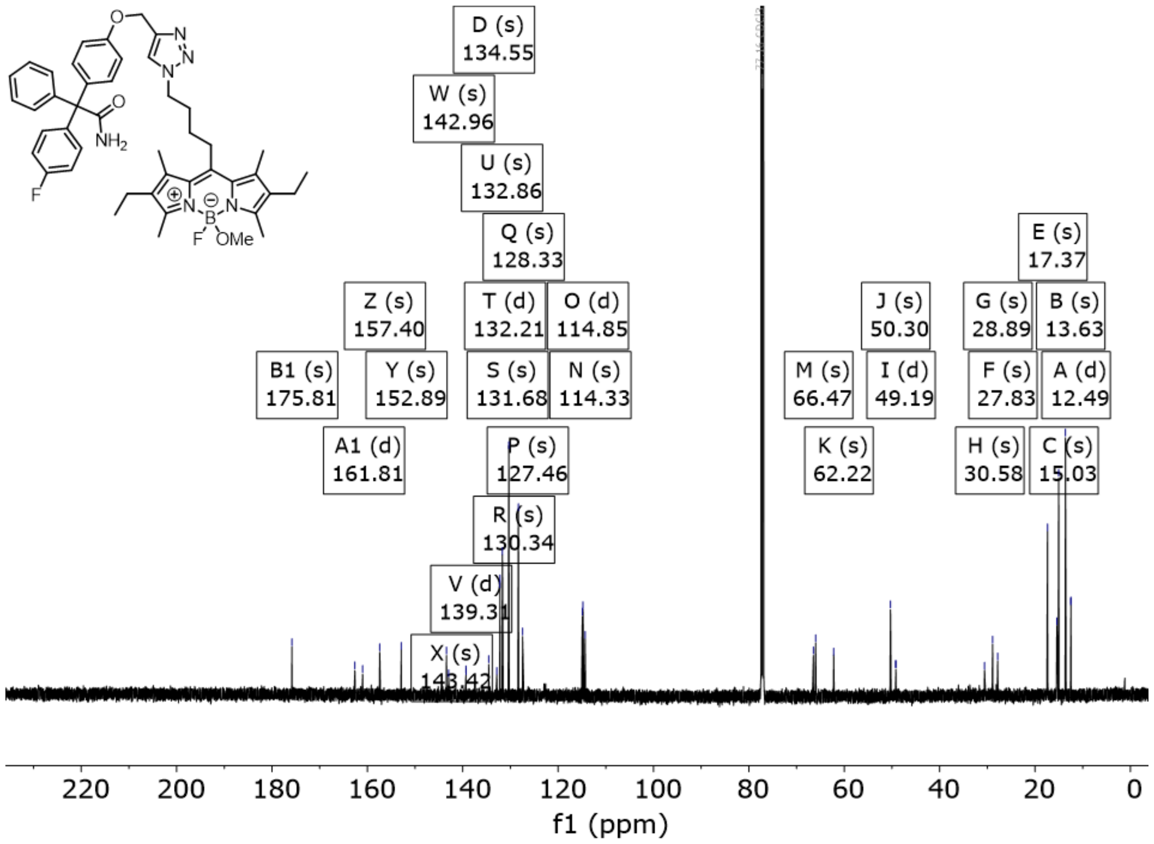

^{1}H NMR spectrum of epoxide **18a** in $CDCl_3$

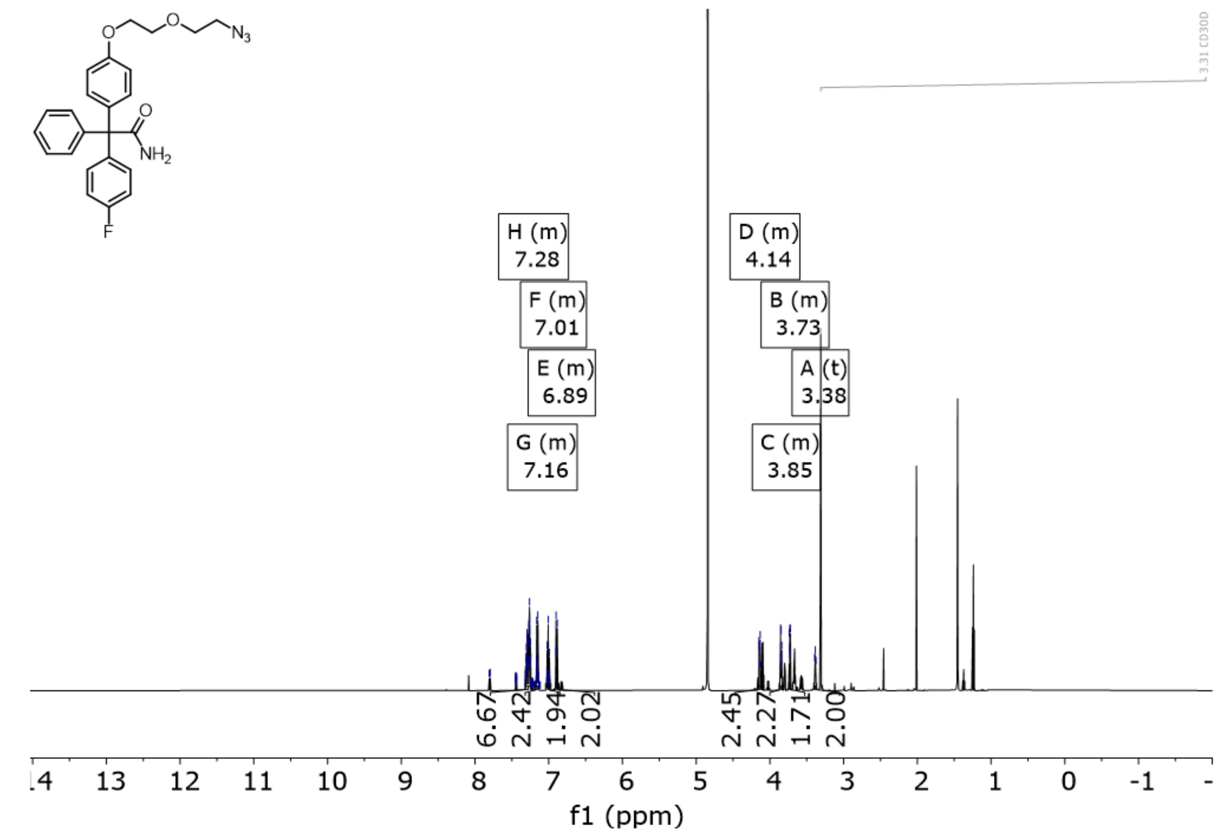

^{13}C NMR spectrum of epoxide **18a** in $CDCl_3$

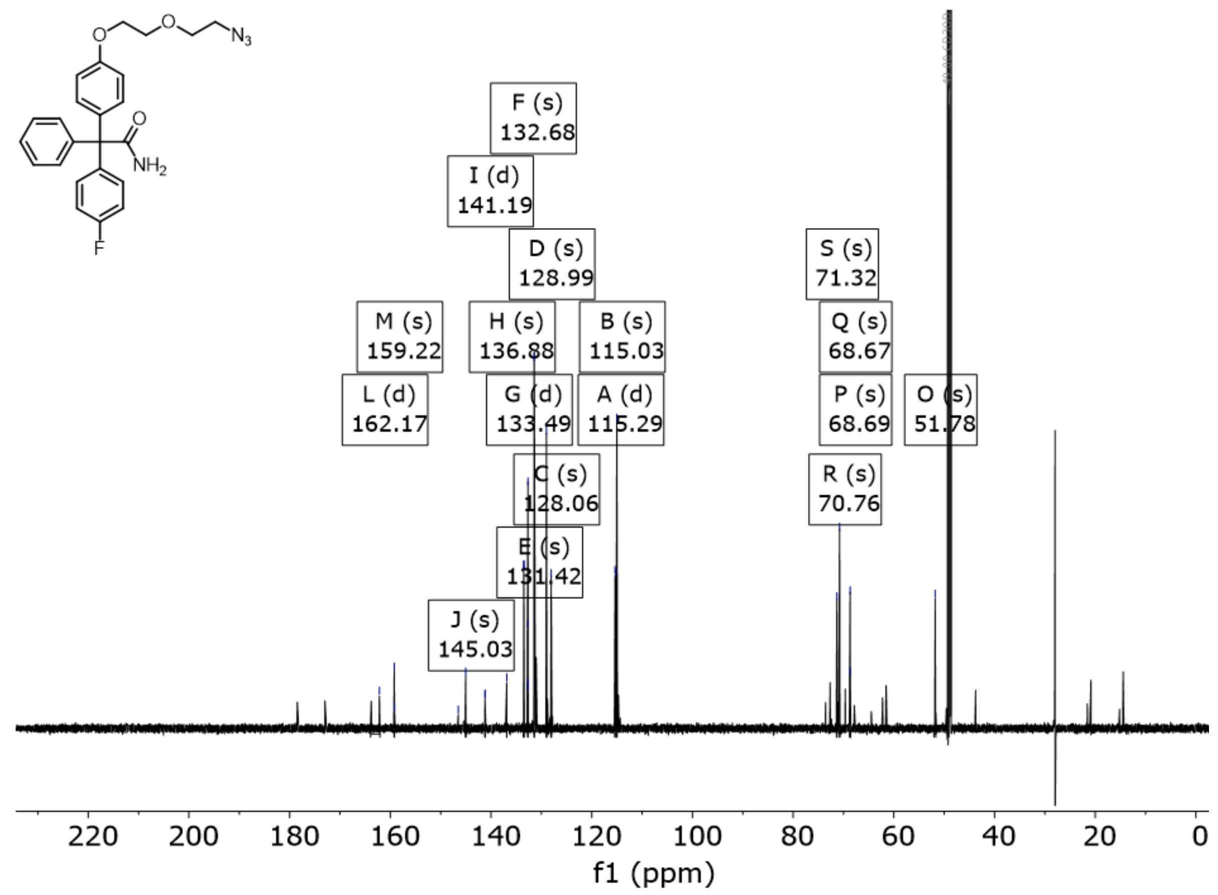

^{1}H NMR spectrum of epoxide **19b** in $CDCl_3$

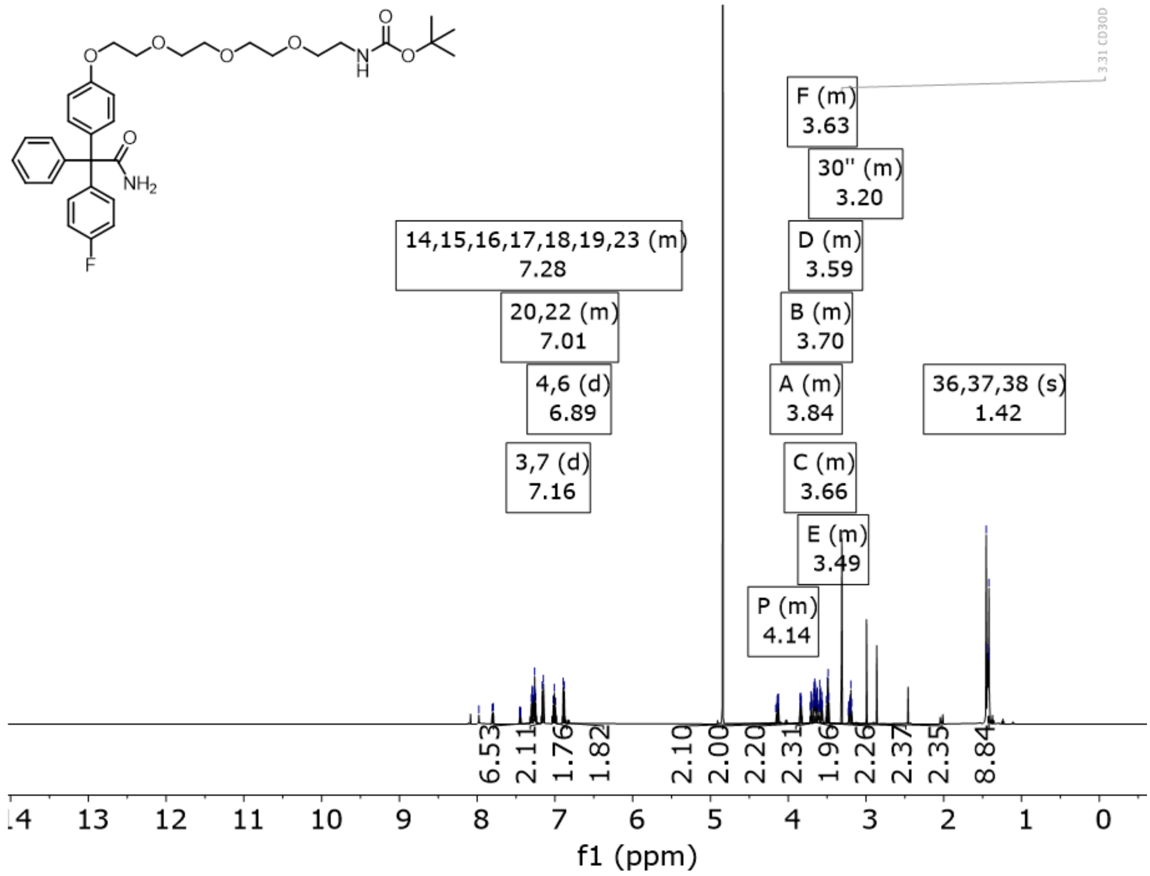

^{13}C NMR spectrum of epoxide **19b** in $CDCl_3$

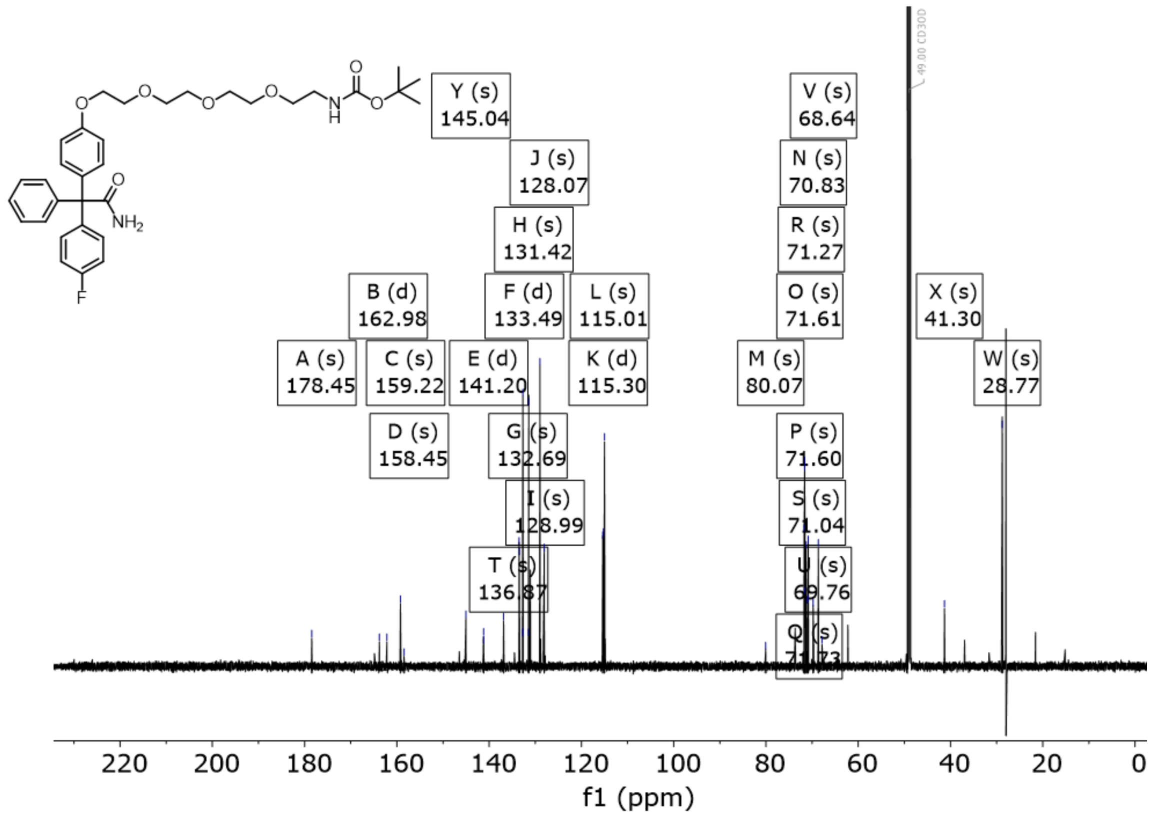

^{1}H NMR spectrum of epoxide **21a** in $CDCl_3$

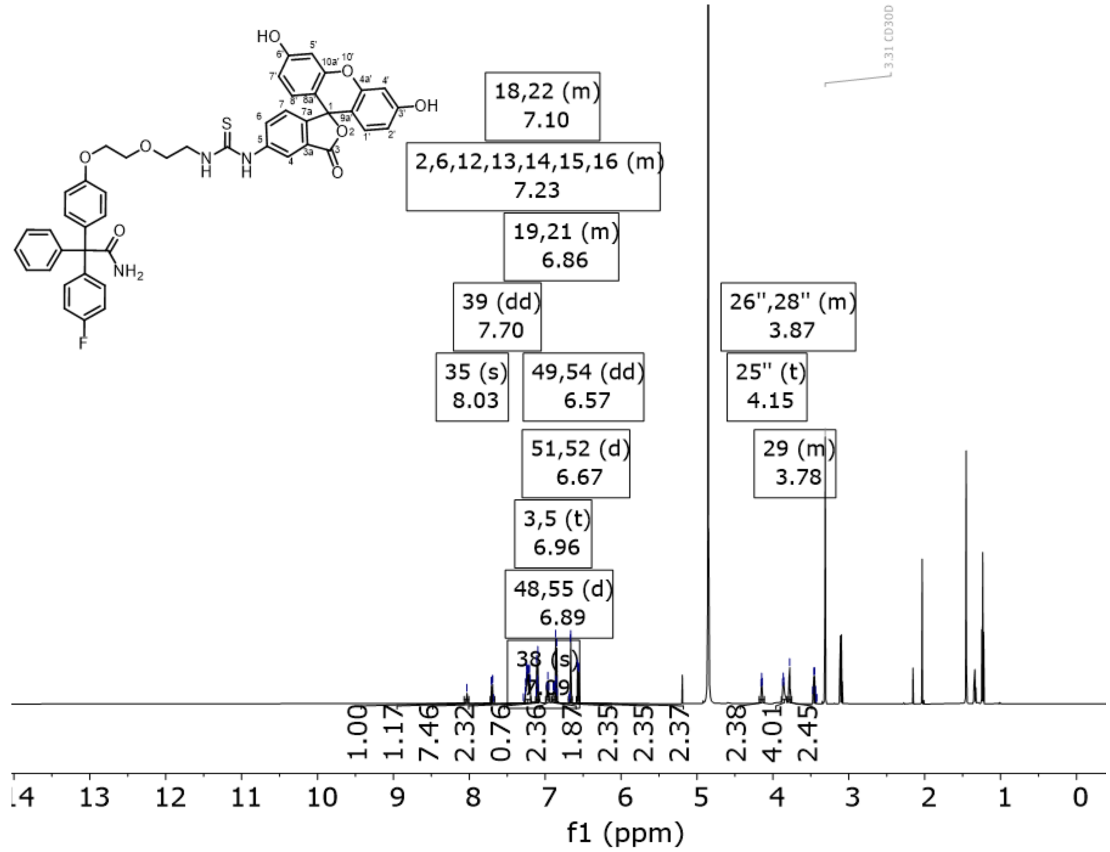

^{13}C NMR spectrum of epoxide **21a** in $CDCl_3$

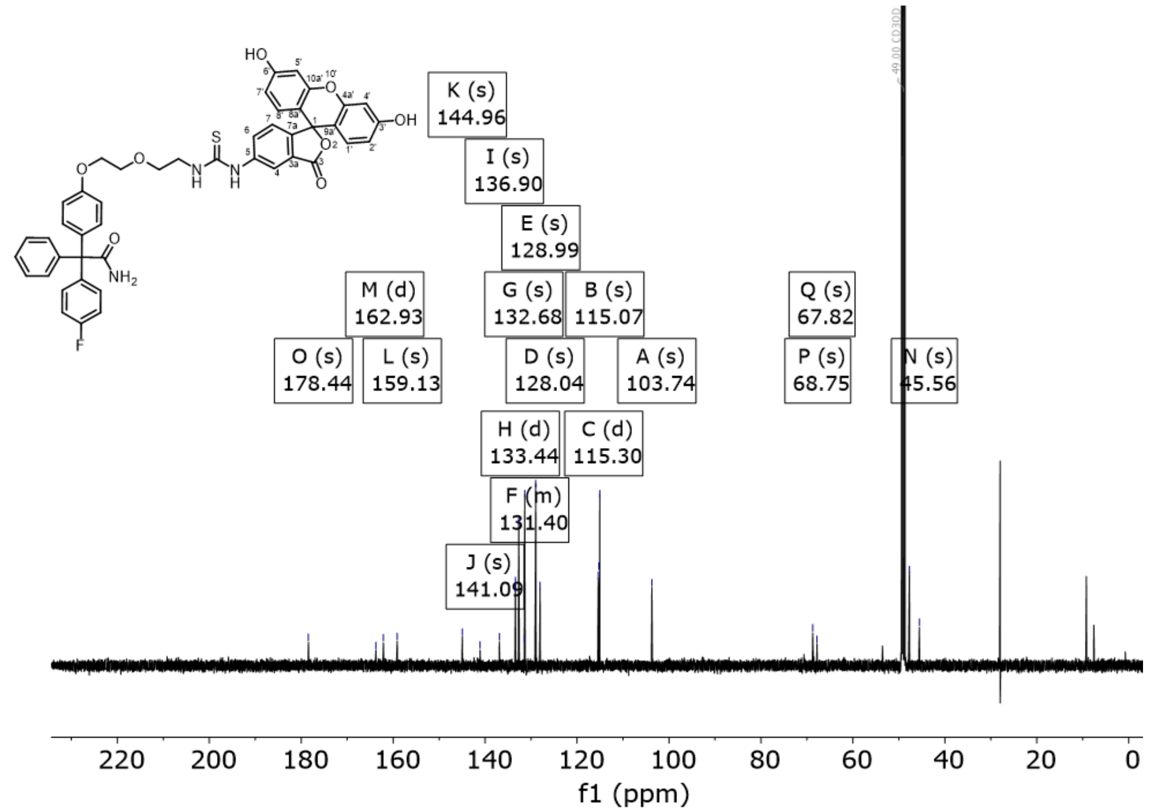

^{1}H NMR spectrum of epoxide **21b** in $CDCl_3$

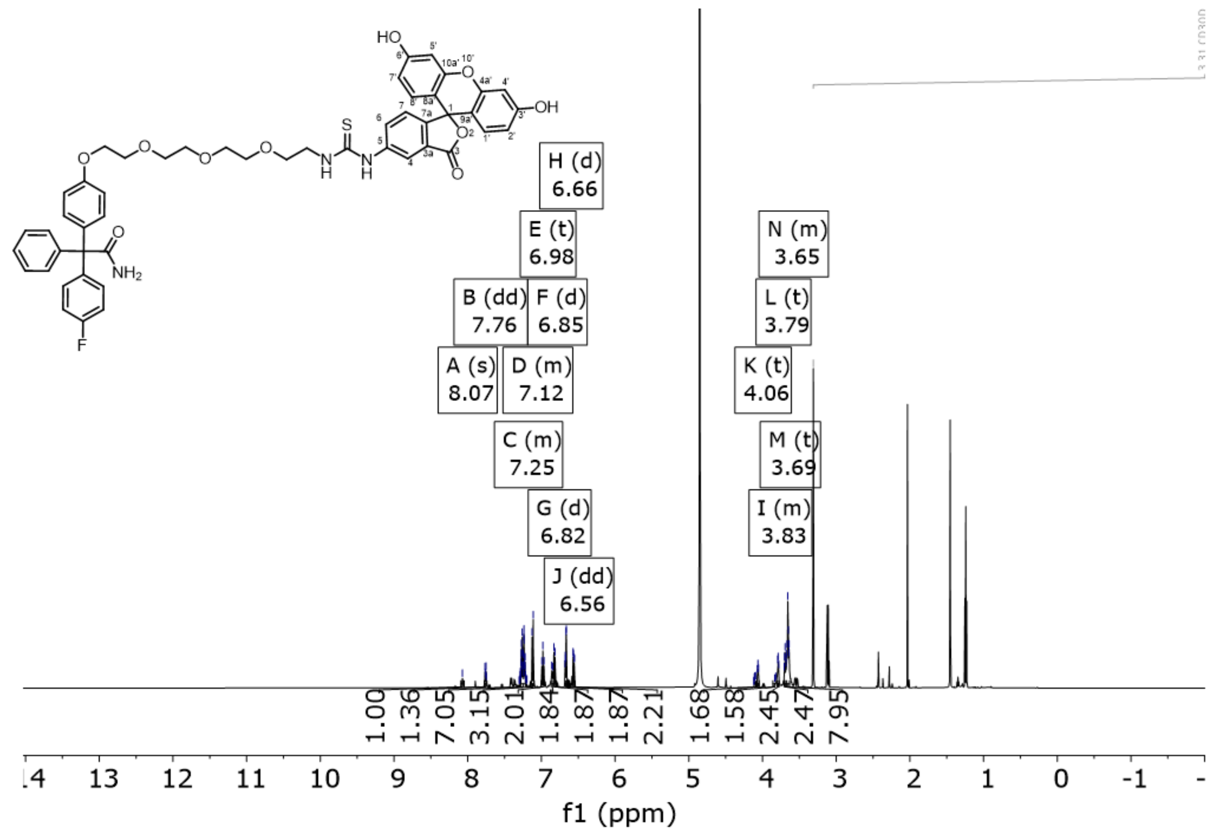

^{13}C NMR spectrum of epoxide **21b** in $CDCl_3$

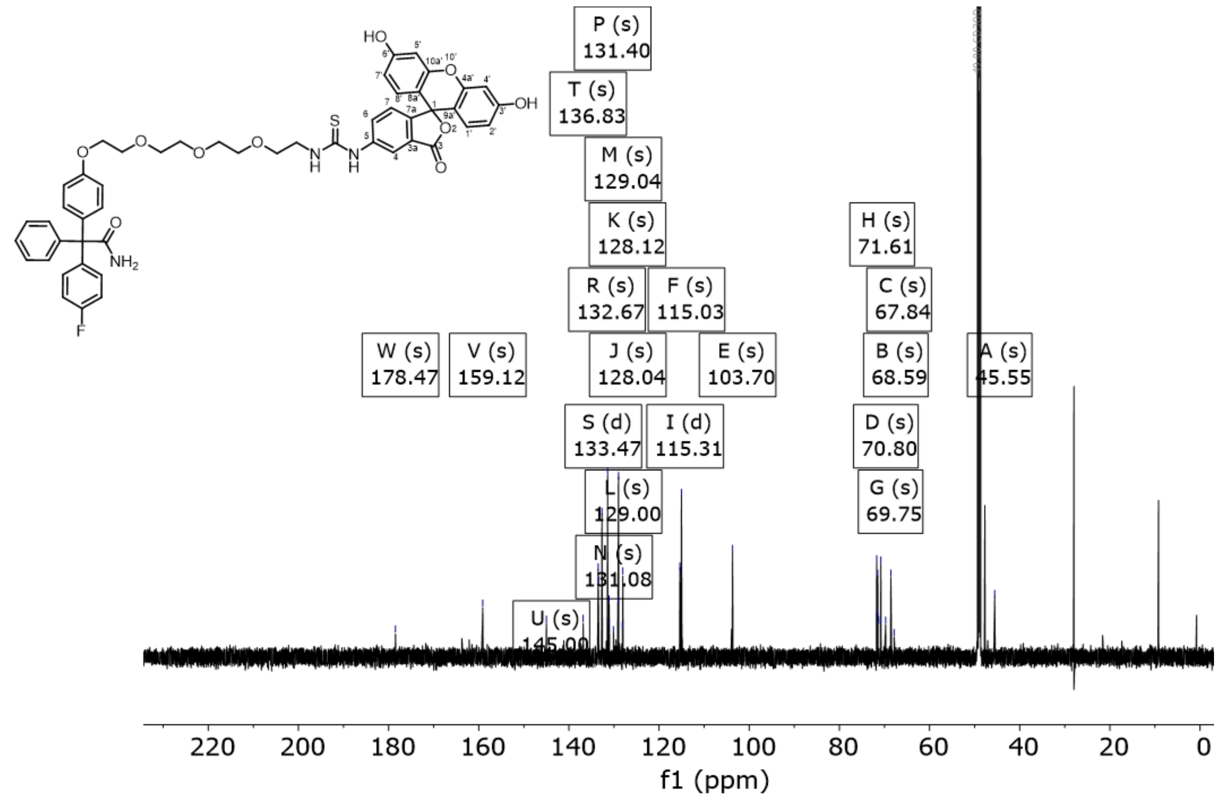

12. HPLC chromatograms

HPLC analysis of **6b**

11 BP02

Sample Name:	BP02	*Injection Volume:*	5,0
Vial Number:	RB5	*Channel:*	UV_VIS_1
Sample Type:	unknown	*Wavelength:*	210
Quantif. Method:	Bioanalytik	*Dilution Factor:*	1,0000
Recording Time:		*Sample Weight:*	1,0000
Run Time (min):	30,00	*Sample Amount:*	1,0000

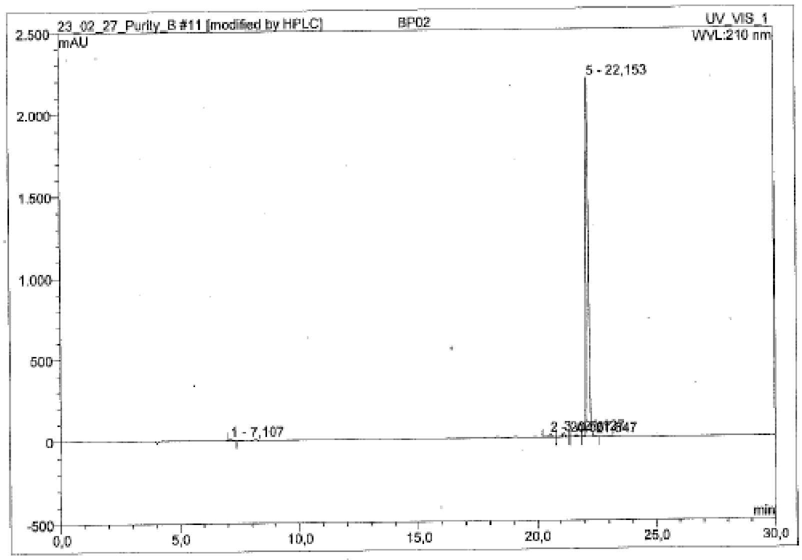

No.	Ret.Time min	Peak Name	Height mAU	Area mAU*min	Rel.Area %	Amount	Type
1	7,11	n.a.	9,848	1,165	0,41	n.a.	BMB*
2	20,57	n.a.	17,888	2,299	0,82	n.a.	BM *
3	21,13	n.a.	24,588	3,056	1,09	n.a.	Mb*
4	21,65	n.a.	10,824	1,473	0,52	n.a.	bM *
5	22,15	n.a.	2195,112	273,148	97,16	n.a.	MB*
Total:			2258,261	281,141	100,00	0,000	

HPLC analysis of **7b**

9 HPLC BP38

Sample Name:	**HPLC BP38**	*Injection Volume:*	**5,0**
Vial Number:	**RB4**	*Channel:*	**UV_VIS_1**
Sample Type:	**unknown**	*Wavelength:*	**210**
Quantif. Method:	**Bioanalytik**	*Dilution Factor:*	**1,0000**
Recording Time:		*Sample Weight:*	**1,0000**
Run Time (min):	**30,00**	*Sample Amount:*	**1,0000**

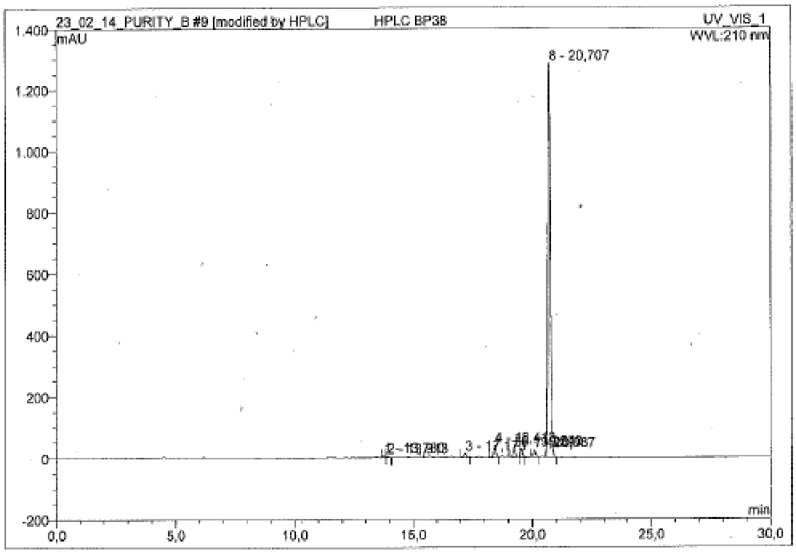

No.	Ret.Time min	Peak Name	Height mAU	Area mAU*min	Rel.Area %	Amount	Type
1	13,76	n.a.	5,714	0,582	0,36	n.a.	BM *
2	13,91	n.a.	4,037	0,467	0,29	n.a.	MB*
3	17,17	n.a.	12,394	1,473	0,90	n.a.	BMB*
4	18,41	n.a.	38,858	4,170	2,56	n.a.	BMB*
5	19,23	n.a.	25,443	2,814	1,73	n.a.	BM *
6	19,54	n.a.	27,853	3,027	1,86	n.a.	MB*
7	20,09	n.a.	23,095	2,588	1,59	n.a.	BMB*
8	20,71	n.a.	1284,380	147,762	90,72	n.a.	BMB
**Total:**			1421,773	162,883	100,00	0,000	

HPLC analysis of **9b**

22 IT24

Sample Name:	**IT24**	*Injection Volume:*	**5,0**
Vial Number:	**RC1**	*Channel:*	**UV_VIS_1**
Sample Type:	**unknown**	*Wavelength:*	**210**
Quantif. Method:	**Bioanalytik**	*Dilution Factor:*	**1,0000**
Recording Time:		*Sample Weight:*	**1,0000**
Run Time (min):	**30,00**	*Sample Amount:*	**1,0000**

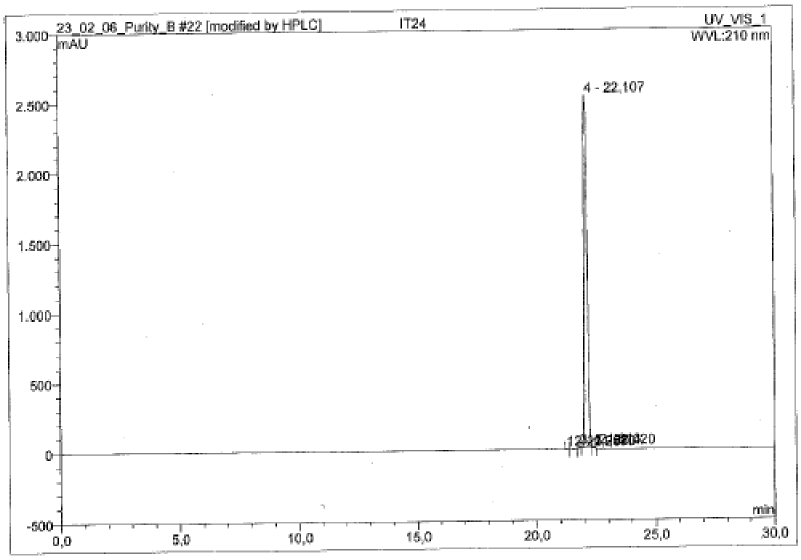

No.	Ret.Time min	Peak Name	Height mAU	Area mAU*min	Rel.Area %	Amount	Type
1	21,27	n.a.	3,681	0,397	0,11	n.a.	BMb*
2	21,58	n.a.	3,911	0,457	0,13	n.a.	bMB*
3	21,81	n.a.	10,379	1,096	0,31	n.a.	BMB*
4	22,11	n.a.	2517,349	353,482	99,15	n.a.	BM *
5	22,42	n.a.	10,666	1,090	0,31	n.a.	MB*
**Total:**			2546,186	356,522	100,00	0,000	

HPLC analysis of **10b** **in der Datei von Insa zwei Chromatogramme zu Nr. 56?!**

20 IT26

Sample Name:	**IT26**	*Injection Volume:*	**5,0**
Vial Number:	**RB8**	*Channel:*	**UV_VIS_1**
Sample Type:	**unknown**	*Wavelength:*	**210**
Quantif. Method:	**Bioanalytik**	*Dilution Factor:*	**1,0000**
Recording Time:		*Sample Weight:*	**1,0000**
Run Time (min):	**30,00**	*Sample Amount:*	**1,0000**

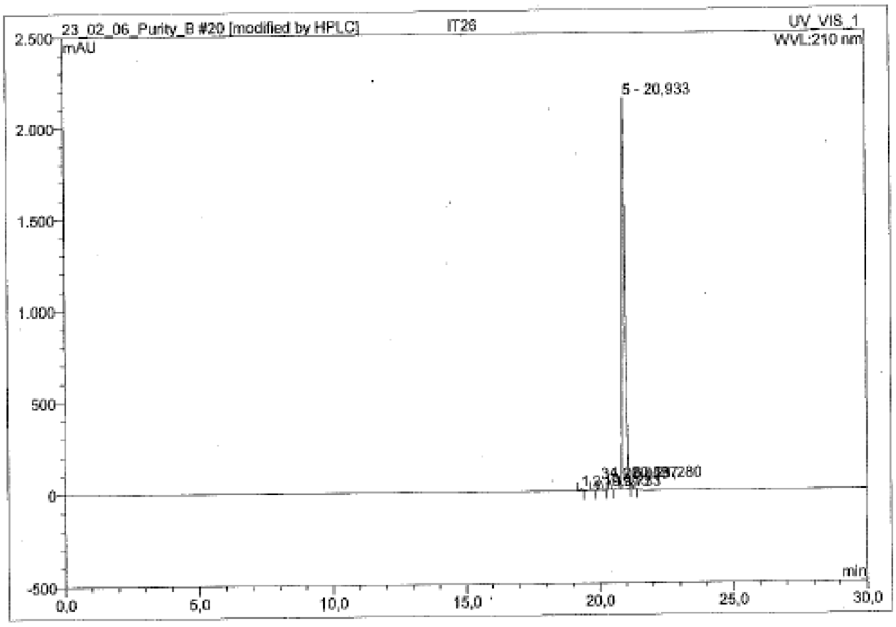

No.	Ret.Time min	Peak Name	Height mAU	Area mAU*min	Rel.Area %	Amount	Type
1	19,31	n.a.	8,130	0,908	0,30	n.a.	BMB*
2	19,73	n.a.	3,432	0,318	0,11	n.a.	BMB*
3	20,05	n.a.	46,489	6,112	2,05	n.a.	BMB*
4	20,39	n.a.	51,024	5,570	1,87	n.a.	BMB*
5	20,93	n.a.	2132,218	280,585	94,00	n.a.	BMb*
6	21,28	n.a.	45,577	5,013	1,68	n.a.	bMB*
Total:			2286,870	298,506	100,00	0,000	

17 IT26

Sample Name:	IT26	*Injection Volume:*	5,0
Vial Number:	BC2	*Channel:*	UV_VIS_1
Sample Type:	unknown	*Wavelength:*	210
Quantif. Method:	Bioanalytik	*Dilution Factor:*	1,0000
Recording Time:		*Sample Weight:*	1,0000
Run Time (min):	30,00	*Sample Amount:*	1,0000

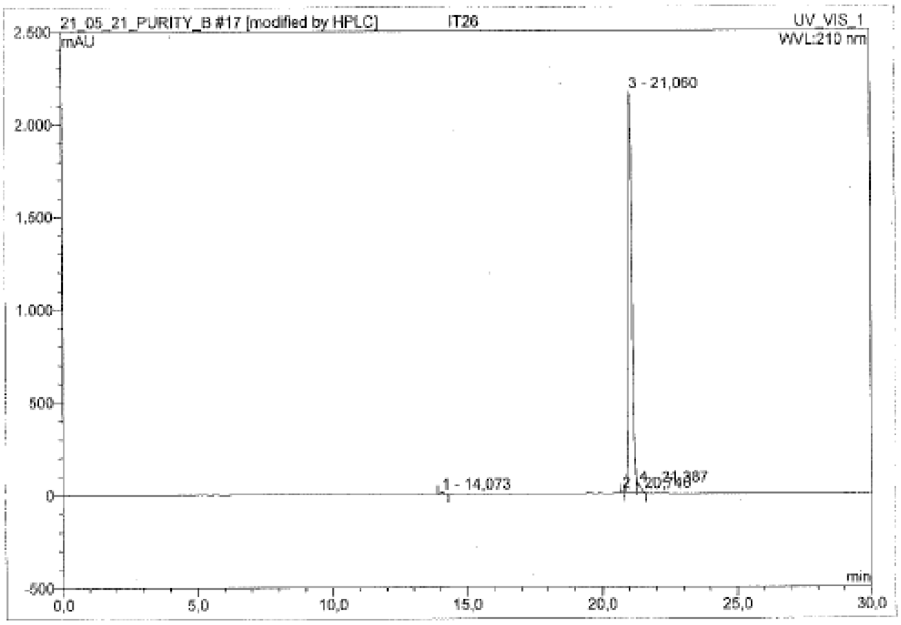

No.	Ret.Time min	Peak Name	Height mAU	Area mAU*min	Rel.Area %	Amount	Type
1	14,07	n.a.	8,671	1,502	0,40	n.a.	BMB*
2	20,74	n.a.	7,432	0,806	0,22	n.a.	BM *
3	21,06	n.a.	2163,574	364,791	97,57	n.a.	M *
4	21,39	n.a.	41,918	6,784	1,81	n.a.	MB*
Total:			2221,595	373,883	100,00	0,000	

HPLC analysis of **12a**

15 BP6

Sample Name:	BP6	*Injection Volume:*	5,0
Vial Number:	RC4	*Channel:*	UV_VIS_1
Sample Type:	unknown	*Wavelength:*	210
Quantif. Method:	Bioanalytik	*Dilution Factor:*	1,0000
Recording Time:		*Sample Weight:*	1,0000
Run Time (min):	30,00	*Sample Amount:*	1,0000

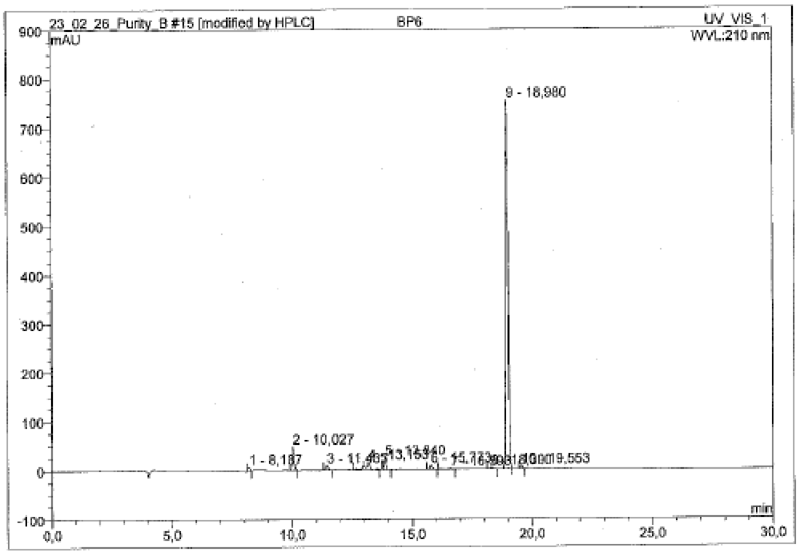

No.	Ret.Time min	Peak Name	Height mAU	Area mAU*min	Rel.Area %	Amount	Type
1	8,19	n.a.	7,343	0,645	0,67	n.a.	BMB*
2	10,03	n.a.	47,095	5,154	5,32	n.a.	BMB*
3	11,43	n.a.	8,980	1,016	1,05	n.a.	BMB*
4	13,15	n.a.	16,042	3,607	3,72	n.a.	BM *
5	13,84	n.a.	24,054	2,515	2,60	n.a.	BMB*
6	15,77	n.a.	8,738	0,960	0,99	n.a.	BMB*
7	16,59	n.a.	2,172	0,524	0,54	n.a.	BMB*
8	18,30	n.a.	3,299	0,369	0,38	n.a.	BMB*
9	18,98	n.a.	751,395	81,523	84,17	n.a.	BMB*
10	19,55	n.a.	5,396	0,547	0,56	n.a.	BMB*
Total:			874,516	96,860	100,00	0,000	

HPLC analysis of **12b**

17 BP7

Sample Name:	BP7	*Injection Volume:*	5,0
Vial Number:	RC5	*Channel:*	UV_VIS_1
Sample Type:	unknown	*Wavelength:*	210
Quantif. Method:	Bioanalytik	*Dilution Factor:*	1,0000
Recording Time:		*Sample Weight:*	1,0000
Run Time (min):	30,00	*Sample Amount:*	1,0000

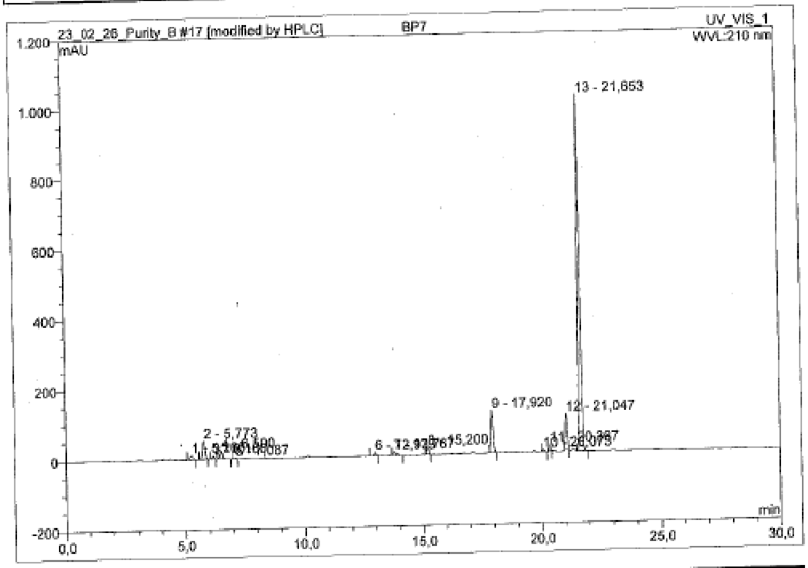

No.	Ret.Time min	Peak Name	Height mAU	Area mAU*min	Rel.Area %	Amount	Type
1	5,26	n.a.	11,350	1,834	1,21	n.a.	BMB*
2	5,77	n.a.	51,526	5,817	3,83	n.a.	BMB*
3	6,16	n.a.	7,517	0,790	0,52	n.a.	BMB*
4	6,50	n.a.	22,151	2,661	1,75	n.a.	BMB*
5	7,09	n.a.	2,699	0,271	0,18	n.a.	BMB*
6	12,97	n.a.	8,884	0,878	0,58	n.a.	BMB*
7	13,77	n.a.	9,703	1,405	0,93	n.a.	BMB*
8	15,20	n.a.	17,548	1,808	1,19	n.a.	BMB*
9	17,92	n.a.	119,555	12,906	8,50	n.a.	BMB*
10	20,07	n.a.	5,545	0,518	0,34	n.a.	BMB*
11	20,37	n.a.	18,379	1,754	1,16	n.a.	BMB*
12	21,05	n.a.	101,088	10,264	6,76	n.a.	BMB*
13	21,65	n.a.	1015,948	110,849	73,04	n.a.	BMB
Total:			1391,894	151,755	100,00	0,000	

HPLC analysis of **13a**

13 BP61

Sample Name:	**BP61**	*Injection Volume:*	**5,0**
Vial Number:	**RB5**	*Channel:*	**UV_VIS_1**
Sample Type:	**unknown**	*Wavelength:*	**210**
Quantif. Method:	**Bioanalytik**	*Dilution Factor:*	**1,0000**
Recording Time:		*Sample Weight:*	**1,0000**
Run Time (min):	**30,00**	*Sample Amount:*	**1,0000**

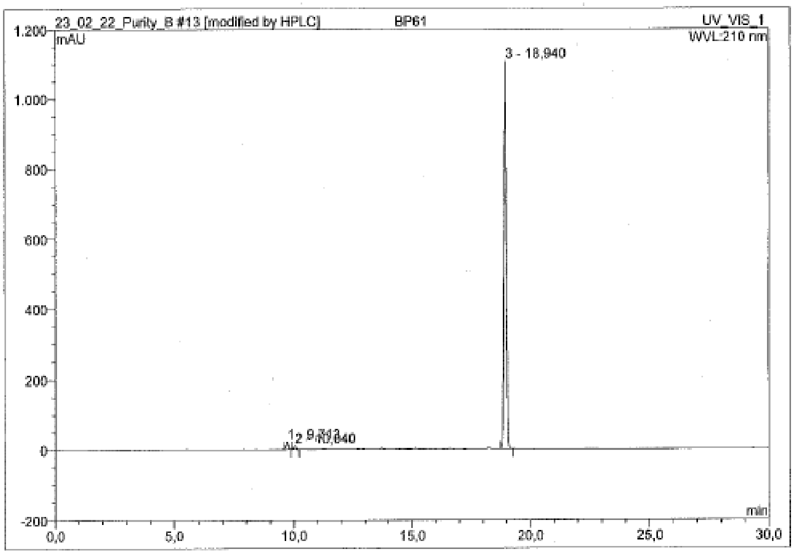

No.	Ret.Time min	Peak Name	Height mAU	Area mAU*min	Rel.Area %	Amount	Type
1	9,71	n.a.	22,347	2,391	1,94	n.a.	BMB*
2	10,04	n.a.	9,940	1,045	0,85	n.a.	BMB*
3	18,94	n.a.	1107,331	120,003	97,22	n.a.	BMB
**Total:**			1139,618	123,439	100,00	0,000	

HPLC analysis of **13b**

17 BP71

Sample Name:	BP71	*Injection Volume:*	5,0
Vial Number:	RB7	*Channel:*	UV_VIS_1
Sample Type:	unknown	*Wavelength:*	210
Quantif. Method:	Bioanalytik	*Dilution Factor:*	1,0000
Recording Time:		*Sample Weight:*	1,0000
Run Time (min):	30,00	*Sample Amount:*	1,0000

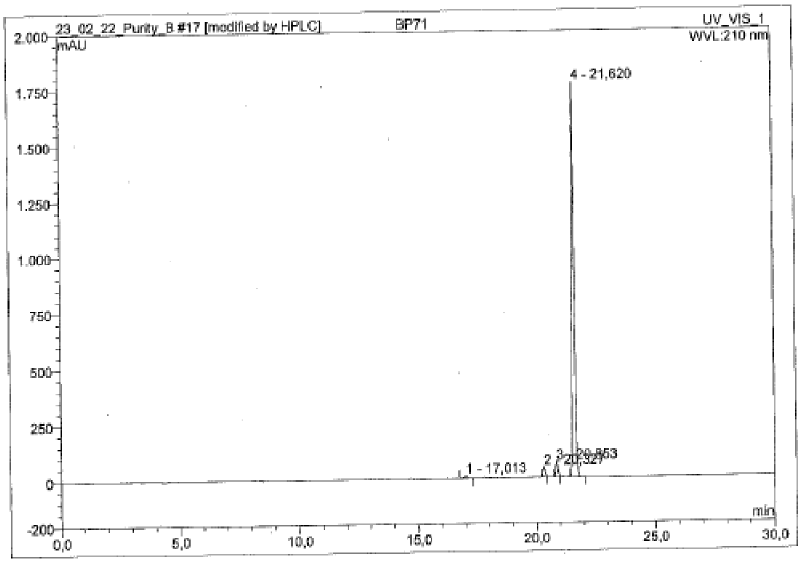

No.	Ret.Time min	Peak Name	Height mAU	Area mAU*min	Rel.Area %	Amount	Type
1	17,01	n.a.	9,829	1,397	0,67	n.a.	BMB*
2	20,33	n.a.	41,191	4,219	2,02	n.a.	BMB*
3	20,85	n.a.	65,069	6,804	3,25	n.a.	BMB*
4	21,62	n.a.	1766,036	196,914	94,07	n.a.	BMB
Total:			1882,125	209,334	100,00	0,000	

HPLC analysis of **14a**

15 BP63

Sample Name:	**BP63**	*Injection Volume:*	**5,0**
Vial Number:	**RB6**	*Channel:*	**UV_VIS_1**
Sample Type:	**unknown**	*Wavelength:*	**210**
Quantif. Method:	**Bioanalytik**	*Dilution Factor:*	**1,0000**
Recording Time:		*Sample Weight:*	**1,0000**
Run Time (min):	**30,00**	*Sample Amount:*	**1,0000**

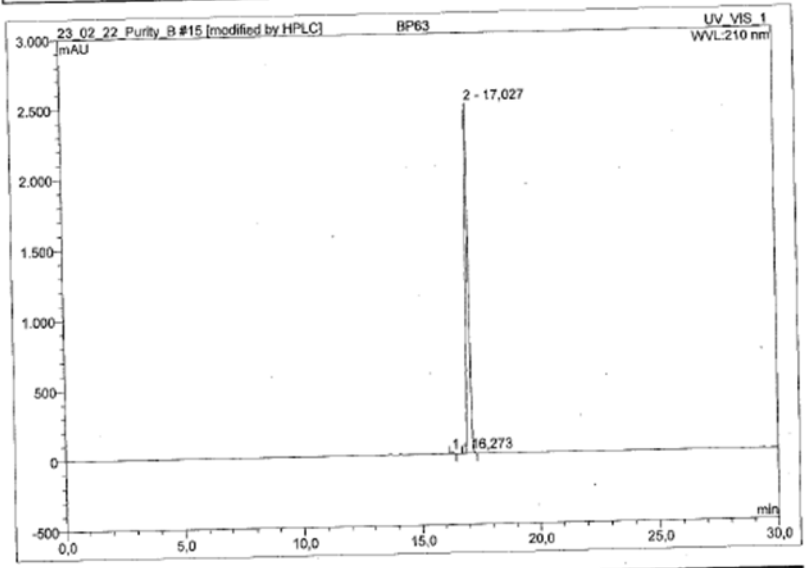

No.	Ret.Time min	Peak Name	Height mAU	Area mAU*min	Rel.Area %	Amount	Type
1	16,27	n.a.	15,474	1,855	0,53	n.a.	BMB*
2	17,03	n.a.	2498,231	346,564	99,47	n.a.	BMB
**Total:**			2513,705	348,419	100,00	0,000	

HPLC analysis of **14b**

13 BP72

Sample Name:	**BP72**	*Injection Volume:*	**10,0**
Vial Number:	**RC3**	*Channel:*	**UV_VIS_1**
Sample Type:	**unknown**	*Wavelength:*	**210**
Quantif. Method:	**Bioanalytik**	*Dilution Factor:*	**1,0000**
Recording Time:		*Sample Weight:*	**1,0000**
Run Time (min):	**30,00**	*Sample Amount:*	**1,0000**

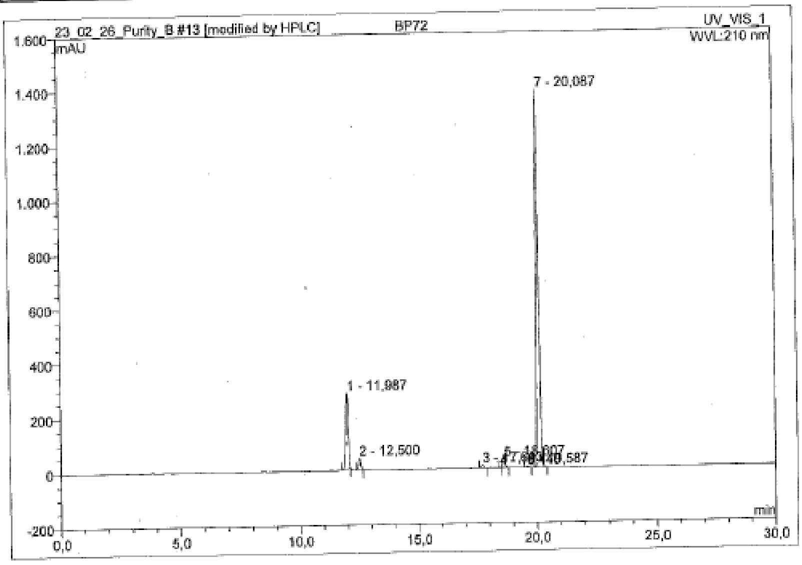

No.	Ret.Time min	Peak Name	Height mAU	Area mAU*min	Rel.Area %	Amount	Type
1	11,99	n.a.	277,033	32,439	15,51	n.a.	BMB*
2	12,50	n.a.	43,695	5,041	2,41	n.a.	BMB*
3	17,69	n.a.	11,319	1,310	0,63	n.a.	BMB*
4	18,44	n.a.	1,726	0,142	0,07	n.a.	BM *
5	18,61	n.a.	33,788	4,019	1,92	n.a.	MB*
6	19,59	n.a.	4,755	0,551	0,26	n.a.	BMB*
7	20,09	n.a.	1385,220	165,601	79,20	n.a.	BMB
**Total:**			1757,536	209,103	100,00	0,000	

HPLC analysis of **15a**

13 IT29

Sample Name:	**IT29**	*Injection Volume:*	**5,0**
Vial Number:	**RB6**	*Channel:*	**UV_VIS_1**
Sample Type:	**unknown**	*Wavelength:*	**210**
Quantif. Method:	**Bioanalytik**	*Dilution Factor:*	**1,0000**
Recording Time:		*Sample Weight:*	**1,0000**
Run Time (min):	**30,00**	*Sample Amount:*	**1,0000**

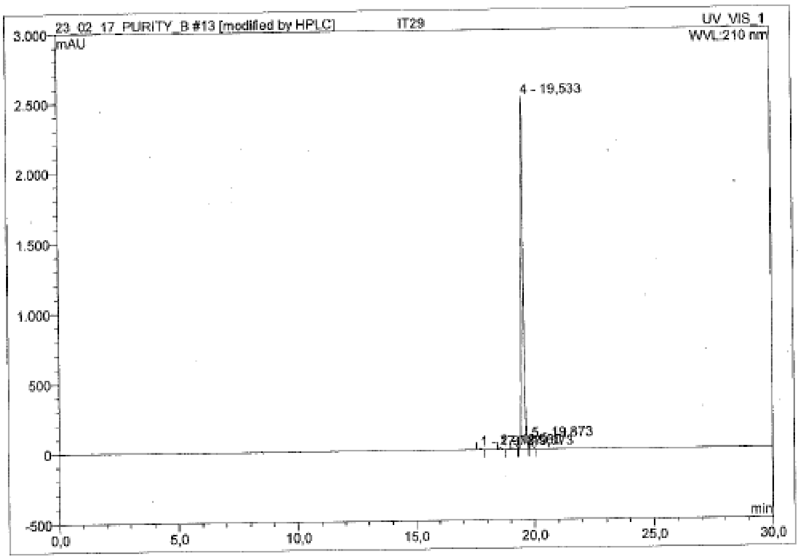

No.	Ret.Time min	Peak Name	Height mAU	Area mAU*min	Rel.Area %	Amount	Type
1	17,73	n.a.	10,174	1,045	0,31	n.a.	BMB*
2	18,56	n.a.	15,747	1,674	0,50	n.a.	BMB*
3	19,07	n.a.	7,321	0,833	0,25	n.a.	BM *
4	19,53	n.a.	2517,935	326,896	96,71	n.a.	M *
5	19,87	n.a.	68,950	7,570	2,24	n.a.	MB*
Total:			2620,127	338,018	100,00	0,000	

HPLC analysis of **16a**

19 IT30

Sample Name:	**IT30**	*Injection Volume:*	**5,0**
Vial Number:	**RC5**	*Channel:*	**UV_VIS_1**
Sample Type:	**unknown**	*Wavelength:*	**210**
Quantif. Method:	**Bioanalytik**	*Dilution Factor:*	**1,0000**
Recording Time:		*Sample Weight:*	**1,0000**
Run Time (min):	**30,00**	*Sample Amount:*	**1,0000**

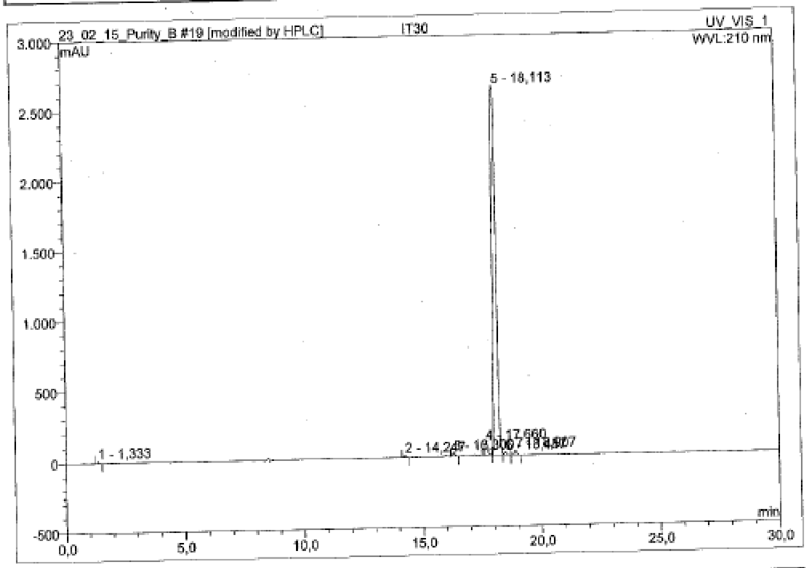

No.	Ret.Time min	Peak Name	Height mAU	Area mAU*min	Rel.Area %	Amount	Type
1	1,33	n.a.	14,716	0,946	0,20	n.a.	BMB*
2	14,25	n.a.	14,138	1,422	0,29	n.a.	BMB*
3	16,30	n.a.	24,894	2,607	0,54	n.a.	MB*
4	17,66	n.a.	93,098	11,485	2,37	n.a.	BM *
5	18,11	n.a.	2630,775	461,288	95,22	n.a.	M *
6	18,49	n.a.	21,479	2,847	0,59	n.a.	M *
7	18,91	n.a.	33,967	3,846	0,79	n.a.	MB*
Total:			2833,069	484,441	100,00	0,000	

HPLC analysis of **16b**

3 IT31

Sample Name:	IT31	*Injection Volume:*	10,0
Vial Number:	RB1	*Channel:*	UV_VIS_1
Sample Type:	unknown	*Wavelength:*	210
Quantif. Method:	Bioanalytik	*Dilution Factor:*	1,0000
Recording Time:		*Sample Weight:*	1,0000
Run Time (min):	30,00	*Sample Amount:*	1,0000

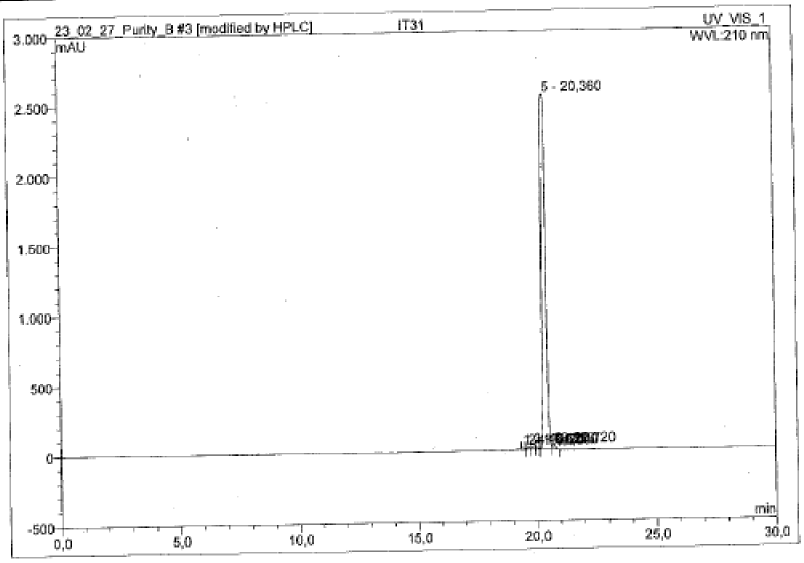

No.	Ret.Time min	Peak Name	Height mAU	Area mAU*min	Rel.Area %	Amount	Type
1	19,42	n.a.	16,495	1,733	0,34	n.a.	BM *
2	19,63	n.a.	17,569	2,450	0,48	n.a.	M *
3	19,85	n.a.	24,285	3,280	0,64	n.a.	M *
4	20,00	n.a.	15,859	1,979	0,38	n.a.	M *
5	20,36	n.a.	2539,385	500,482	97,31	n.a.	M *
6	20,72	n.a.	30,116	4,409	0,86	n.a.	MB*
Total:			2643,709	514,335	100,00	0,000	

HPLC analysis of **18a**

Chromatogram and Results

Injection Details

Injection Name:	**IT50**	*Run Time (min):*	**30,00**
Vial Number:	**GA2**	*Injection Volume:*	**5,00**
Injection Type:	**Unknown**	*Channel:*	**UV_VIS_1**
Calibration Level:		*Wavelength:*	**210,0**
Instrument Method:	**Chromni**	*Bandwidth:*	**n.a.**
Processing Method:	**Chromni Processing Method**	*Dilution Factor:*	**1,0000**
Injection Date/Time:	**19.Apr.23 16:43**	*Sample Weight:*	**1,0000**

Chromatogram

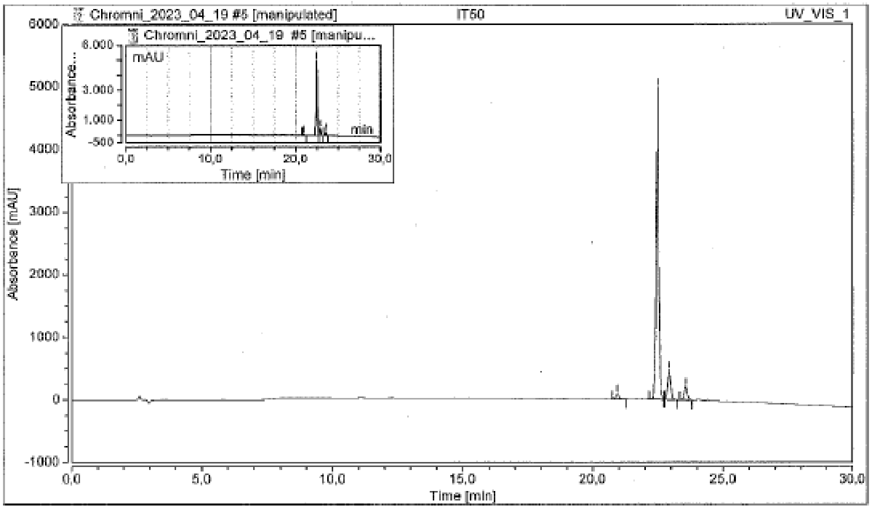

Integration Results

No.	Peak Name	Retention Time<br>min	Area<br>mAU*min	Height<br>mAU	Relative Area<br>%	Relative Height<br>%	Amount<br>n.a.
1		20,943	12,054	90,191	1,99	1,56	n.a.
2		22,482	501,464	4995,477	82,72	86,27	n.a.
3		22,923	63,298	461,031	10,44	8,31	n.a.
4		23,552	29,408	223,511	4,85	3,86	n.a.
**Total:**			**606,224**	**5790,209**	**100,00**	**100,00**	

Chromeleon (c) Dionex
Version 7.2.0.3765

HPLC analysis of **19b**

Instrument:AKBW_CHROMNI2 Sequence:Chromni_2023_04_12 Page 1 of 1

Chromatogram and Results

Injection Details

Injection Name:	**IT48**	*Run Time (min):*	**30,00**
Vial Number:	**GA7**	*Injection Volume:*	**5,00**
Injection Type:	**Unknown**	*Channel:*	**UV_VIS_1**
Calibration Level:		*Wavelength:*	**210,0**
Instrument Method:	**Chromni**	*Bandwidth:*	**n.a.**
Processing Method:	**Chromni Processing Method**	*Dilution Factor:*	**1,0000**
Injection Date/Time:	**12.Apr.23 19:59**	*Sample Weight:*	**1,0000**

Chromatogram

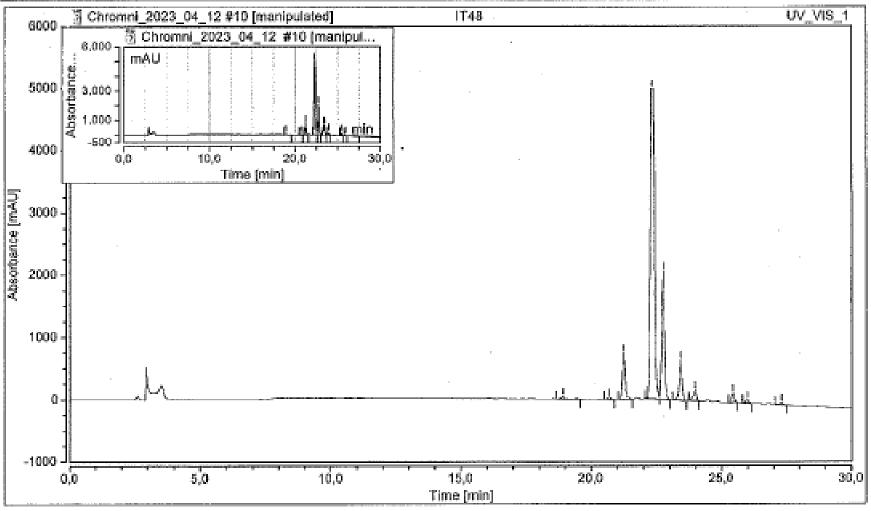

Integration Results

No.	Peak Name	Retention Time min	Area mAU*min	Height mAU	Relative Area %	Relative Height %	Amount n.a.
1		18,908	7,278	50,446	0,50	0,56	n.a.
2		20,673	4,579	41,176	0,31	0,46	n.a.
3		21,233	87,860	751,273	6,04	8,37	n.a.
4		22,307	965,565	4984,781	66,34	55,55	n.a.
5		22,737	250,998	2067,038	17,25	23,03	n.a.
6		23,405	85,124	651,039	5,85	7,25	n.a.
7		23,963	25,137	179,098	1,73	2,00	n.a.
8		25,422	17,413	153,516	1,20	1,71	n.a.
9		25,972	7,809	60,621	0,54	0,68	n.a.
10		27,307	3,639	34,803	0,25	0,39	n.a.
**Total:**			**1455,402**	**8973,791**	**100,00**	**100,00**	

Default/Integration

Chromeleon (c) Dionex
Version 7.2.0.3765

HPLC analysis of **21a**

Instrument:AKBW_CHROMNI2 Sequence:Chromni_2023_04_19 Page 7 of 28

Chromatogram and Results

Injection Details

Injection Name:	**IT53**	*Run Time (min):*	**30,00**
Vial Number:	**GA4**	*Injection Volume:*	**5,00**
Injection Type:	**Unknown**	*Channel:*	**UV_VIS_1**
Calibration Level:		*Wavelength:*	**210,0**
Instrument Method:	**Chromni**	*Bandwidth:*	**n.a.**
Processing Method:	**Chromni Processing Method**	*Dilution Factor:*	**1,0000**
Injection Date/Time:	**19.Apr.23 18:05**	*Sample Weight:*	**1,0000**

Chromatogram

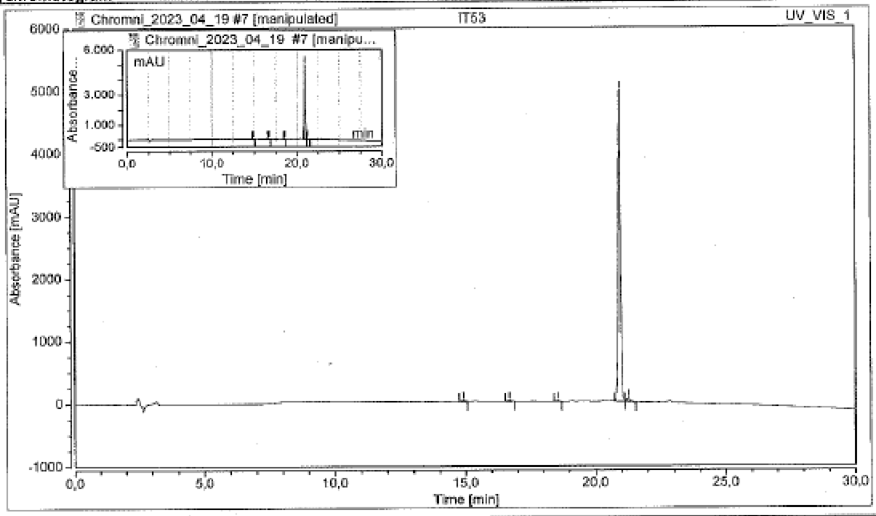

Integration Results

No.	Peak Name	Retention Time min	Area mAU*min	Height mAU	Relative Area %	Relative Height %	Amount n.a.
1		14,902	2,204	22,122	0,50	0,43	n.a.
2		16,692	2,706	24,589	0,62	0,48	n.a.
3		18,553	2,270	22,702	0,52	0,44	n.a.
4		20,943	420,597	4981,161	96,27	97,27	n.a.
5		21,248	9,098	70,312	2,08	1,37	n.a.
**Total:**			**436,874**	**5120,886**	**100,00**	**100,00**	

Default/Integration

Chromeleon (c) Dionex
Version 7.2.0.3765

HPLC analysis of **21b**

Chromatogram and Results

Injection Details

Injection Name:	IT52	*Run Time (min):*	30,00
Vial Number:	BA6	*Injection Volume:*	5,00
Injection Type:	Unknown	*Channel:*	UV_VIS_1
Calibration Level:		*Wavelength:*	210,0
Instrument Method:	Chromni	*Bandwidth:*	n.a.
Processing Method:	Chromni Processing Method	*Dilution Factor:*	1,0000
Injection Date/Time:	14.Jul.23 02:05	*Sample Weight:*	1,0000

Chromatogram

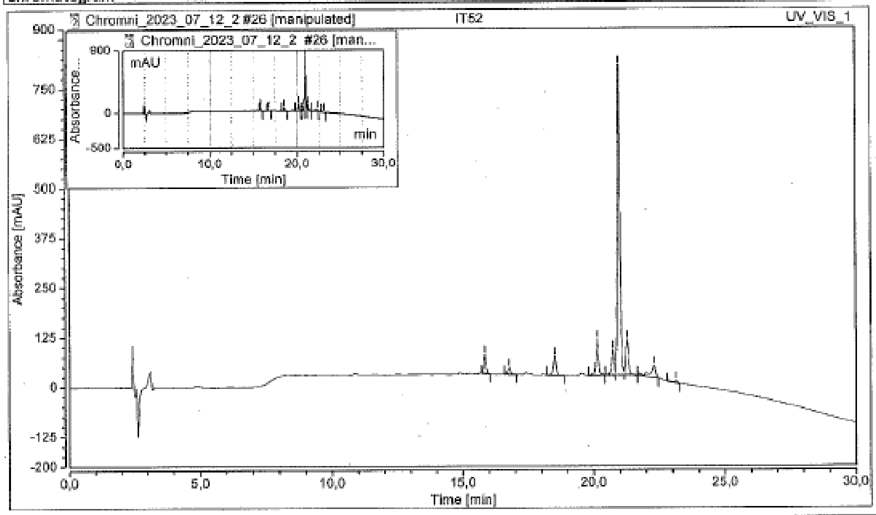

Integration Results

No.	Peak Name	Retention Time min	Area mAU*min	Height mAU	Relative Area %	Relative Height %	Amount n.a.
1		15,838	4,591	48,078	3,55	4,07	n.a.
2		16,760	1,856	16,566	1,43	1,40	n.a.
3		18,520	5,248	48,667	4,06	4,12	n.a.
4		20,137	10,737	92,530	8,30	7,84	n.a.
5		20,723	7,131	64,634	5,51	5,48	n.a.
6		20,973	81,991	780,614	63,39	66,15	n.a.
7		21,272	9,943	90,547	7,69	7,67	n.a.
8		22,298	6,872	31,804	5,31	2,70	n.a.
9		23,120	0,984	6,566	0,76	0,56	n.a.
**Total:**			**129,352**	**1180,006**	**100,00**	**100,00**	
